# Influence of Ligand Design and Non‐Covalent Interactions on the Isoselective Ring‐Opening Polymerization of *rac*‐β‐Butyrolactone Using Salan and Salalen Rare‐Earth Metal Catalysts

**DOI:** 10.1002/anie.202504513

**Published:** 2025-05-06

**Authors:** Stefanie Hörl, Ion Chiorescu, Jonas Bruckmoser, Jonas Futter, Bernhard Rieger

**Affiliations:** ^1^ Wacker‐Lehrstuhl für Makromolekulare Chemie Catalytic Research Center Technische Universität München Lichtenbergstraße 4 85748 Garching Germany; ^2^ Department Chemie Technische Universität München Lichtenbergstraße 4 85748 Garching Germany

**Keywords:** DFT calculations, Isoselective ring‐opening polymerization, Non‐covalent interactions, Polyhydroxybutyrate, Salan/salene complexes

## Abstract

We herein report the influence of the ligand framework on the isoselective ring‐opening polymerization (ROP) of *rac*‐β‐butyrolactone using highly active in situ generated salan [ONNO]^H2^ and salalen [ONNO]^H^ rare‐earth metal complexes. The stereochemistry was found to be highly dependent on the *ortho*‐substituents, enabling the synthesis of either isotactic poly(3‐hydroxybutyrate) (PHB) (up to *P_m_* = 0.92) or syndiotactic PHB (up to *P_r_* = 0.91). To obtain further information on the mechanism of the isoselective ROP, which exhibits an enantiomorphic site control (ESC), a new hybrid Y[ONNO]^H^(N(SiHMe_2_)_2_)(THF) complex was isolated and characterized via NMR, DFT, and mass‐spectrometry experiments. The impact of non‐covalent interactions (NCIs) was demonstrated by the addition of NCI inhibitors. Kinetic studies revealed a secondary kinetic isotope effect (SKIE) of 1.14, indicating that the pre‐coordination of the monomer plays a significant role in the mechanism. These findings provide a foundation for the future design of catalysts for isoselective ROP.

## Graphical abstract

Polyhydroxybutyrates are key sustainable polyesters, but controlling the stereoregularity is crucial for exhibiting the most promising thermal and mechanical properties. Highly active rare‐earth metal salan/salalen complexes yield isotactic (*P_m_* up to 0.96 at 0 °C) or syndioenriched (*P_r_* up to 0.91) PHB, depending on the steric demand of the ligand and non‐covalent interactions (NCIs) in the catalytic system.

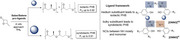

With fossil fuel depletion and the growing concern about plastic pollution, the search for bio‐based and biodegradable polymers has gained popularity in both academic research and industrial development.^[^ ^1^, ^2^ ^]^ A well‐known example of a polymer with a promising end‐of‐life scenario is polyhydroxyalkanoate, which is biologically accessible via fermentative processes. However, they have the disadvantage of high production costs and challenging process scaling and downstream purification.^[^ ^2^, ^3^, ^4^, ^5^ ^]^ Within this class of polymers, poly(3‐hydroxybutyrate) (PHB) is the most promising polyester as it exhibits excellent thermal properties (*T_m_* = 180 °C). The properties are induced by the configuration of the methyl group, as naturally, only a strictly isotactic (*R*) configuration of the chiral center occurs (probability of adjacent *meso* linkage *P_m_* = 1).^[^ ^6^, ^7^ ^]^

To overcome scalability and cost problems, synthetic approaches towards PHB were subject to research over the past 60 years via ring‐opening polymerization (ROP) of the inexpensive and commercially available monomer *rac‐*β‐butyrolactone (BBL).^[^ ^8^, ^9^, ^10^, ^11^ ^]^ The monomer is accessible via the carbon monoxide insertion into propylene oxide and can be considered a sustainable feedstock since propylene production can be CO_2_‐based (Figure 1).^[^ ^8^, ^12^, ^13^, ^14^, ^15^ ^]^ Access to syndiotactic PHB was already obtained in 2006 as Carpentier and coworkers investigated a yttrium aminoalkoxy(bisphenolate) complex, which is highly stereoselective.^[^ ^16^, ^17^ ^]^ While syndioselective catalysts have received less attention in past years, new syndioselective spiro‐salen catalysts were recently published, along with applications of syndio‐rich PHB as excellent adhesives offering a sustainable alternative.^[^ ^18^, ^19^, ^20^ ^]^ Isoselective ROP, however, was highly challenging, and initially, only catalysts accessing isoenriched PHB, like chromium salophene complexes (*P_m_* = 0.67)^[^ ^21^, ^22^, ^23^ ^]^ rare‐earth metal‐based complexes (*P_m_* up to 0.75),^[^ ^24^, ^25^, ^26^ ^]^ and a heterogeneous neodymium borohydride supported on silica catalyst (*P_m_* = 0.85)^[^ ^27^ ^]^ were known. Another approach was published by Chen and coworkers, who obtained highly isotactic PHB via the synthesis of an eight‐membered diolide and subsequent ROP, accepting the trade‐off of multistep monomer synthesis.^[^ ^28^, ^29^, ^30^ ^]^

**Figure 1:**
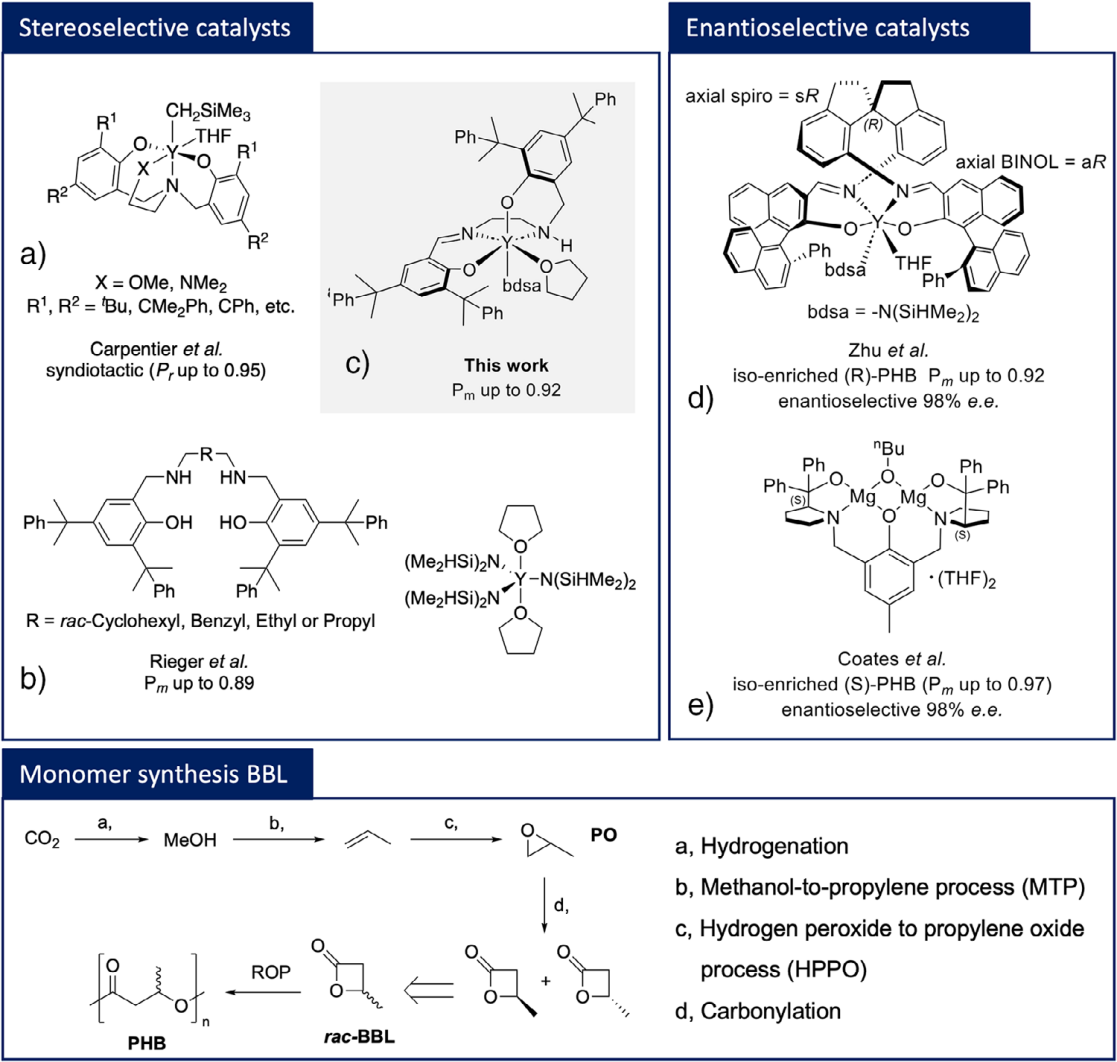
Examples of reported stereo‐ and enantioselective catalysts for the stereoselective ROP of *rac*‐BBL and sustainable CO_2_‐based monomer synthesis.^[^ ^12^, ^15^, ^31^, ^32^, ^33^ ^].^

The strategy of kinetic resolution polymerization of the racemic mixture has recently gained importance as two enantioselective catalysts have been published, yielding highly isotactic PHB under the influence of an enantiomorphic site control (ESC) (Figure 1). Coates and coworkers reported a bimetallic (*S*,*S*)‐prophenolMg_2_(μ‐O*^n^* Bu)(THF)_2_ catalyst that produces nearly perfectly isotactic (*S*)‐PHB (*P_m_* = 0.98) via ring‐opening of (*R*)‐BBL while inverting the stereocenter.^[^ ^34^ ^]^ While the earth‐abundant catalyst is limited to the polymerization of only one enantiomer, Zhu and coworkers could show the activity of a C_2_ symmetric spiro‐BINOL salen yttrium catalyst for either the (*R*) or the (*S*) monomer, respective to its stereoinformation (*P_m_* values up to 0.92).^[^ ^35^ ^]^ It should be noted that these approaches always lead to ∼50% conversion, and an additional use for the remaining monomer is needed. To overcome this problem, our group recently reported a catalyst bearing an amine functionality in the ligand that simultaneously polymerizes both monomers while still maintaining a high isospecificity. The introduction of these salan pro ligands in rare‐earth metal catalyzed ROP provided the possibility of a further coordination sphere in the active species by hydrogen bond donors (NH).^[^ ^36^ ^]^

The importance of these non‐covalent interactions (NCI) introduced by the NH moieties has already been thoroughly investigated by Romain and coworkers using aluminum salan complexes.^[^ ^37^ ^]^ Carpentier and coworkers also demonstrated the major role of NCIs in the stereoselective ROP of 4‐alkoxymethylene‐β‐propiolactones (BPL^OR^). These studies nicely show how important the design of the ligand sphere is for the ring‐opening mechanism in stereoregular PHAs.^[^ ^38^, ^39^, ^40^, ^41^, ^42^ ^]^

We herein report the influence of catalyst design in terms of *ortho*‐substituents and NCIs in the stereoselective ROP of *rac*‐BBL generating either syndiotactic or isotactic PHB with high TOFs. These studies are providing a deeper understanding of characteristic features for stereoselective polymerization. Varying the *ortho*‐substituent and introducing functional groups can have a major impact on the stereocontrol. Recently, we could highlight the screening ability of the in situ approach with yttrium salan complexes, obtaining mechanistic details and information without time‐consuming purification steps.^[^ ^43^ ^]^ Using salan pro‐ligands [ONNO]^H2^ with a *racemic* cyclohexyl and propyl backbone led to iso‐enriched PHB up to 0.89 (at −35 °C) in both cases. Besides the backbone, the *ortho*‐substituents also showed a major impact, as a small group like *^t^* butyl **(L1^Cyclohexyl^**) led to an atactic PHB in comparison to a dicumyl group (**L2^Cyclohexyl^**) (Table 1, entry 1 vs 2; Figure 2).^[^ ^36^ ^]^

**Table 1:** Ring‐opening polymerization of *rac*‐BBL with various yttrium‐salan and salalan complexes.^a)^

			*t*_R_	*X_P_* ^b)^	*M_n_*_,rel_ ^c)^		
Entry	Catalytic System	(M):(Cat)	(min)	(%)	(kg mol^−1^)	*Ð* ^c)^	*P_m_* ^d)^
1^e)^	**Y+L1^Cyclohexyl^**	1:200	120	83	35	1.9	0.63
2^e)^	**Y+L2^Cyclohexyl^**	1:200	1	98	41	2.2	0.82
3	**Y+L3^Cyclohexyl^**	1:200	1	99	47	1.9	0.83
4	**Y+L4^Cyclohexyl^**	1:200	3	99	43	3.0	0.09
5^e)^	**Y+L2^Propyl^**	1:200	7	81	148	1.9	0.88
6	**Y+L3^Propyl^**	1:200	7	97	145	2.2	0.90
7	**Y+L4^Propyl^**	1:200	7	75	99	1.9	0.28
8	**Y+L5**	1:200	30	55	43	1.9	0.92
9	**YL5**	1:50	20	82	13	1.6	0.89
10	**YL5**	1:200	20	72	43	1.8	0.91
11^f)^	**YL5**	1:200	120	94	23	1.8	0.81
12^g)^	**YL5**	1:200	480	87	126	1.6	0.96

^a)^ Polymerization conditions: [BBL]_0_ = 2.0 M in toluene at rt.

^b)^ Determined via ^1^H NMR spectroscopy in CDCl_3_.

^c)^ Determined via SEC in CHCl_3_ at 40 °C relative to polystyrene calibration.

^d)^ Determined via ^13^C{^1^H} NMR spectra in CDCl_3_, integration of the carbonyl signal.

^e)^ Values from literature.^[^ ^36^ ^]^

^f)^ Polymerization conducted at 75 °C.

^g)^ Polymerization conducted at 0 °C.

**Figure 2:**
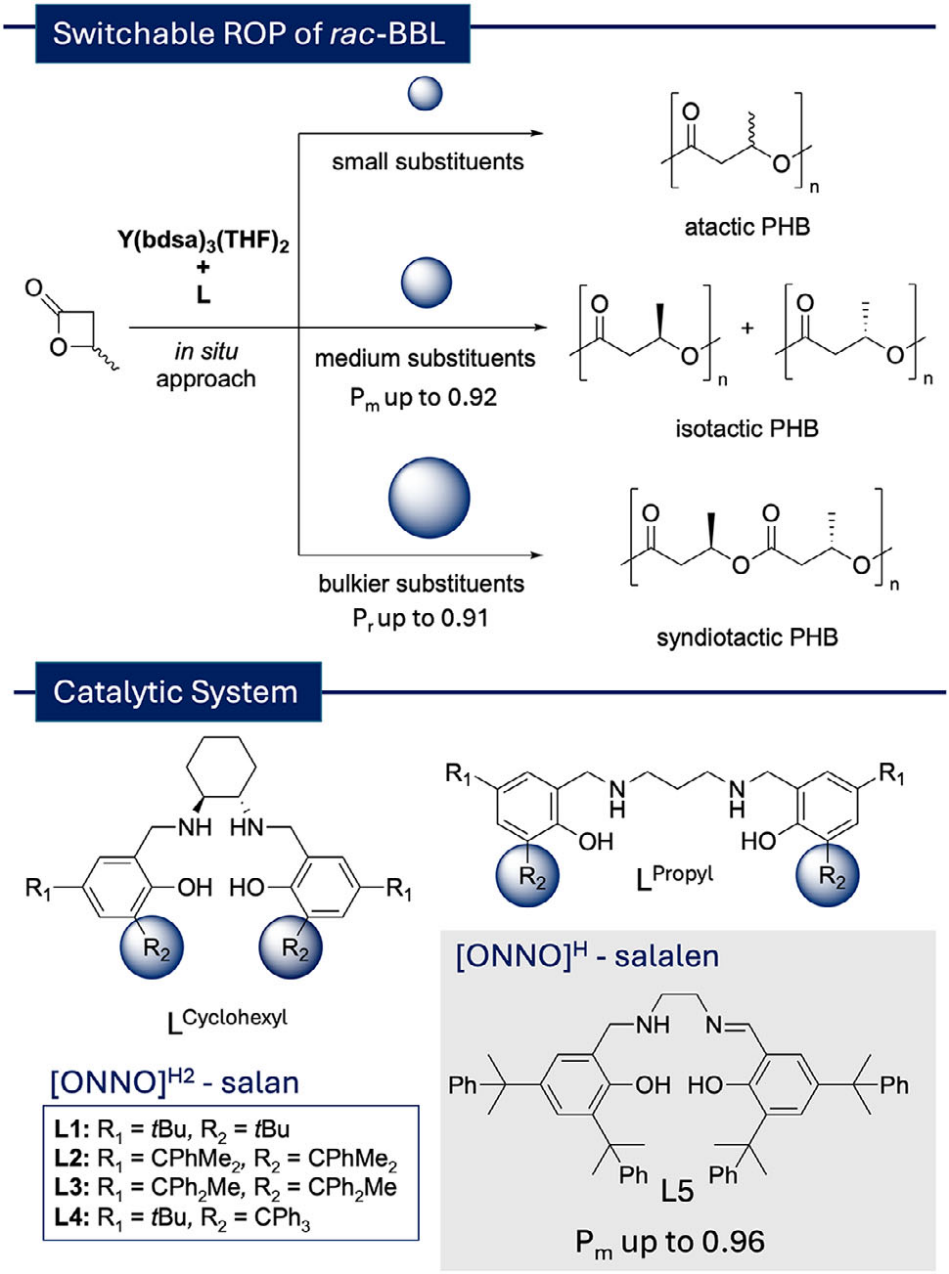
Stereoselective ring‐opening polymerization of *rac*‐BBL using in situ‐generated yttrium salan complexes yielding different tacticities depending on the *ortho*‐substituent (bdsa = N(SiHMe_2_)_2_).

We, therefore, increased the steric demand by one additional phenyl group of the pro‐ligand (**L3^Cyclohexyl^**) to further optimize the isoselectivity and obtained a *P_m_* value of 0.83 while still yielding high molecular weights of 47 kg mol^−1^ (Table 1, entry 3). Surprisingly, if the remaining methyl group was replaced by a third phenyl moiety, a switch in the polymerization mechanism occurred from an ESC to a chain‐end control (CEC) (Figures 3f vs g and S25), resulting in a highly syndiotactic PHB (*P_r_* = 0.91) (Table 1, entry 4). The same tendencies of increased isotacticity with enhanced steric demand in the *ortho*‐position can be found when comparing the two different pro‐ligands **L2^Propyl^** and **L3^Propyl^** (Table 1, entries 5–7). **L4^Propyl^**, on the other hand, also resulted in syndio‐enriched PHB (*P_r_* = 0.72) due to a CEC. Kinetic analysis revealed a first‐order dependence of the polymerization rate on BBL after a prolonged initiation period of 2 min, compared to 0.7 min for **L2^Propyl^**.^[^ ^36^ ^]^ This study shows nicely that the change in the mechanism affects only the stereoselectivity and has no impact on the polymerization kinetics itself (Figure S20). Similar switchable polymerization behavior of alkylated salan rare‐earth metal catalysts in ROP of *rac*‐BBL was published by the group of Cui, hypothesizing stereoelectronic effects accountable for the stereoselectivity.^[^ ^26^ ^]^

**Figure 3:**
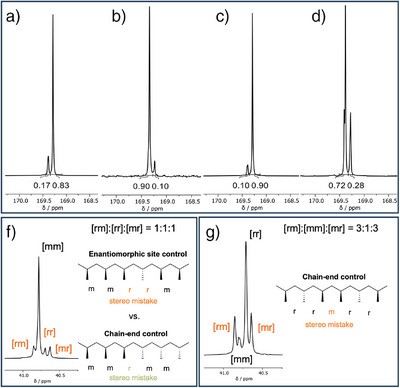
^13^C{^1^H} NMR spectra (carbonyl region) of isotactic and syndiotactic PHBs produced by a), **Y+L3^Cyclohexyl^** b), **Y+L4^Cyclohexyl^** c), **Y+L3^Propyl^** d), **Y+L4^Propyl^**. Switch in mechanism observable in the methylene region of PHB produced by f) **Y+L3^Cyclohexyl^** and g) **Y+L4^Propyl^**.

We however, hypothesized the presence of a catalytic pocket in which an ESC mechanism and coordination of the monomer unit occurred. Increasing the steric demand led to fewer stereo mistakes, but if the catalytic pocket is getting too crowded by big *ortho*‐substituents, no pre‐coordination of the monomer takes place, resulting in the change of selectivity. We already could show that salen pro‐ligands are not isoselective,^[^ ^36^ ^]^ and we were thus interested if a hybrid salalen pro‐ligand structure could still produce isotactic PHB. For the class of aluminum catam catalysts, which are known to be highly active for the related stereoselective ROP of *rac*‐lactide, hybrid catalen catalyst, for example are resulting in a reduced activity.^[^ ^44^, ^45^ ^]^

With the exchange of one amine functionality by an imine in the ligand backbone, we aimed to introduce a more rigid moiety while still retaining an NH functional group necessary for the stereoselective ROP, according to our hypothesis. The synthesis pathway of the tetradent salalen ([ONNO]^H^) pro‐ligand started with a condensation reaction of dicumylsalicylaldehyde and ethylenediamine, directly followed by a reduction step. The subsequent condensation led to the asymmetric salalen ligand **L5** (Scheme S1). The synthesis of the yttrium salalen complex **YL5** was achieved by a metathesis reaction with Y(bdsa)_3_(THF)_2_ in a similar procedure to various salen catalysts in literature.^[^ ^28^, ^46^, ^47^ ^]^

Slow precipitation in pentane gave analytically pure Y[ONNO]^H^(N(SiHMe_2_)_2_)(THF) catalyst **YL5**, which was fully characterized by ^1^H and ^13^C{^1^H} NMR spectroscopy (Figures S30–S35) and elemental analysis. The ^1^H NMR (C_6_D_6_) shows the characteristic pentet for the bdsa group (4.74 ppm) as well as the signals for a coordinated THF (3.20 ppm), leading in combination with the tetradent [ONNO]^H^ ligand to an octahedral geometry (Figure 4). No suitable single crystals for X‐crystallography were obtained, but diffusion‐ordered NMR spectroscopy (DOSY) (Figure S36),^[^ ^48^ ^]^ and liquid injection field desorption/ionization mass spectrometry analysis (LIFDI‐MS) could detect a monomeric species (929.2 g mol^−1^). A minor fraction of a dimeric species with a molecular weight of 1657.7 g mol^−1^ (Y_2_[ONNO]^H^_2_) was detected, along with a Y_2_[ONNO]^H^_3_ complex, which is most probably inactive in the polymerization process (Figure S37).

**Figure 4:**
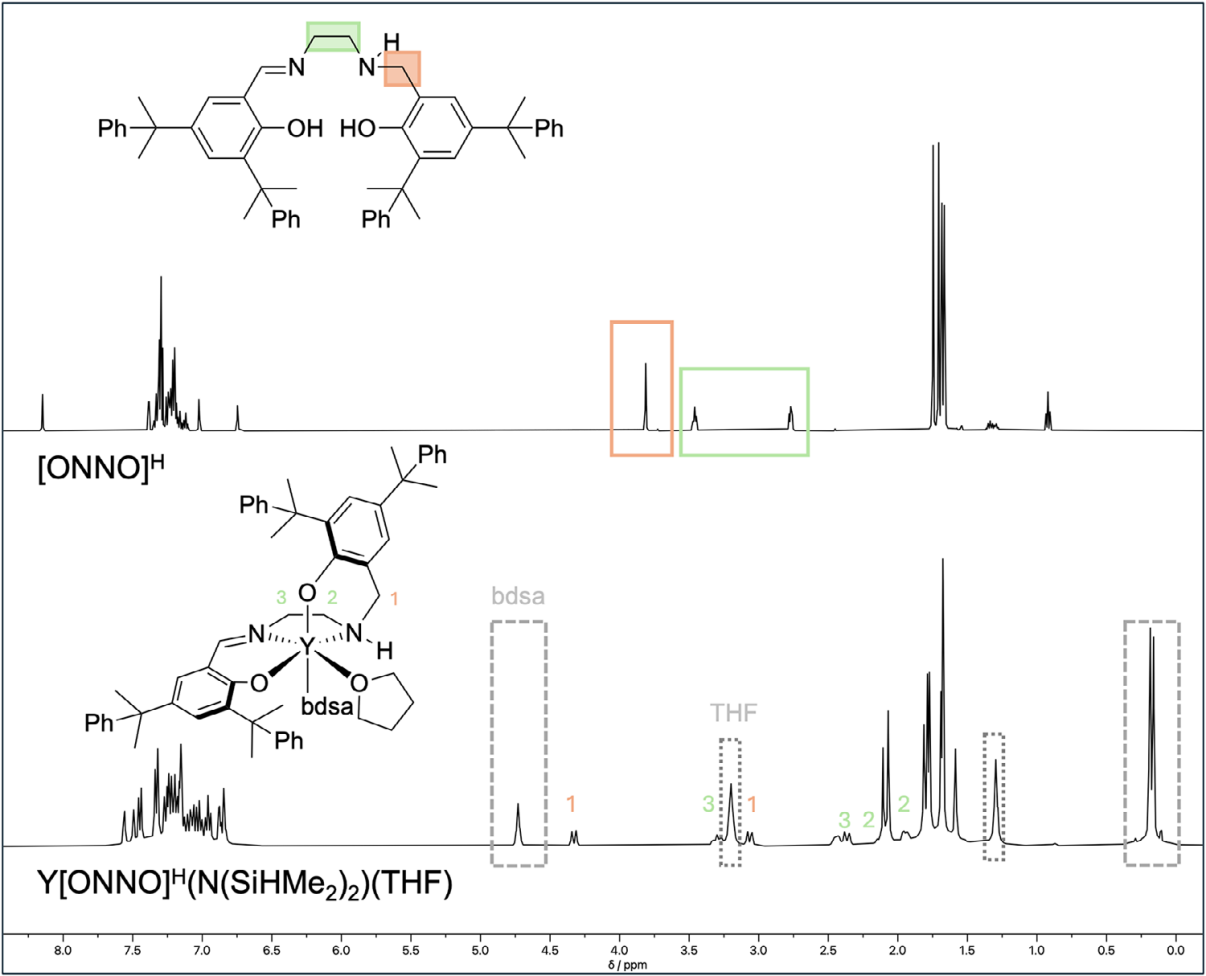
^1^H NMR spectrum of pro‐ligand **L5** (upper) and isolated **YL5** (lower), both measured in C_6_D_6_ at rt.

While salan and salen complexes typically exhibit a binding modus of either *fac–fac* or *mer–mer*, respectively, the unsymmetrical salalen pro‐ligands are known to bind in either *fac*‐*mer* or *mer*‐*fac*.^[^ ^49^ ^]^ DFT calculations confirm *fac‐mer* as the most stable configuration (Figure 5). All others are significantly less favored; the next most stable, *mer–mer*, is disfavored by 19 kJ mol^−1^. A detailed analysis of the computed stability is provided in the Supporting Information.

**Figure 5:**
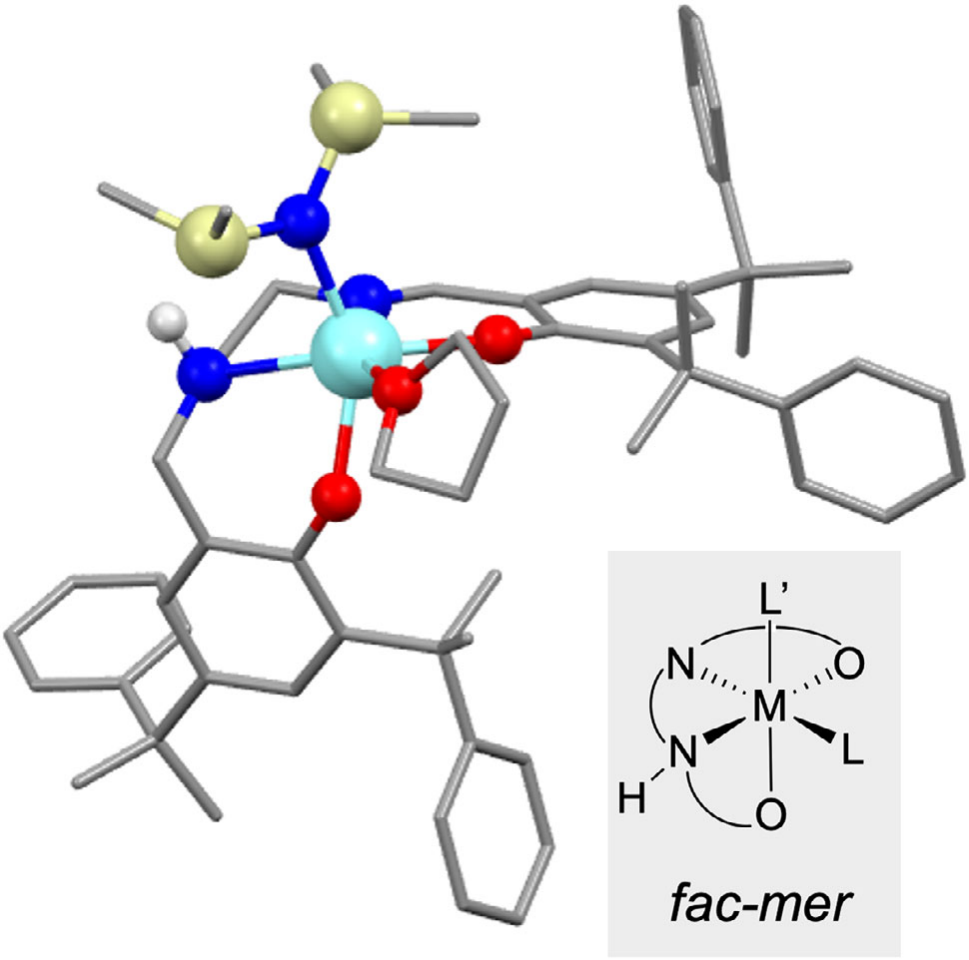
The most stable structure of **YL5**. All hydrogen atoms, except those in the amine moiety, are omitted for clarity.

First, we tested pro‐ligand **L5** in the in situ approach polymerizing *rac*‐BBL. While the polymerization activity is decreased and after 20 min strong gelation of the reaction mixture occurs, a high *P_m_* of 0.92 was obtained. Evaluation of the conversion showed a stagnation of the polymerization at 55% and molecular weights of 43 kg mol^−1^. To exclude the possibility of a kinetic resolution polymerization of one enantiomer, polymerization experiments with both (*R*)‐ and (*S*)‐BBL (Table S1, entries 1 and 2) and an investigation of the unreacted monomer surplus via chiral GC‐MS (Figure S26) were conducted. Both enantiomers exhibited polymerizability, indicating that the catalyst is not enantioselective. Using the isolated catalyst **YL5**, the ROP of *rac*‐BBL proceeded similarly to the in situ approach. The conversion was slightly higher at around 72%, with molecular weights of 66 kg mol^−1^ and a high isotacticity (*P_m_* = 0.91) (Table 1, entry 10). A lower [BBL]/[**YL5**] ratio of 50:1 resulted in molecular weights of 13 kg mol^−1^, demonstrating the possibility of controlling molecular weights (Table 1, entry 9). Polymerization at higher temperatures resulted in complete conversion; however, this was accompanied by a reduced isotacticity (*P_m_* = 0.81, Table 1, entry 11), while decreasing the temperature to 0 °C resulted in almost perfectly isotactic PHB (*P_m_* = 0.96, Table 1, entry 12).

We hypothesized that the coordination of the monomer in the catalytic pocket for our catalytic system is the crucial step for achieving a highly stereoselective ROP. Such NCIs between a monomer and an NH functionality were already reported in the stereoselective ROP of *rac‐*lactide.^[^ ^37^ ^]^ If alkylated salan ligands^[^ ^43^ ^]^ [ONNO]^R2^ or salen ligands^[^ ^36^ ^]^ [ONNO] are used in the polymerization of *rac‐*BBL, no stereocontrol over tacticity is achieved due to their inability to form NCIs (Scheme S2). We additionally performed two different experiments underlying this hypothesis. At first, an NCI blocker, like benzyl alcohol (BnOH), was added. As expected, the isotacticity decreased, resulting in an atactic polymer (Table S1, entries 3 and 4). As a second approach, kinetic isotope experiments were performed to gain further information on the mechanism in the rate‐determined step (KIE = *k* ^H^/*k* ^D^). A primary kinetic isotope effect (KIE) can be seen if the isotopic substitution occurs in a bond‐breaking or forming (PKIE >> 1). Secondary KIEs are usually lower and can be seen if the internal vibration of the system is influenced, e.g., a change in hybridization (SKIE ∼1.1–1.2 or, if inversed, ∼0.8–0.9).^[^ ^50^, ^51^, ^52^ ^]^ We therefore deuterated **L5** using D_2_O to obtain **L5^D^** and subsequently used it as a pro‐ligand in the polymerization. Polymerization with **L5^D^** was well controlled (*M_n_*_,rel_ = 57–59 kg mol^−1^, *Ð* = 1.6–1.8), resulting in isotactic PHB (Table S2, entries 3 and 4). Determination of the rate constant *k*_obs_ showed two active catalysts, with a *k* ^H^_obs_ = 4.48 ± 0.18·10^−3^ s^−1^ for **L5** and *k* ^D^_obs_ = 3.92 ± 0.05·10^−3^ s^−1^ for **L5^D^** (Figure 6), meaning that the deuterated “heavier” polymerization is 1.14 times slower than **L5**. These results indicate a secondary kinetic isotope effect (SKIE) in which no bond breaking is observed but rather coordination of the monomer to the NH/ND moieties occurs in the rate‐determining step.^[^ ^53^ ^]^

**Figure 6:**
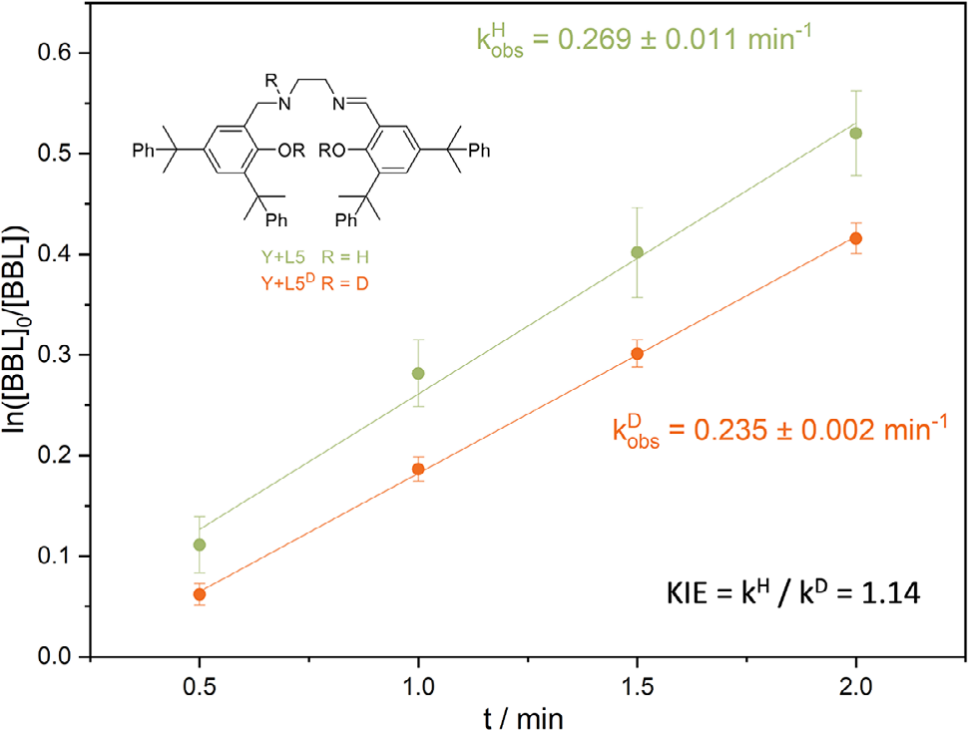
Plot of *k* ^H^_obs_ (**Y+L5**, green) and *k* ^D^_obs_ (**Y+L5^D^**, orange) showing the kinetic isotope effect observed in the stereoselective ROP of *rac*‐BBL.

In summary, we could show the important influence of the *ortho*‐substituent in the stereoselective ROP, and we could demonstrate that a hybrid salalen ligand [ONNO]^H^, which combines the stability of an imine moiety with the isoselectivity of the amine moiety, is a highly selective and active catalyst in the ROP of *rac*‐BBL. Regarding the mechanism, a switch from isotactic to syndiotactic PHB occurred due to the blockade of the catalytic pocket by sterically demanding trityl moieties. The mechanism responsible for the isospecificity could be identified through experiments with NCI inhibitors and kinetic studies using a deuterated salalen ligand. The latter revealed a secondary KIE at the NH moiety, suggesting monomer coordination with the hydrogen in the rate‐determining step. Additionally, we could synthesize a new yttrium salalen catalyst, **YL5**, demonstrating high activity in the isoselective polymerization of *rac*‐BBL with *P_m_* up to 0.92. DFT calculation confirmed a *fac‐mer* configuration to be the most stable. Overall, structural characterization and kinetic studies could show the importance of the ligand framework and the presence of hydrogen bond donors in stereoselective polymerizations. Exploiting such interactions opens up new avenues for the future catalyst as well as polymer design, eventually delivering materials with tailored high‐performance properties.

## Supporting Information

Additional experimental details, materials, and methods; additional polymerization data; ^1^H and ^13^C{^1^H} NMR spectra for all novel compounds (PDF).

## Conflict of Interests

The authors declare no conflict of interest.

## Supporting information



Supporting Information

## Data Availability

The data that support the findings of this study are available in the supplementary material of this article.
