# Supplementary material for: Influence of Ligand Design and Non‐Covalent Interactions on the Isoselective Ring‐Opening Polymerization of rac‐β‐Butyrolactone Using Salan and Salalen Rare‐Earth Metal Catalysts
Source: Angew Chem Int Ed Engl. 2025 May 6;64(26):e202504513. doi: 10.1002/anie.202504513 (PMC12184297; doi:10.1002/anie.202504513)
Supplement: Supplementary file 1 — Supporting Information [file ANIE-64-e202504513-s001.pdf]

Supporting Information for:

Influence of Ligand Design and Non-Covalent Interactions on the Isoselective Ring-Opening Polymerization of *rac*-BBL using Salan and Salalen Rare-Earth Metal Catalysts

Stefanie Hörl[a], Ion Chiorescu[b], Jonas Futter[a], Jonas Bruckmoser[a] and Bernhard Rieger[a*]

[a]WACKER-Chair of Macromolecular Chemistry, Catalysis Research Center, Department of Chemistry, Technical University of Munich
Lichtenbergstraße 4, 85748 Garching bei München, Germany.

[b]Theoretische Chemie, School of Natural Sciences, Technische Universität München, 85748 Garching, Germany

[*] Corresponding Author; email: rieger@tum.de

Table of Contents

1 Experimental Section 2
1.1 Materials and Methods 2
1.2 Pro-ligand Synthesis 3
1.3 Yttrium-Salalen Complex 10
2 Polymerization Data 16
2.1 General Polymerization Procedure 16
2.2 Polymer Characterization Data 19
2.3 Determination of Tacticity 21
2.4 SEC Characterization of ROP Polymers 24
3 Additional Characterization Data 26
3.1 Characterization of the Catalyst YL5 26
3.2 Kinetic Parameters 30
4 Computational Details 32
5 Cartesian coordinates of YL5 isomers 35
6 References 45

1 Experimental Section

1.1 Materials and Methods

All manipulations containing air- and/or moisture sensitive compounds were carried out under argon atmosphere using standard Schlenk or glovebox techniques. Glassware was flame-dried under vacuum prior to use. Unless otherwise stated, all chemicals were purchased from Sigma-Aldrich, TCI Chemicals or ABCR and used as received. Solvents were obtained from an MBraun MB-SPS 800 solvent purification system and stored over 3 Å molecular sieves prior to use. Deuterated chloroform ($CDCl_3$), benzene (C_6D_6), toluene (C_7D_8) and tetrahydrofuran (C_4D_8O) were obtained from Sigma-Aldrich and dried over 3 Å molecular sieves.

Nuclear magnetic resonance (NMR) spectra were recorded on a Bruker AV-III-500 spectrometer equipped with a QNP-Cryoprobe or AV-III-400 spectrometers at ambient temperature (298 K). ^{1}H and $^{13}C\{^{1}H\}$ NMR spectroscopic chemical shifts δ are reported in ppm relative to tetramethylsilane and were referenced internally to the relevant residual solvent resonances. The following abbreviations are used: br, broad; s, singlet; d, doublet; t, triplet; p, pentet; m, multiplet; AB, AB system.

Size exclusion chromatography (SEC) was performed on a PL-GPC50 plus from Polymer Laboratories with $CHCl_3$ (with amylene as stabilizing agent) as eluent at 40 °C. Size separation was done using two PolarGel Mixed-C columns by Agilent Technologies with a polymer sample concentration of 1.5-2.0 mg/L. Weight-average molecular weight (M_w), number-average molecular weight (M_n) and polydispersity indices ($Đ = M_w/M_n$) were determined *via* single RI-detection and reported against polystyrene standards.

Gas-chromatography mass spectrometry (GC-FID) measurements were performed on an Agilent GC-7890B equipped with a MSD 59771 mass detector, a 7693 automatic liquid sampler and a G4513A autoinjector. Sample separation is done using a HP-5MS UI column (30 m length, 0.25 mm diameter, 0.25 µm film) in a temperature range of 60-300 °C followed by mass spectrometry using full scan method in a mass range of 40-500 au. Samples are prepared by dissolving 1 mg/mL in HPLC grade dichloromethane prior to measurement.

Elemental Analysis
Elemental analysis was measured with EURO EA instrument from HEKAtech at the Laboratory for Microanalysis, Catalysis Research Center, Technical University of Munich.

LIFDI-MS
The mass spectra were taken using a Linden CMS LIFDI as ionization source and a ThermoFischer Scientific Exactive Plus Orbitrap as a detector. The sample application was performed via a fumed silica capillary from a glovebox under an argon atmosphere to enable the measurement of highly air-sensitive compounds.

Attenuated total reflection infrared spectroscopy (ATR-IR)
Spectra were recorded on a Bruker Vertex 70v ATR-IR spectrometer at room temperature under argon as inert gas.

1.2 Pro-ligand Synthesis

Salan [ONNO]^{H2} and salalen [ONNO]^{H} were obtained by the condensation reaction of the respective diamine and salicylaldehyde. 4-*t*Bu-2-(1,1-Diphenylethyl)salicylaldehyde and 4-*t*Bu-2-trityl-salicylaldehyde were synthesized according to literature procedures.[1-4]

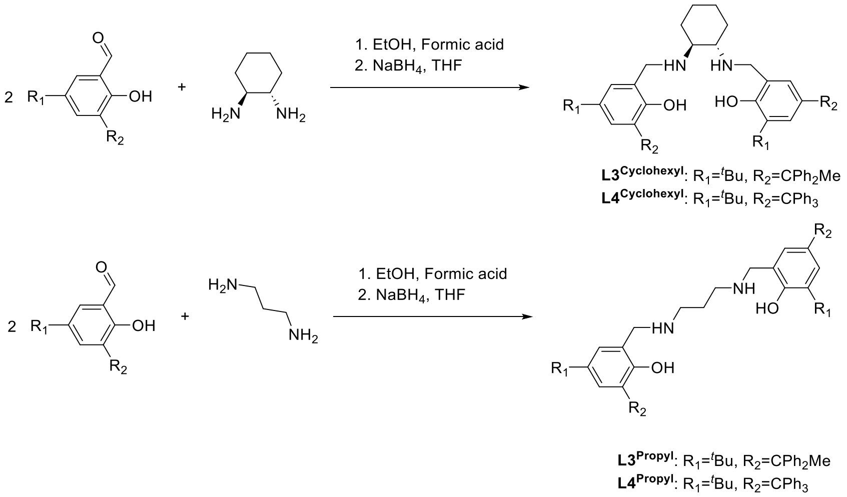

General procedure of salan pro-ligands L3-L4$^{Cyclohexyl / Propyl}$

The synthesis of salan pro-ligands **L3-L4$^{Cyclohexyl / Propyl}$** followed a similar literature known procedure[3, 5] and therefore the synthesis is described as a general procedure. For synthesis **L3-L4^{Cyclohexyl}**, the racemic diamine is used.

The respective aldehyde (19.5 mmol, 2.0 eq.) was dissolved in EtOH (100 mL) at 50 °C and subsequently, diamine (9.8 mmol, 1.0 eq.) and formic acid (2.7 mmol, 0.3 eq.) were added. The reaction mixture was refluxed for 6 h and cooled to room temperature. The resulting yellow precipitate was directly dissolved by addition of THF (100 mL), and the reaction mixture was cooled to 0°C. $NaBH_4$ (126.9 mmol, 13.0 eq.) was added portion wise and stirred for 1h at this temperature. Subsequently, the reaction mixture was allowed to warm to room temperature and stirred for 12 h until the solution was completely colorless. The solvent was removed *in vacuo*, the residue dissolved in DCM (200 mL) and H_2O (200 mL) was added. The phases were separated, and the organic layer washed with water (2x75 mL) and brine (75 mL). The organic layer was dried over Na_2SO_4, the solvent removed under reduced pressure, and the residue further purified as described below.

L3^{cyclohexyl}

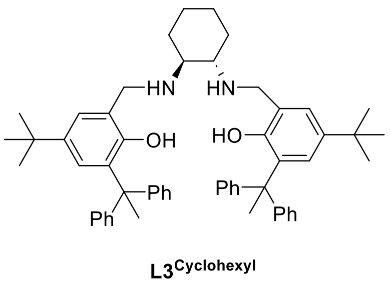

L3^{Cyclohexyl}

The residue was recrystallized from MeOH/DCM, resulting in pro-ligand **(L3^{Cyclohexyl})** (80%) as colorless solid.

^{1}H NMR (400 MHz, $CDCl_3$, 300 K): δ (ppm) = 7.30-7.12 (m, 20H, Ar*H*), 6.84 (d, $^4J_{H,H}$ = 2.4 Hz, 2H, Ar*H*), 6.59 (d, $^4J_{H,H}$ = 2.4 Hz, 2H, Ar*H*), 3.81 (d, $^3J_{H,H}$ = 13.8 Hz, 2H, HN-CH_2), 3.70 (d, $^3J_{H,H}$ = 14.2 Hz, 2H, HN-CH_2), 2.28 (s, 6H, Me), 2.15-2.01 (m, 4H, Cy*H*), 1.71-1.60 (m, 2H, Cy*H*), 1.64-1.60 (2H, Cy*H*),1.11 (s, 18H,^{t}Bu), 1.11 (m, 2H, Cy*H*) 1.01-0.89 (m, 2H, Cy*H*).

^{13}C{^{1}H} NMR (101 MHz, $CDCl_3$, 300 K): δ (ppm) = 153.9, 148.9, 148.8, 140.1, 134.9, 128.5, 128.4 127.6, 127.5, 127.0. 125.6, 125.5, 123.7, 122.2, 59.2, 52.0, 50.1, 34.0, 31.5, 30.6, 27.70, 24.4

Elemental Analysis: Anal. Calc. for $C_{56}H_{66}N_2O_2$: C, 84.17; H, 8.32; N, 3.51. Found: C, 83.52; H, 8.58, N, 3.40%.

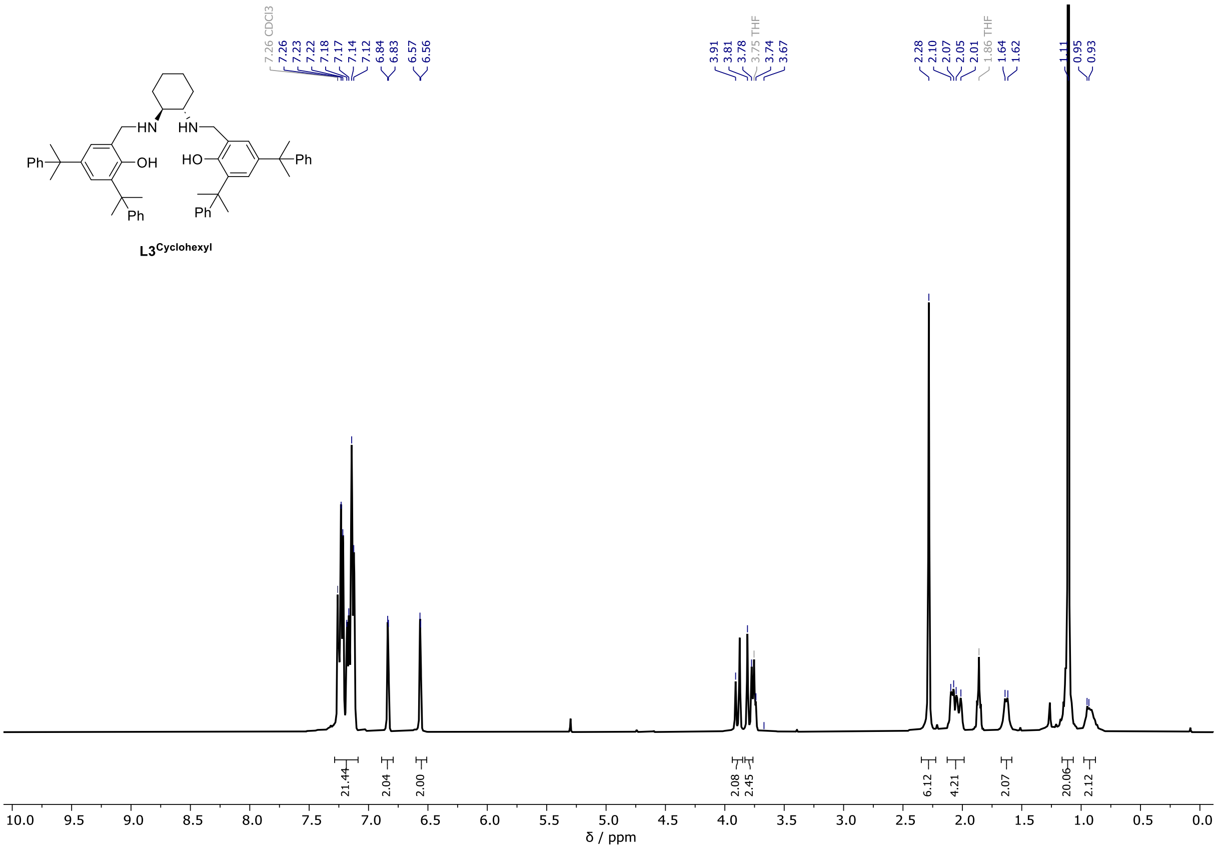

Figure S1: ^{1}HNMR spectrum (400 MHz, $CDCl_3$, 300 K) of pro-ligand **L3^{Cyclohexyl}**.

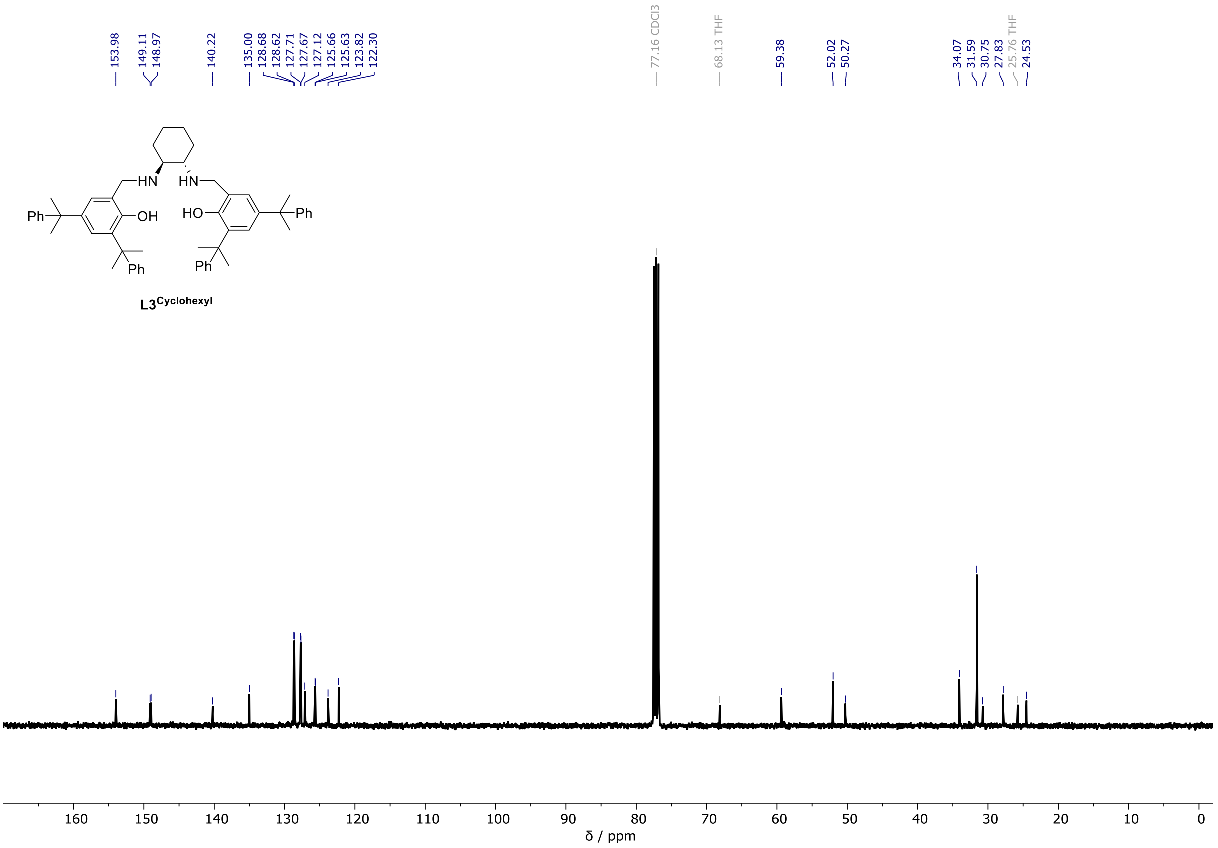

Figure S2: $^{13}C\{^{1}H\}$ NMR spectrum (101 MHz, $CDCl_3$, 300 K) of pro-ligand **L3^{Cyclohexyl}**.

L4^{cyclohexyl}

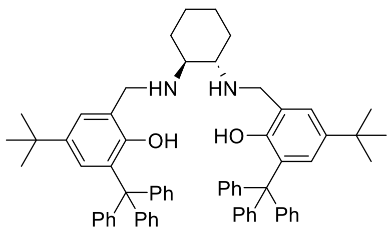

L4^{Cyclohexyl}

The residue was washed with MeOH, resulting in pro-ligand **(L4^{Cyclohexyl})** (74%) as colorless solid.

^{1}H NMR (400 MHz, $CDCl_3$, 300 K): δ (ppm) = 10.30 (s br, 2H, OH), 7.21-7.08 (m, 30 H, Ar*H*), 7.05 (d, $^{4}J_{H,H}$ = 2.6 Hz, 2H, Ar*H*), 6.83 (d, $^{4}J_{H,H}$ = 2.6 Hz, 2H, Ar*H*), 3.79 (d, $^{3}J_{H,H}$ = 13.8 Hz, 2H, HN-C*H*$_2$), 3.66 (d, $^{3}J_{H,H}$ = 13.8 Hz, 2H, HN-C*H*$_2$), 1.78 (d, $^{3}J_{H,H}$ = 12.7, 2H, Cy*H*), 1.51-1.48 (m, 4H, Cy*H*), 1.13 (s, 18 H, ^{t}Bu), 0.86-0.82 (m, 2H, Cy*H*), 0.55-0.53 (m, 2H, CyH).

$^{13}C\{^{1}H\}$ NMR (101 MHz, $CDCl_3$, 300 K): δ (ppm) = 153.9, 146.2, 139.9, 133.2, 131.2, 127.3, 126.9, 125.3, 124.1, 121.7, 63.37, 57.8, 49.3, 34.1, 31.5, 29.8, 24.5.

Elemental Analysis: Anal. Calc. for $C_{66}H_{70}N_2O_2$: C, 85.86; H, 7.64; N, 3.03. Found: C, 85.74; H, 7.80, N, 3.16%.

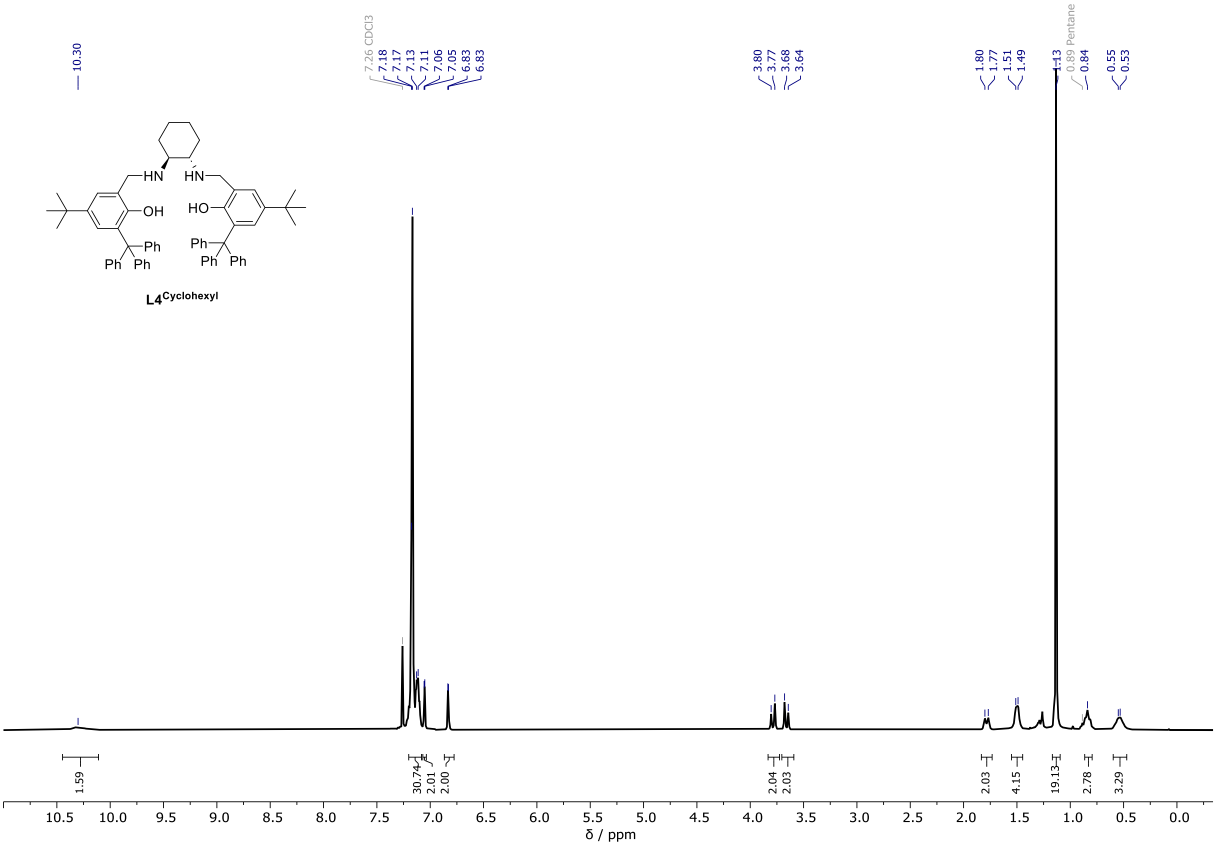

Figure S3: ^{1}H NMR spectrum (400 MHz, $CDCl_3$, 300 K) of pro-ligand **L4^{Cyclohexyl}**.

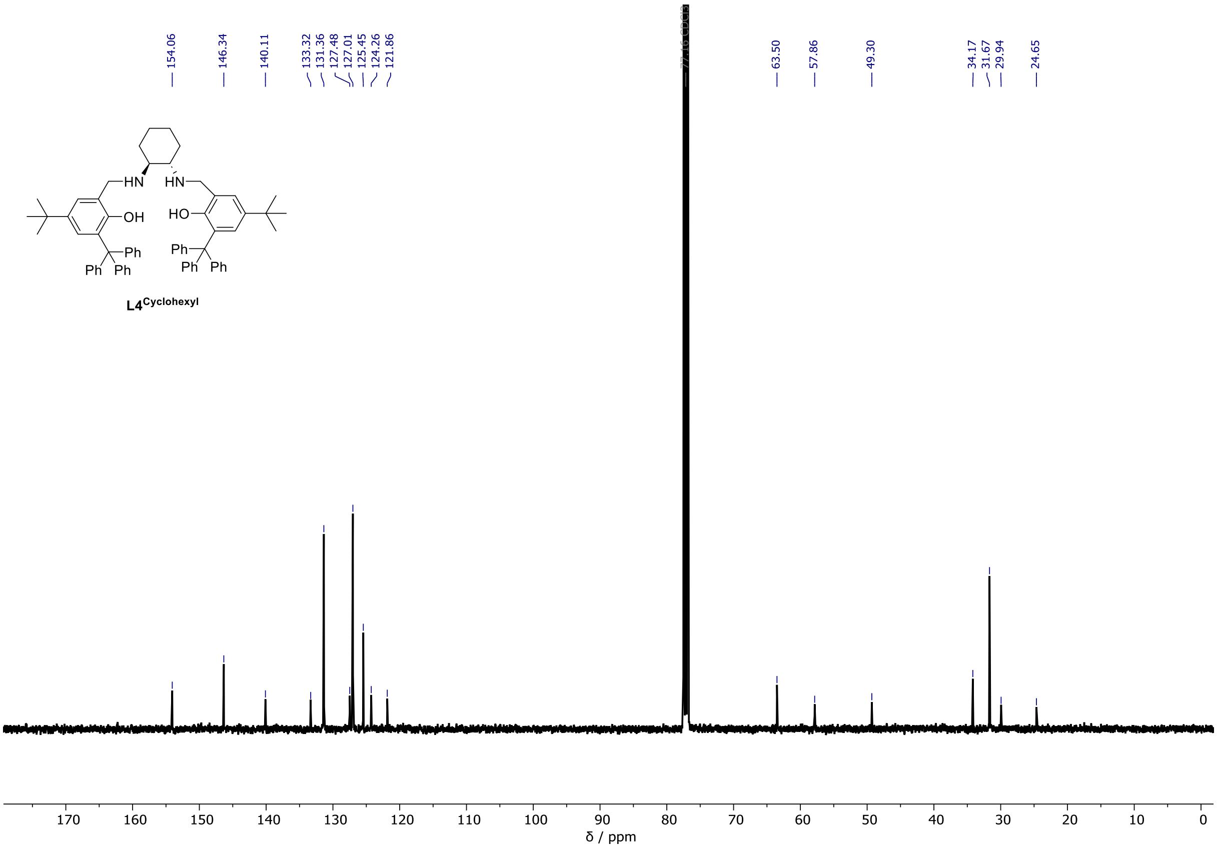

Figure S4: $^{13}C\{^1H\}$ NMR spectrum (101 MHz, $CDCl_3$, 300 K) of pro-ligand **L4^{Cyclohexyl}**.

L3^{Propyl}

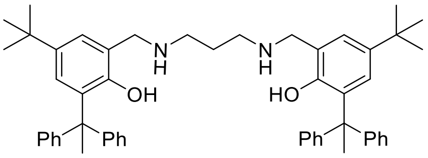

L3^{Propyl}

The residue was washed with MeOH, resulting in pro-ligand **(L3**^{Propyl}**)** (78%) as colorless solid.

^{1}H NMR (400 MHz, $CDCl_3$, 300 K): δ (ppm) = 7.30-7.15 (m, 20 H, Ar*H*), 6.90 (d, $^{4}J_{H,H}$ = 2.6 Hz, 2H, Ar*H*), 6.54 (d, $^{4}J_{H,H}$ = 2.6 Hz, 2H, Ar*H*), 3.90 (s, 4H, HN-CH_2), 2.59 (t, $^{3}J_{H,H}$ = 6.9 Hz, 4H, CH_2-CH_2-NH), 2.33 (s, 6H, Me), 1.59 (p, $^{3}J_{H,H}$ = 6.9 Hz, 2H, CH_2-CH_2-NH), 1.12 (s, 18H, ^{t}Bu).

^{13}C{^{1}H} NMR (101 MHz, $CDCl_3$, 300 K): δ (ppm) = 154.1, 149.0, 140.2, 134.9, 128.6, 127.7, 127.4, 125.7, 123.8, 121.9, 53.4, 52.0, 46.4, 34.0, 31.5, 29.6, 27.6.

Elemental Analysis: Anal. Calc. for $C_{53}H_{62}N_2O_2$: C, 83.86; H, 8.23; N, 3.69. Found: C, 83.84; H, 8.33, N, 3.87%.

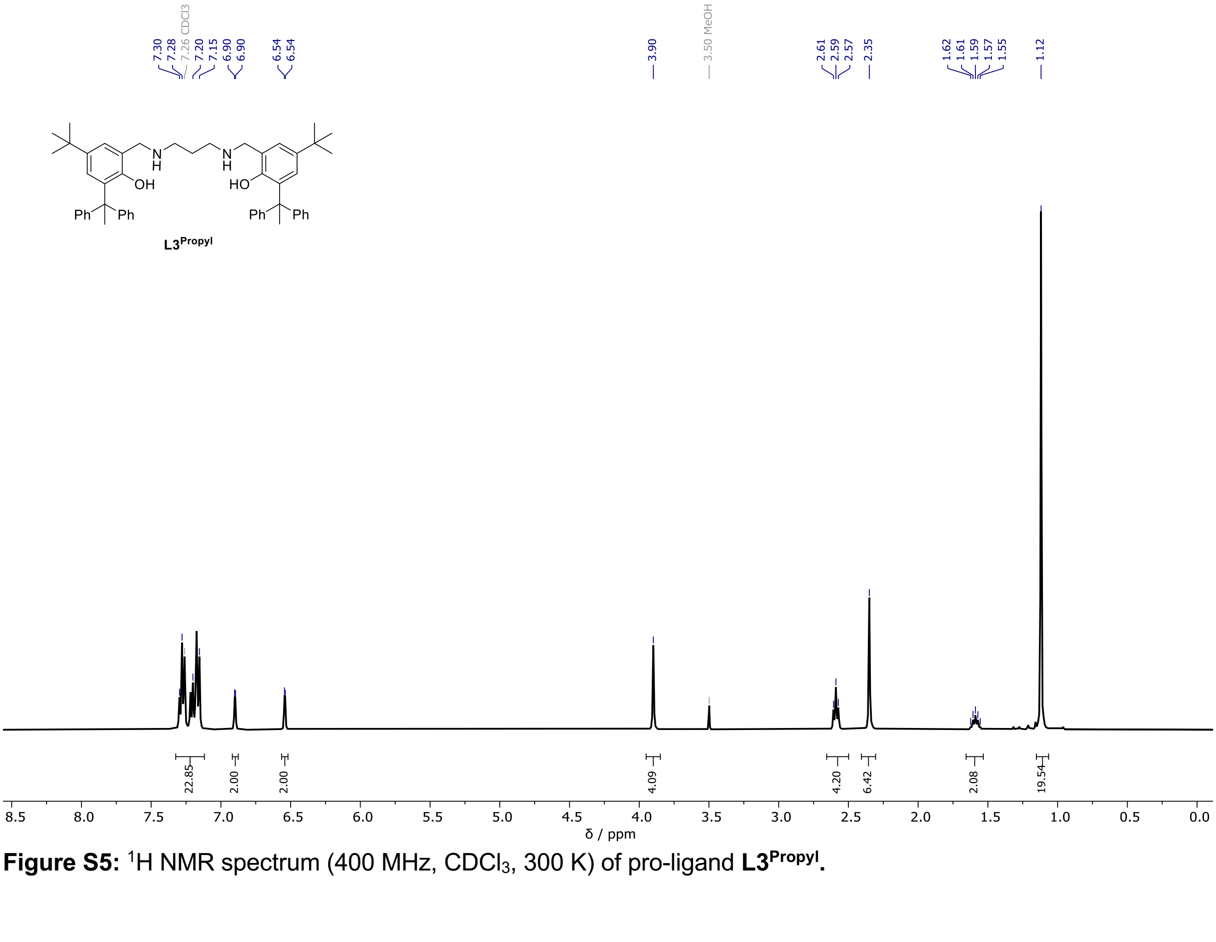

Figure S5: ^{1}H NMR spectrum (400 MHz, $CDCl_3$, 300 K) of pro-ligand **L3**^{Propyl}.

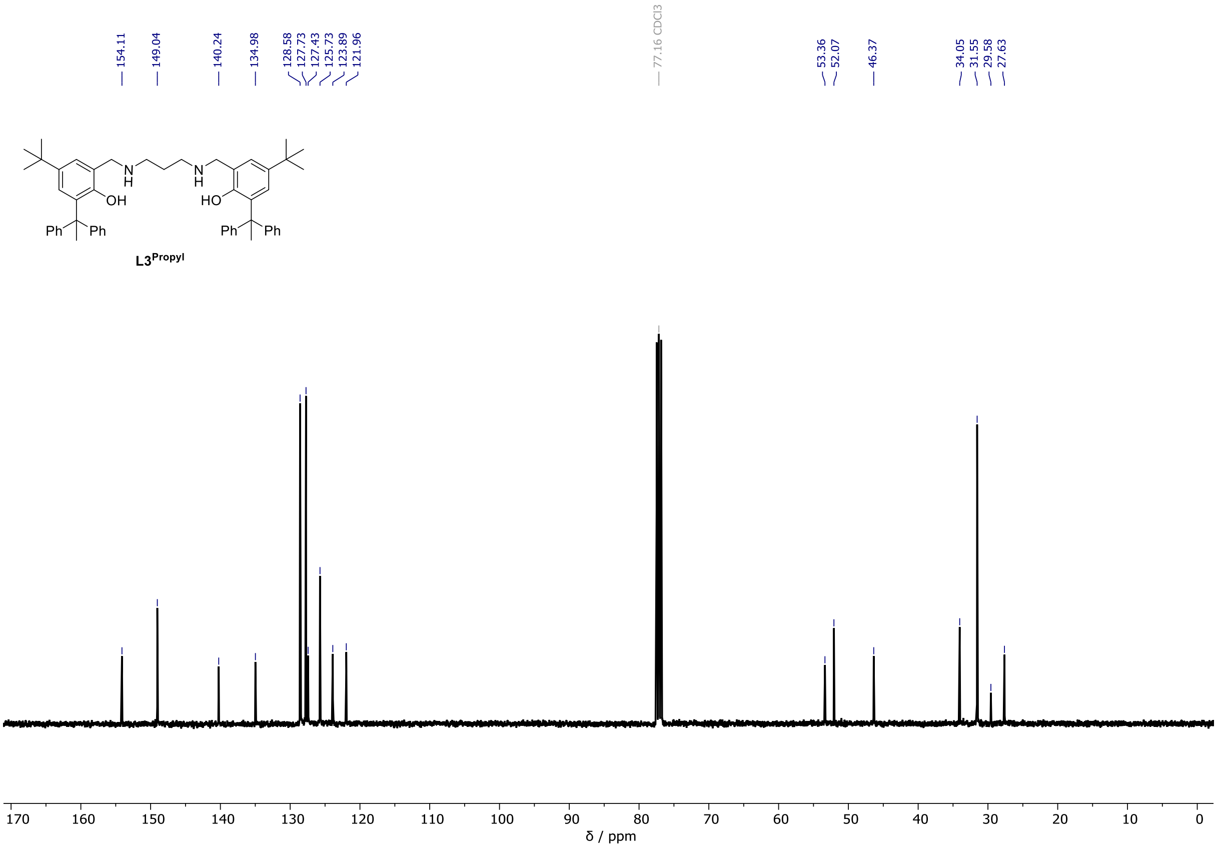

Figure S6: $^{13}C\{^1H\}$ NMR spectrum (101 MHz, $CDCl_3$, 300 K) of pro-ligand **L3^{Propyl}**.

L4^{Propyl}

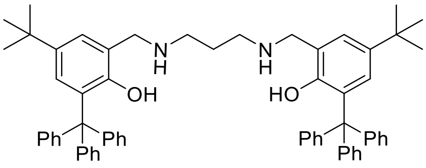

L4^{Propyl}

The residue was recrystallized in MeOH/DCM, resulting in pro-ligand **(L4^{Propyl})** (63%) as colorless solid.

^{1}H NMR (400 MHz, $CDCl_3$, 300 K): δ (ppm) = 7.23-7.08 (m, 32H, Ar*H*), 6.91 (d, $^4J_{H,H}$ = 2.4 Hz, 2H, Ar*H*), 3.82 (s, 4H, HN-CH_2), 2.27 (t, $^3J_{H,H}$ = 7.0 Hz, 4H, CH_2-CH_2-NH), 1.59 (p, $^3J_{H,H}$ = 6.9 Hz, 2H, CH_2-CH_2-NH), 1.25-1.18 (m, 2H, CH_2-CH_2-NH)1.12 (s, 18H, ^{t}Bu).

$^{13}C\{^1H\}$ NMR (101 MHz, $CDCl_3$, 300 K): δ (ppm) = 154.2, 146.3, 140.3, 133.5, 131.5, 127.8, 127.0, 125.5, 124.3, 121.9, 63.5, 53.2, 46.2, 34.2, 31.7, 29.5.

Elemental Analysis: Anal. Calc. for $C_{63}H_{66}N_2O_2$: C, 85.67; H, 7.53; N, 3.17. Found: C, 85.58; H, 7.58, N, 3.34%.

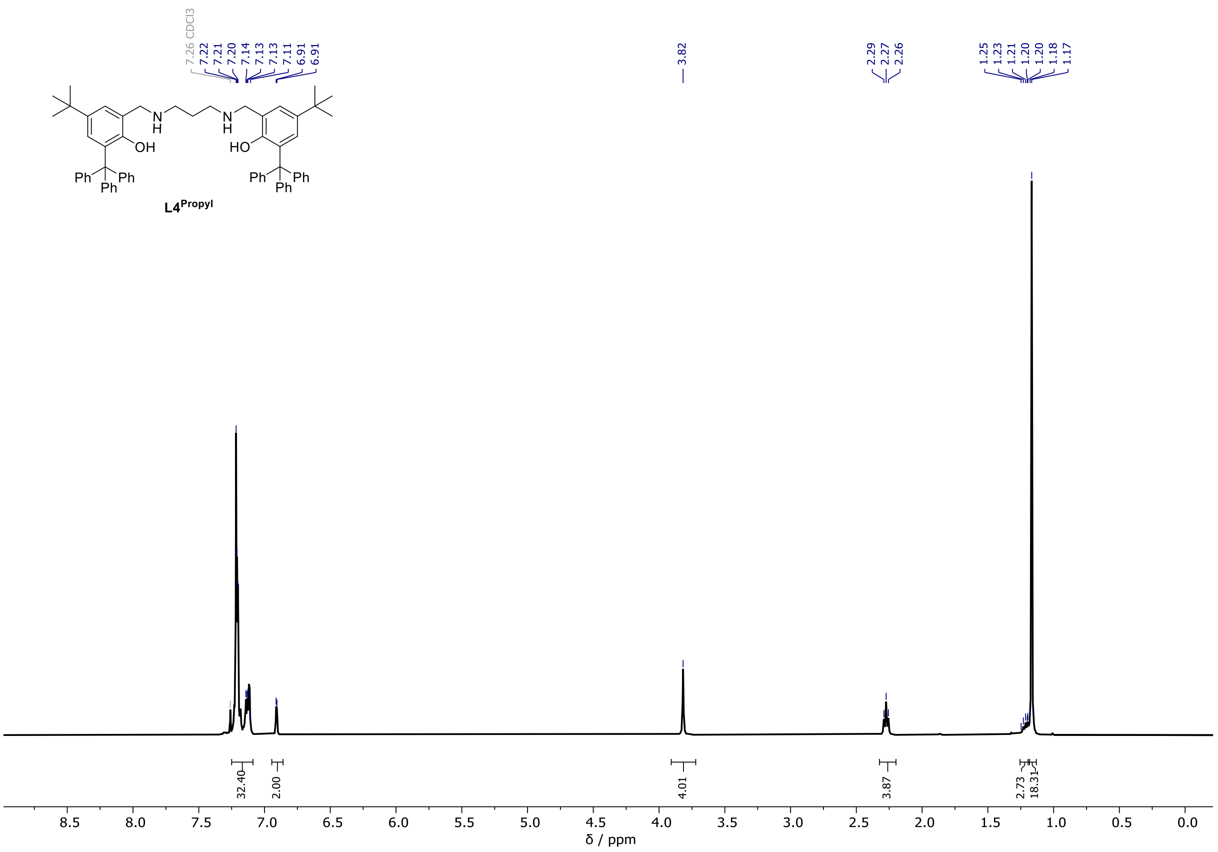

Figure S7: ^{1}H NMR spectrum (400 MHz, $CDCl_3$, 300 K) of pro-ligand **L4^{Propyl}**.

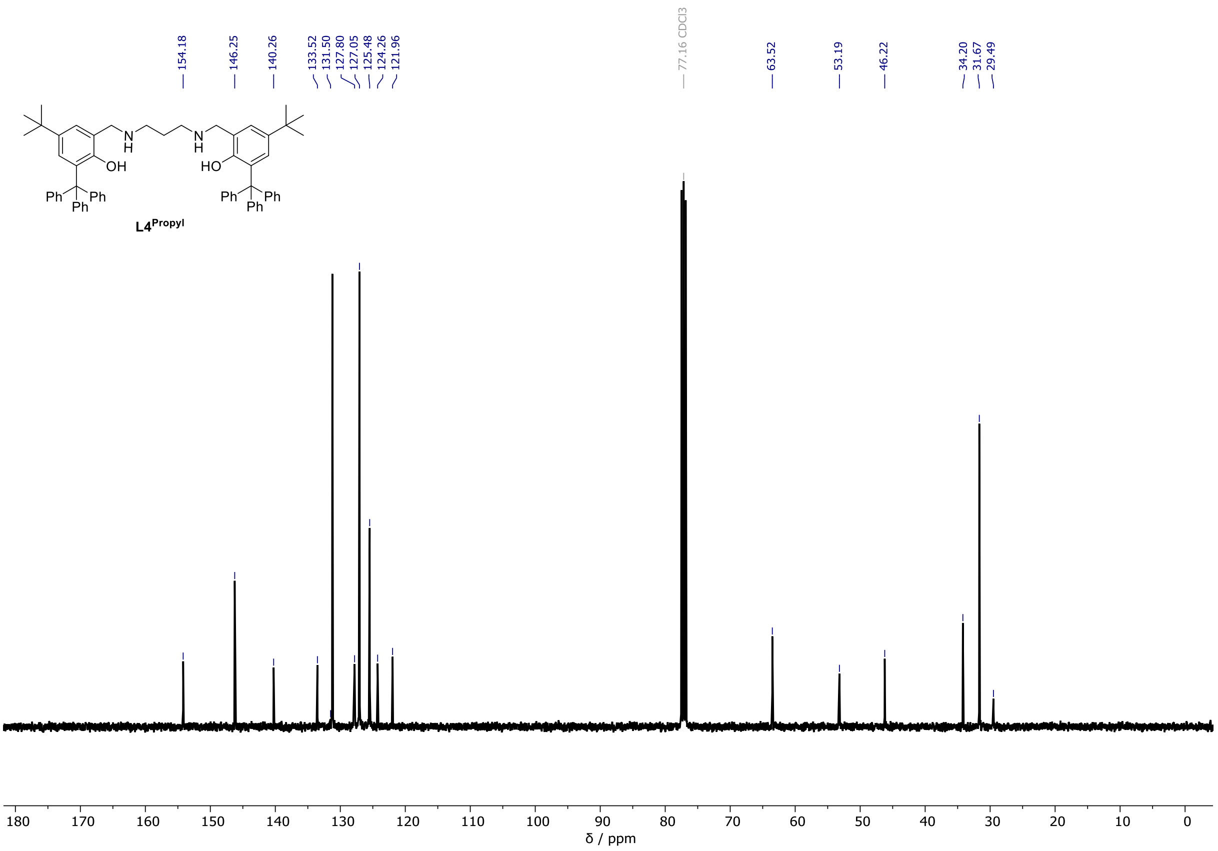

Figure S8: $^{13}C\{^{1}H\}$ NMR spectrum (101 MHz, $CDCl_3$, 300 K) of pro-ligand **L4^{Propyl}**.

1.3 Yttrium-Salalen Complex

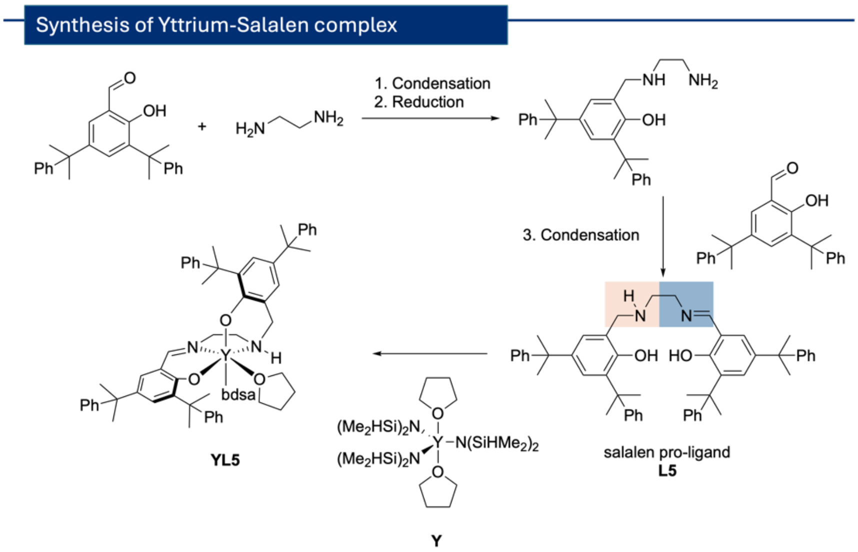

Scheme S1. Reaction pathway for the synthesis of **YL5.**

Dicumylethtylendiamine Precursor L5'

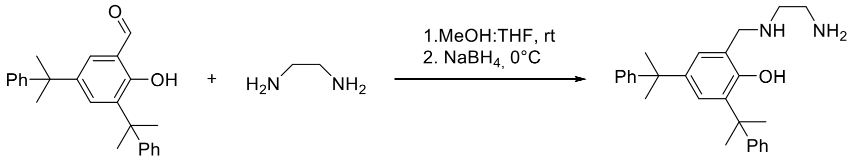

L5'

The salalen ligand [ONNO]^{H} was prepared following an adopted literature procedure.[6] Dicumylsalicylaldehyde (5.00 g, 14.0 mmol, 1 eq.) was dissolved in MeOH:THF (150 mL; 5:1), and ethylendiamine (0.84 g, 14.0 mmol, 1 eq.) diluted in MeOH (60 mL) was added dropwise to the reaction mixture and was stirred overnight at room temperature. Subsequently, THF was added (50 mL), and $NaBH_4$ (0.57 g, 15.0 mmol, 2.7 eq.) was added portion wise at 0°C. The reaction mixture was stirred for 2h, was then led to rise to room temperature, and subsequently stirred for another 2h. The solvent was removed *in vacuo* and water (100 mL) and DCM (100 mL) were added. The phases were separated, and the organic phase was washed with water (2 x 100mL), brine (1 x 100 mL) and dried over $MgSO_4$. The solution was removed *in vacuo,* and the side product was recrystallized in EtOH. The solvent of the supernatant was removed *in vacuo* resulting in the curde product **L5'** (3.4 g, 8.4 mmol, 60%) as colorless oil.

^{1}H NMR (400 MHz, $CDCl_3$, 300 K): *δ* (ppm) = 7.32-7.10 (m, 11H, Ar*H*), 6.72 (d, $^{4}J_{H,H}$ = 2.4 Hz, 2H, Ar*H*), 3.82 (s, 2H, HN-CH_2), 2.67 (td, $^{3}J_{H,H}$ = 6.4, 4.9 Hz, 2H, CH_2-NH_2), 2.54 (td, $^{3}J_{H,H}$ = 6.4, 4.9 Hz, 2H, CH_2-CH_2-NH_2), 1.67 (s, 6H, Me), 1.64 (s, 6H, Me).

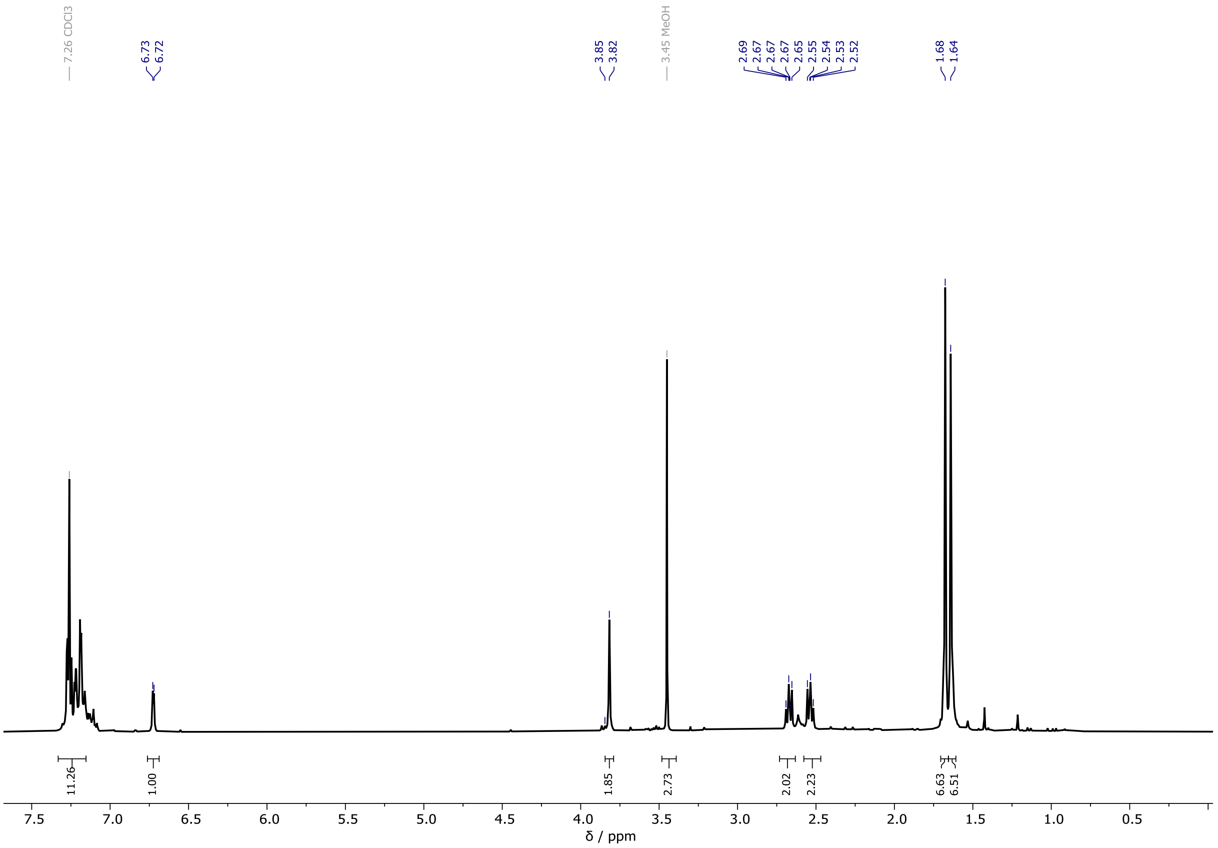

Figure S9: ^{1}H NMR spectrum (400 MHz, $CDCl_3$, 300 K) of precursor **L5'**.

Dicumylethyl-salalen Propligand L5

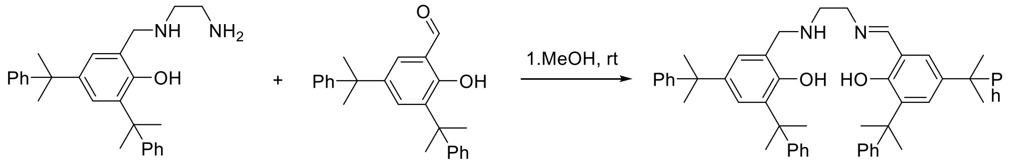

L5' **L5**

Dicumylsalicylaldehyde (3.0 g, 8.4 mmol, 1 eq.) was dissolved in MeOH (60 mL) and **L5'** (3.4 g, 8.4 mmol, 1 eq.) was added at room temperature. The reaction mixture was stirred for 24 h and the resulting suspension was filtered and washed with pentane (30 mL). The resulting crude product was recrystallized in MeOH:THF resulting in the $[ONNO]^{H}$ salalen pro-ligand **L5** (4.4 g, 5.9 mmol, 70 %) as slightly yellow solid.

^{1}H NMR (400 MHz, $CDCl_3$, 300 K): δ (ppm) = 13.01 (s, 1H, OH), 10.41 (s br, 1H, OH), 8.12 (s, 1H, N=C*H*), 7.35 (d, $^{4}J_{H,H}$ = 2.4 Hz, 1H, Ar*H*), 7.32-7.07 (m, 21H, Ar*H*), 6.98 (d, $^{4}J_{H,H}$ = 2.4 Hz, 2H, Ar*H*), 6.71 (d, $^{4}J_{H,H}$ = 2.5 Hz, 2H, CH_2-Ar*H*), 3.78 (s, 2 H, HN-CH_2-Ar), 3.41 (t, $^{3}J_{H,H}$ = 5.3 Hz, 2H, CH_2-CH_2=N), 2.72 (t, $^{3}J_{H,H}$ = 5.8 Hz, 2H, CH_2-CH_2-NH), 1.71 (s, 6H, Me), 1.67 (s, 6H, Me), 1.65 (s, 6H, Me), 1.63 (s, 6H, Me).

^{13}C{^{1}H} NMR (101 MHz, $CDCl_3$, 300 K): δ (ppm) = 167.4, 157.6, 154.0, 151.5, 151.4, 150.8, 150.7, 140.2, 140.0, 136.2, 135.5, 129.3, 128.2, 128.1, 128.0, 128.0, 127.8, 126.9, 126.9, 125.8, 125.7, 125.7, 125.6, 125.6, 125.2, 125.2, 125.1, 121.9, 118.0, 59.0, 53.6, 52.7, 48.0, 42.6, 42.5, 42.2, 42.2, 31.2, 31.0, 29.7, 29.6.

Elemental Analysis: Anal. Calc. for $C_{52}H_{58}N_2O_2$: C, 84.06; H, 7.87; N, 3.77. Found: C, 84.07; H, 8.12, N, 3.76%.

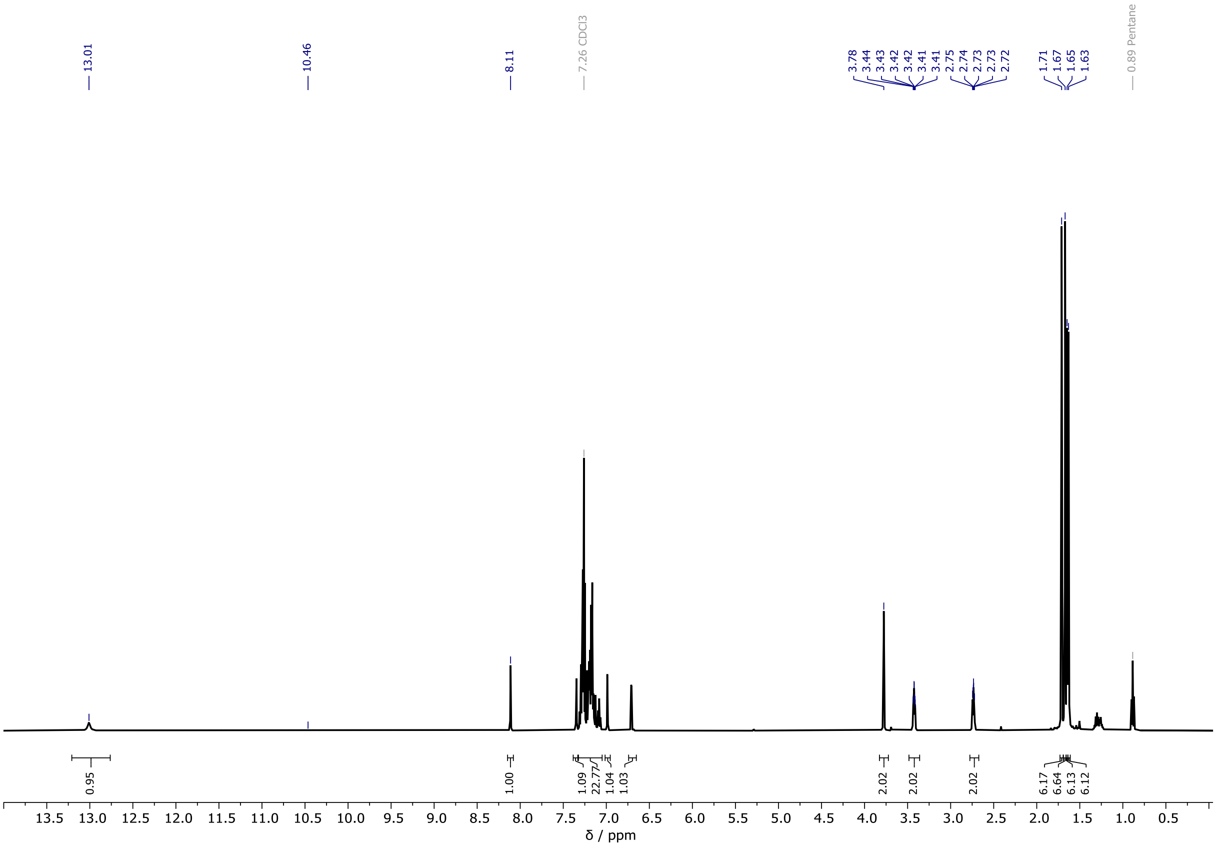

Figure S10: ^{1}H NMR spectrum (400M Hz, $CDCl_3$, 300 K) of pro-ligand **L5.**

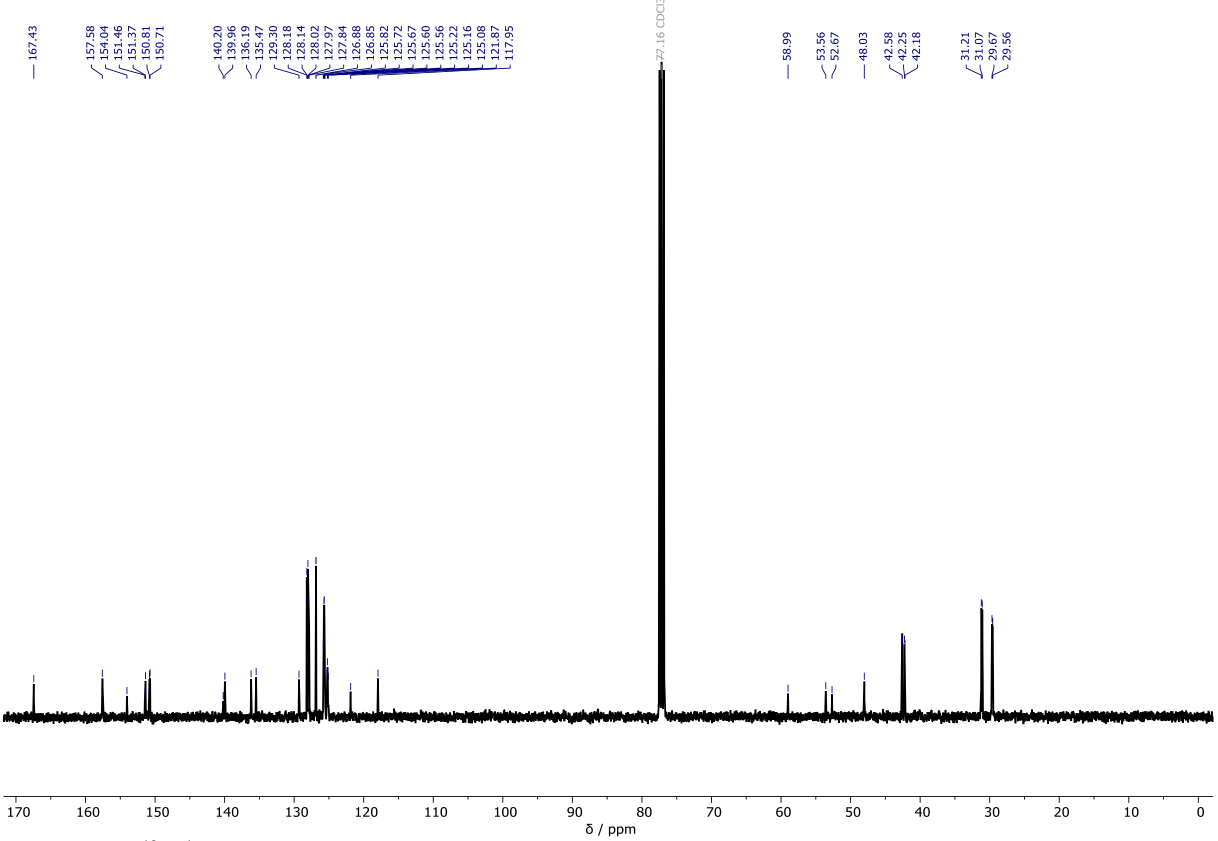

Figure S11: $^{13}C\{^1H\}$ NMR spectrum (101 MHz, $CDCl_3$, 300 K) of pro-ligand **L5**.

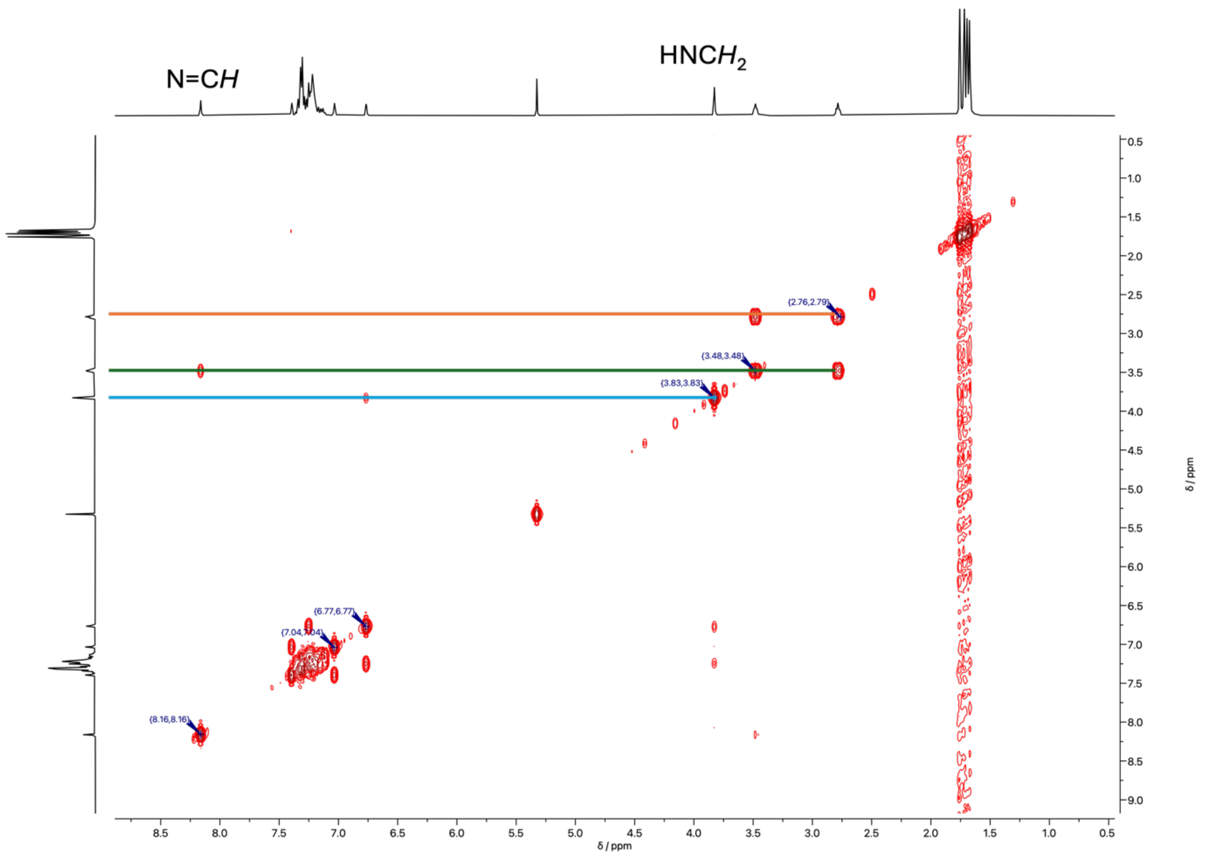

Figure S12: ^{1}H-COSY NMR spectrum of pro-ligand **L5**.

Deuterated Dicumylethyl-salalen Propligand L5^D

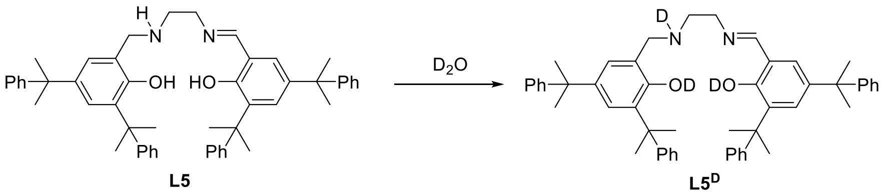

Deuterated dicumylethylsalalen was synthesized according to a literature known procedure.[7] Dicumylethylsalalen (0.50 g, 0.67 mmol, 1.00 eq.) was dissolved in $CDCl_3$ (3 mL) and overlaid with D_2O (6 mL). After 3h, the D_2O was exchanged twice over 24h, and subsequently, solvent was removed *in vacuo*, yielding the product **L5^D** (0.45 g, 0.60 mmol, 90%) as a yellow powder. The pro-ligand was stored in the glove box to ensure no exchange of deuterium with protons appears with the moisture in the air.

^{1}H NMR (400 MHz, $CDCl_3$, 300 K): δ (ppm) = 13.03 (s, 1D, -OD), 10.52 (bs, 1D, -OD), 8.10 (s, 1H, N=CH-Ar), 7.35 (d, $^4J_{H,H}$= 2.4, 1H, Ar*H*), 7.31-7.06 (m, 21H, Ar*H*), 6.98 (d, $^4J_{H,H}$= 2.4, 1H, Ar*H*), 6.71 (d, $^4J_{H,H}$= 2.5, 1H, Ar*H*), 3.76 (s, 2H, , DN-C*H*$_2$-Ar), 3.41 (t, $^3J_{H,H}$= 5.3, 2H, CH_2-C*H*$_2$-N=), 2.72 (t, $^3J_{H,H}$= 5.8, 2H, CH_2-C*H*$_2$-ND), 1.71 (s, 6H, Me), 1.67 (s, 6H, Me), 1.65 (s, 6H, Me), 1.63 (s, 6H, Me).

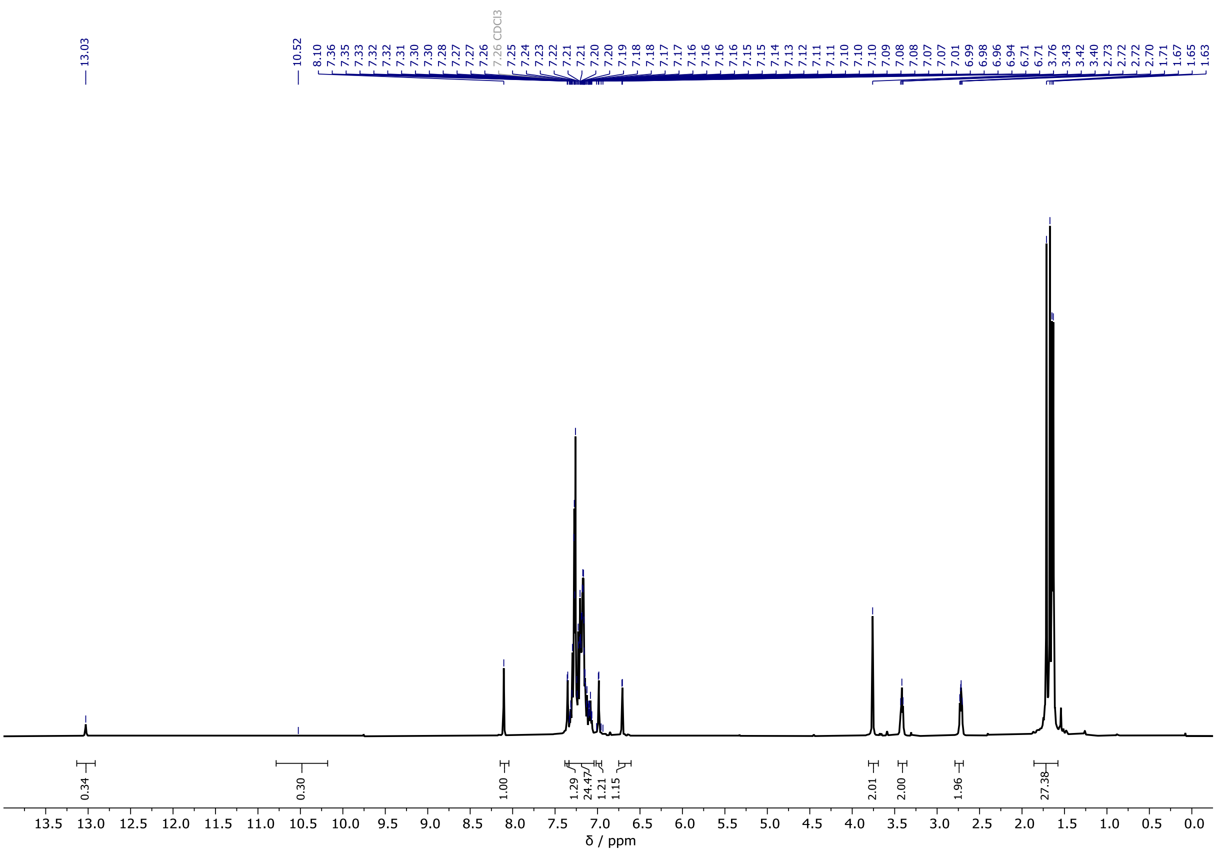

Figure S13: ^{1}H NMR spectrum (400 MHz, $CDCl_3$, 300 K) of pro-ligand **L5^D**.

Dicumylethyl-Salalen-Yttrium Complex YL5

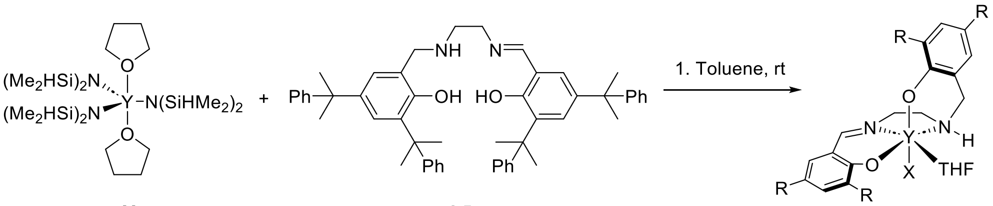

Y **L5**

YL5
X = N(SiHMe$_2$)$_2$
R = CPhMe$_2$

Y(bdsa)$_3$(THF)$_2$ (0.80 g, 1.27 mmol, 1 eq.) was dissolved in toluene (10 mL), and a solution of the pro-ligand **L5** (0.94 g, 1.27 mmol, 1 eq.) dissolved in toluene (10 mL) was added dropwise at room temperature. The reaction mixture was stirred for 22h, and after evaporation of the solvent, the crude product was washed with pentane (2 x 10 mL). The crude product was recrystallized in pentane yielding **YL5** (0.35 g, 0.34 mmol, 27%) as slightly yellow solid.

^{1}H NMR (400 MHz, $CDCl_3$, 300 K): δ (ppm) = 7.57 (d, $^4J_{H,H}$ = 2.6 Hz, 1H, ArH_{Imin}), 7.50 (d, $^4J_{H,H}$ = 2.5 Hz, 1H, ArH_{Amine}), 7.27 (s, 1H, C*H*=N), 7.48-6.80 (m, 21H, Ar$H_{Dicumyl}$ + ArH_{Imin}), 6.88 (d, 1H, $^4J_{H,H}$ = 2.5 Hz, ArH_{Amine}), 4.74 (p, $^4J_{H,H}$ = 3.0 Hz, 2H, Si*H*), 4.33 (d, $^3J_{H,H}$ = 12.4 Hz, 1H, Ar-CH_2^a-NH), 3.31 (t, $^3J_{H,H}$ = 11.3 Hz, 1H, =N-CH_2^a-CH$_2$), 3.20 (bs, 4H, THF), 3.07 (dd, $^3J_{H,H}$ = 12.7, 3.0 Hz, 1H, Ar-CH_2^a-NH), 2.48-2.41 (m, 1H, NH), 2.37 (d, $^3J_{H,H}$ = 13.3, 1H, =N-CH_2^a-CH$_2$), 2.12-2.08 (m, 2H, CH$_2$-CH_2^a-NH), 2.08 (s, 3H, Me), 1.99.1.89 (m, 1H, CH$_2$-CH_2^a-NH), 1.82 (s, 3H, Me), 1.79 (s, 3H, Me), 1.78 (s, 3H, Me), 1.69 (s, 3H, Me), 1.68 (s, 6H, Me), 1.58 (s, 3H, Me), 1.33-1.26 (bs, 4H, THF), 0.18 (dd, $^3J_{H,H}$ = 10.9, 3.0 Hz, 12H, Si-Me).

$^{13}C\{^{1}H\}$ NMR (101 MHz, $CDCl_3$, 300 K): *δ* (ppm) = 169.7, 164.1, 164.0, 160.6, 160.5, 153.2, 152.5, 151.7, 151.1, 137.9, 137.3, 136.1, 135.7, 135.6, 133.0, 132.8, 129.3, 128.6, 127.3, 127.2, 126.8, 126.5, 126.3, 125.9, 125.7, 125.6, 125.4, 124.6, 123.4, 122.1, 69.9, 61.3, 52.4, 46.2, 43.6, 42.8, 42.7, 42.5, 33.4, 33.1, 31.8, 31.7, 31.3, 31.1, 29.1, 27.8, 25.8, 21.4, 3.8, 3.7.

Elemental Analysis: Anal. Calc. for $C_{60}H_{78}N_3O_3Si_2Y$: C, 69.67; H, 7.60; N, 4.06. Found: C, 69.36; H, 7.35, N, 4.07%.

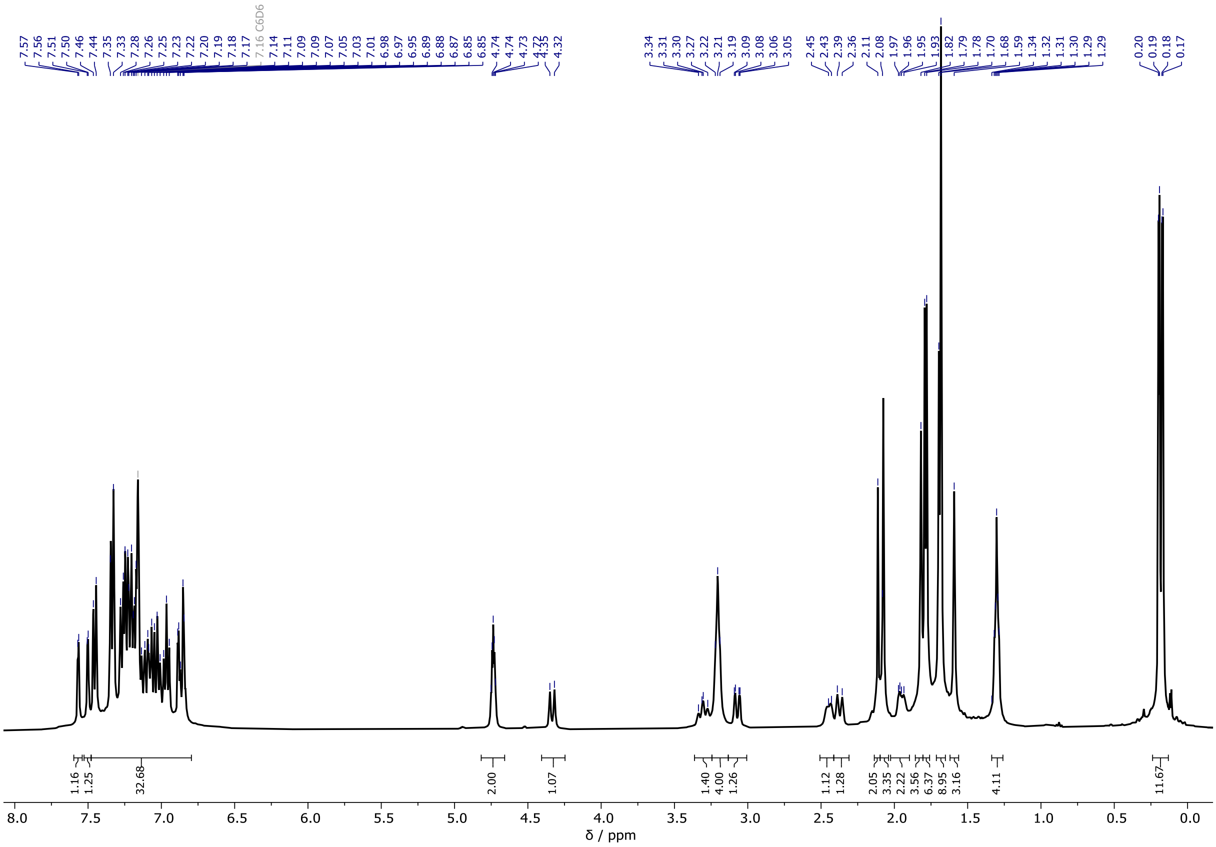

Figure S14: ^{1}H NMR spectrum (400 MHz, $CDCl_3$, 300 K) of catalyst **YL5.**

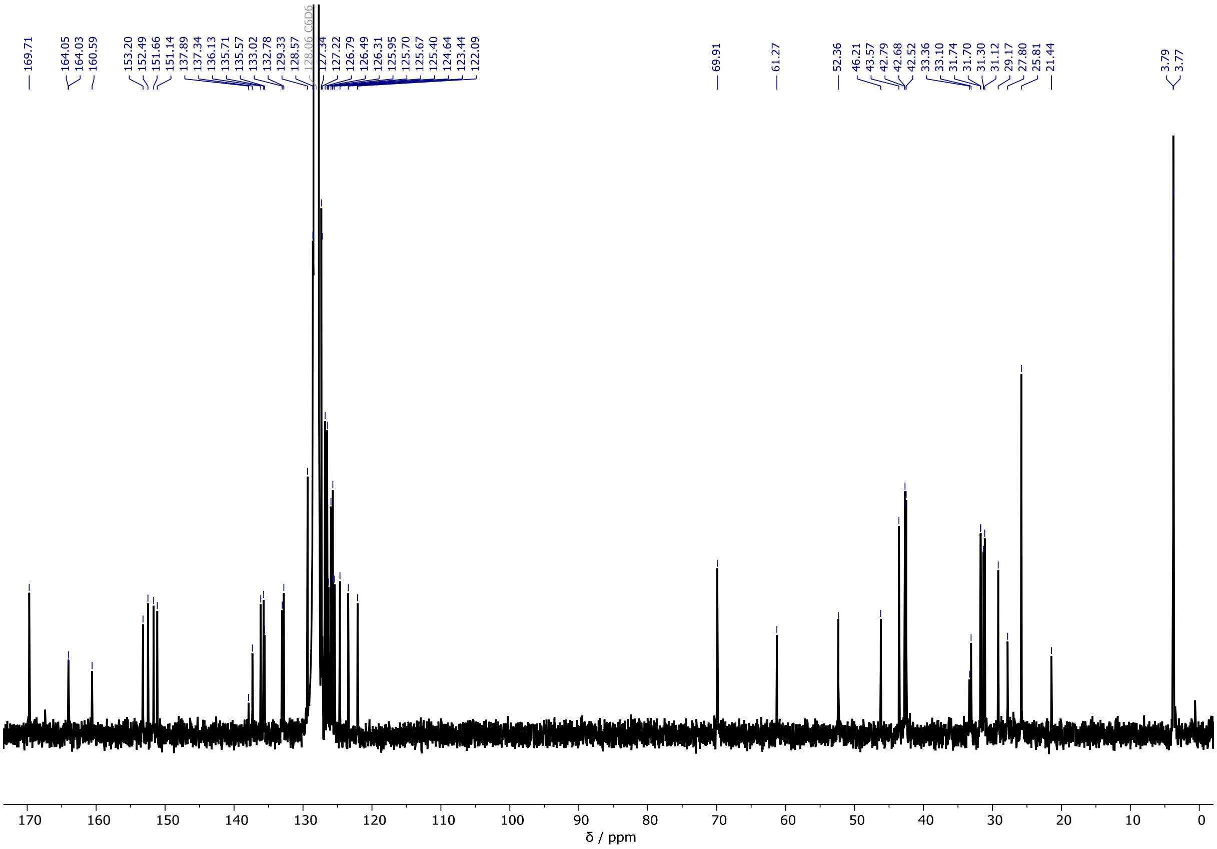

Figure S15: $^{13}C\{^{1}H\}$ NMR spectrum (101 MHz, $CDCl_3$, 300 K) of catalyst Y**L5**.

2 Polymerization Data

2.1 General Polymerization Procedure

Ring-opening polymerization using *in situ* generated catalysts

In a glove box, a 20 mL glass reactor was charged with a predetermined amount of $Y(bdsa)_3(THF)_2$ (1 eq.) and salan pro-ligand (1 eq., **L1 – L5**). The respective amount of toluene was added such that the overall monomer concentration after *rac*-BBL addition is 2.0 M. The reaction mixture was stirred for 1 h at room temperature and then, *rac*-BBL (equivalents as specified in the polymerization table) was added to this mixture. After stirring for a desired time period at room temperature, the polymerization was quenched by the addition of 0.5 mL of wet $CDCl_3$. An aliquot sample was taken for determination of conversion by ^{1}H NMR spectroscopy. The quenched mixture was then precipitated into 20 mL of diethyl ether/pentane (1/1), filtered, washed with diethyl ether/pentane (1/1) and dried in vacuo.

Ring-opening polymerization using isolated catalysts YL5

In a glove box, a 20 mL glass reactor was charged with a predetermined amount of catalyst (1 eq.) and the respective amount of toluene was added such that the overall monomer concentration after *rac*-BBL addition is 2.0 M. The reaction mixture was stirred for 15 min at the respective temperature and then, *rac*-BBL (equivalents as specified in the polymerization table) was added to this mixture. After stirring for a desired time period at room temperature, the polymerization was quenched by the addition of 0.5 mL of wet $CDCl_3$. An aliquot sample was taken for determination of conversion by ^1H NMR spectroscopy. The quenched mixture was then precipitated into 20 mL of diethyl ether/pentane (1/1), filtered, washed with diethyl ether/pentane (1/1) and dried in vacuo.

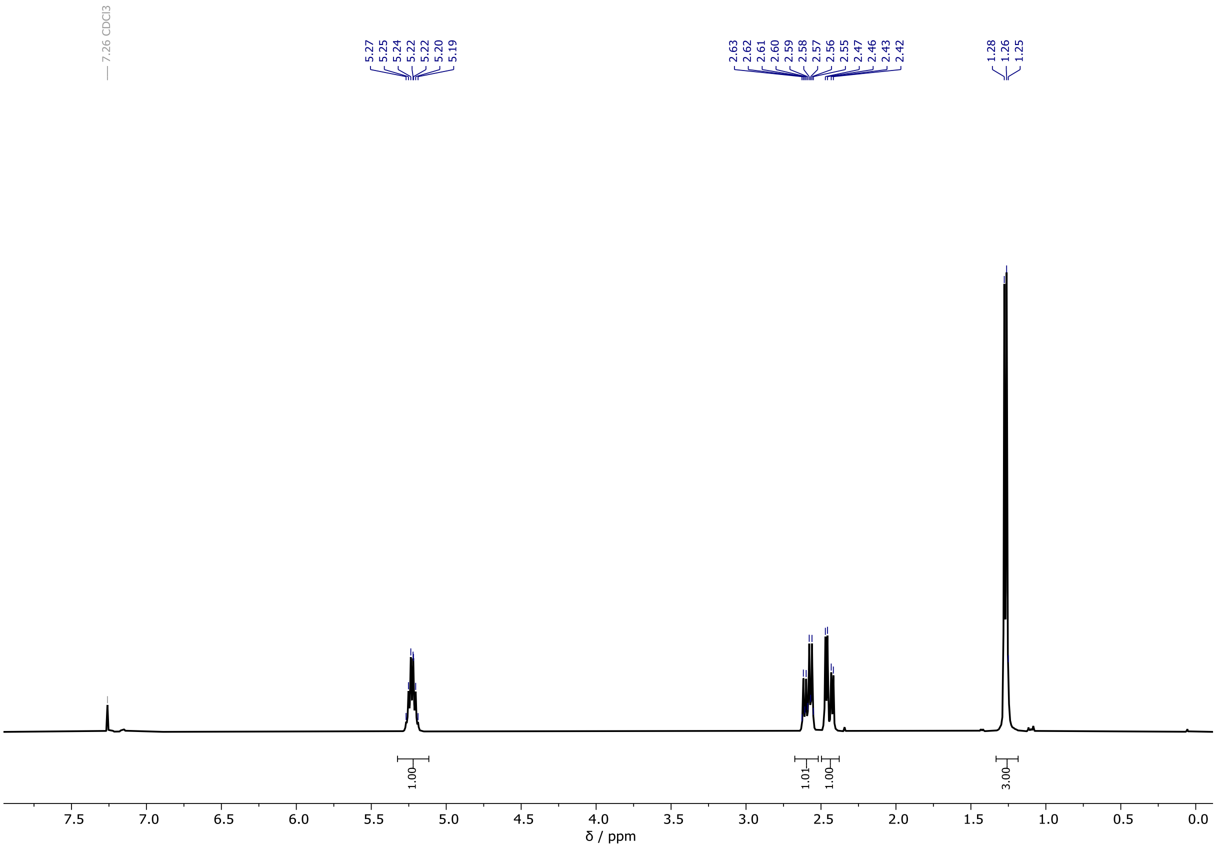

Figure S16: Representative ^1H NMR spectrum (400 MHz, $CDCl_3$, 300 K) of *syndio*-PHB (Table 1, entry 4).

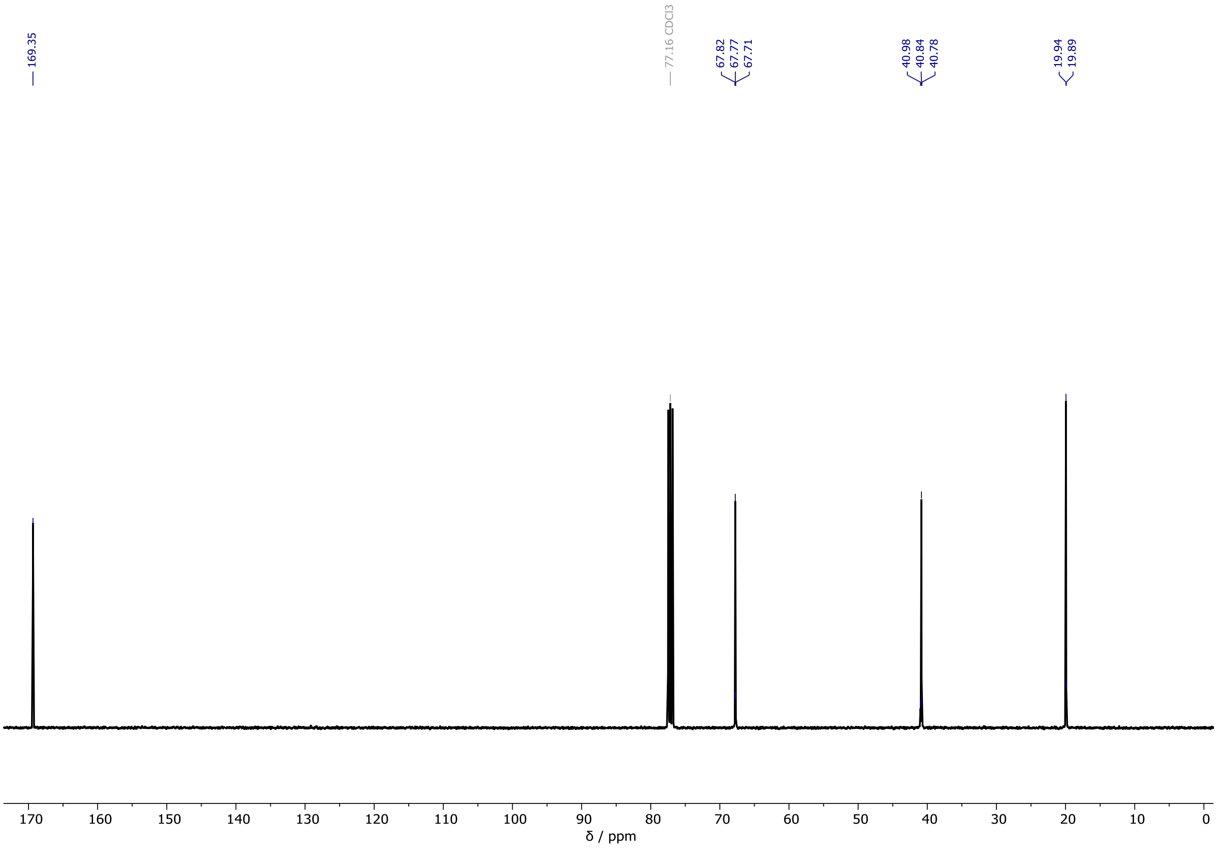

Figure S17: Representative $^{13}C\{^{1}H\}$ NMR spectrum (101 MHz, $CDCl_3$, 300 K) of *syndio*-PHB (Table 1, entry 4).

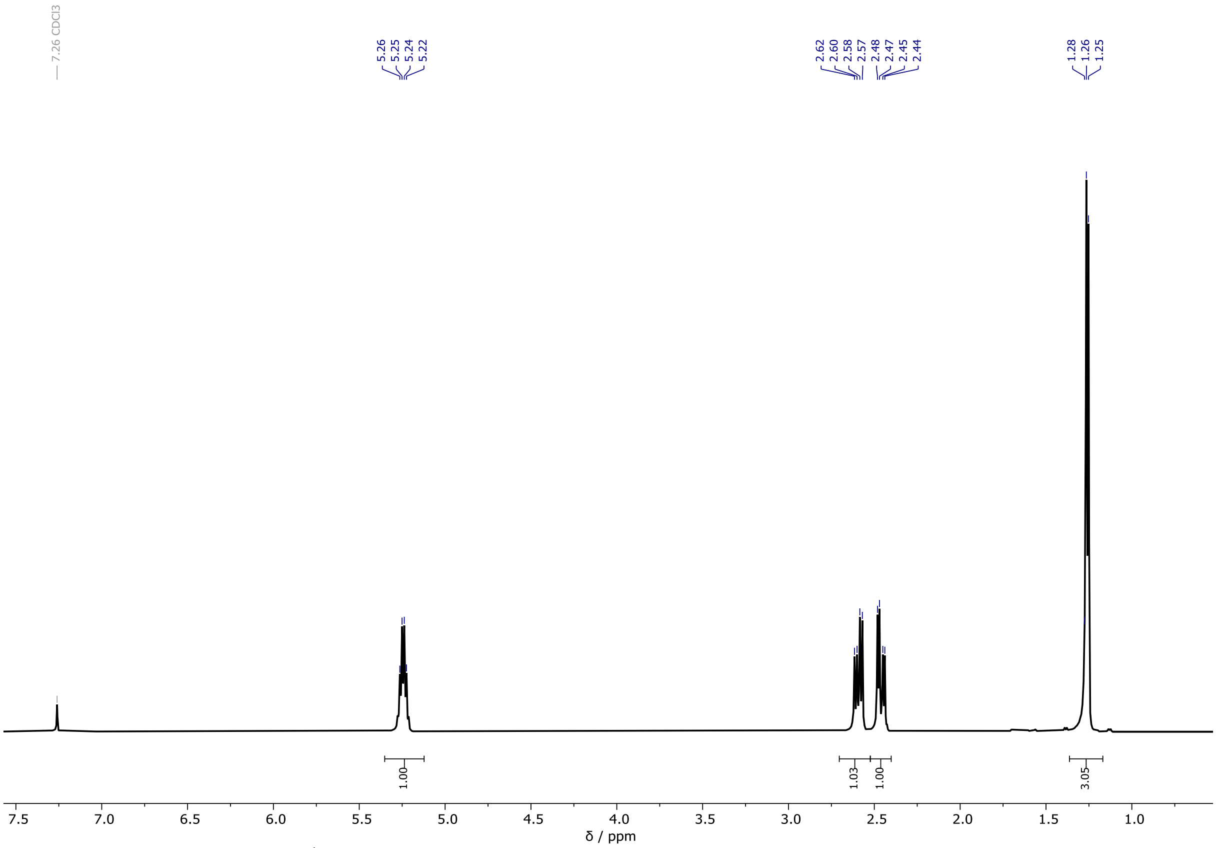

Figure S18: Representative ^{1}H NMR spectrum (400 MHz, $CDCl_3$, 300 K) of *iso*-PHB (Table 1, entry 6).

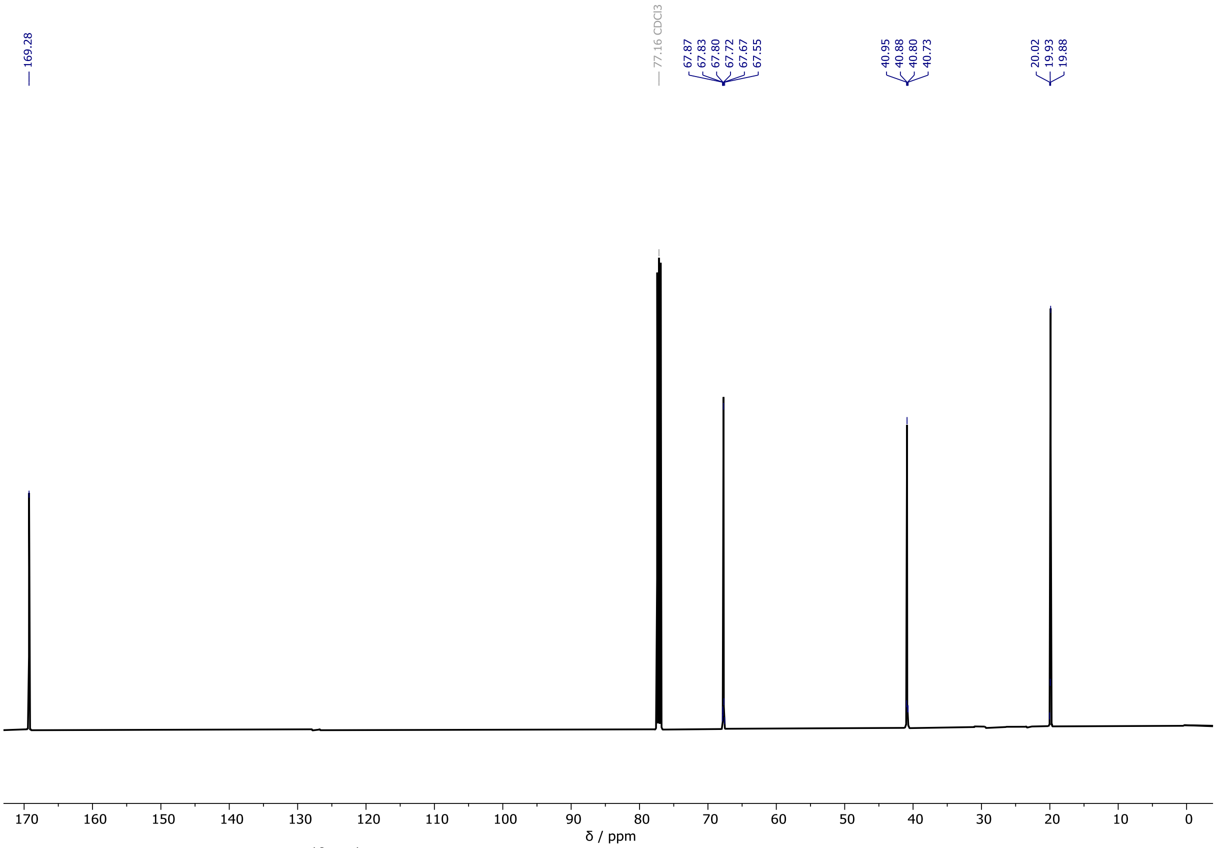

Figure S19: Representative $^{13}C\{^{1}H\}$ NMR spectrum (101 MHz, $CDCl_3$, 300 K) of *iso*-PHB (Table 1, entry 6).

2.2 Polymer Characterization Data

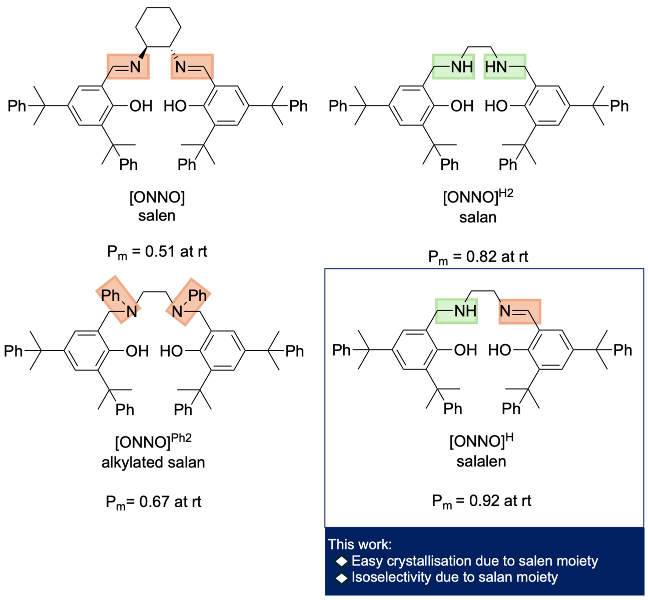

Scheme S2: Reported salen, salan, and alkylated salan ligands active in the stereoselective ROP of *rac* BBL.[3, 8]

Table S1: Ring-opening polymerization of BBL with salalen ligands.[a]

Run	Catalytic System	Monomer	t_R [min]	X [%]	$M_{n,theo}$ [kg·mol^{-1}]	$M_{n,rel}$[b] [kg·mol^{-1}]	$Đ$[b]	P_m[c]
**1**	**Y+L5**	(*S*)-BBL	5	64	11	27	1.9	n.d.
**2**	**Y+L5**	(*R*)-BBL	5	55	8	33	1.8	n.d.
**3**	**Y+L5**	*rac*-BBL + 1 eq. BnOH	20	95	16	42	1.3	0.57
**4**	**Y+L5**	*rac*-BBL + 2 eq. BnOH	20	92	8	8	1.1	0.50

[a]Reaction conditions: $[Monomer]_0$ = 2 M in toluene at room temperature, [b]Determined by SEC in $CHCl_3$ at 40°C relative to polystyrene standards. [c]Tacticity determined by ^{13}C NMR spectroscopy, integration of the carbonyl signal, n.d. not determined.

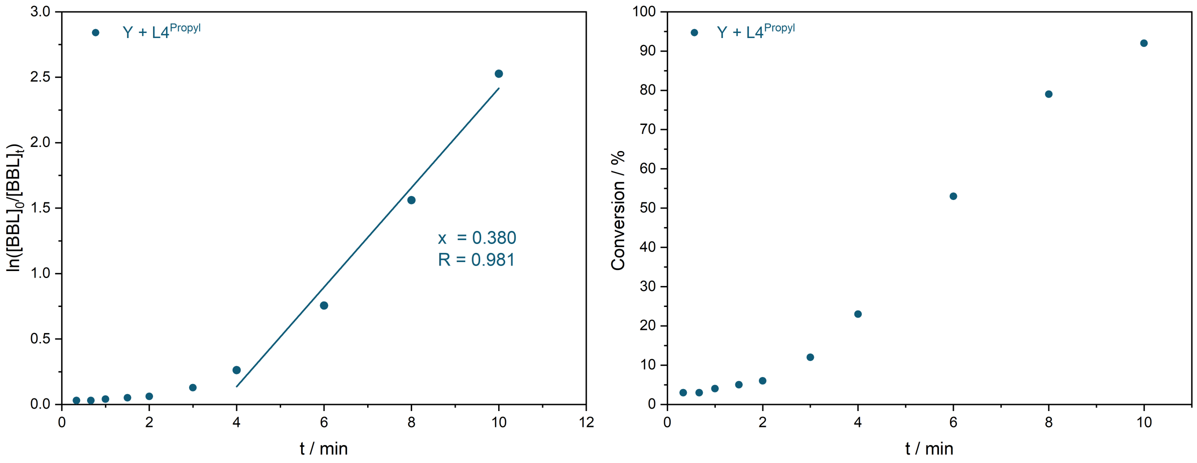

Figure S20: Semi-logarithmic plot of monomer concentration over time (left) and conversion over time (right) for the ROP of BBL mediated by **Y + L4**$^{\mathbf{Propyl}}$. Conditions: $[BBL]_0$ = 2.0 M, [BBL]/[Y + **Y + L4**$^{\mathbf{Propyl}}$] = 200/1 in Toluene at rt; M_n = 97 kg·mol^{-1}; P_m = 0.26.

2.3 Determination of Tacticity

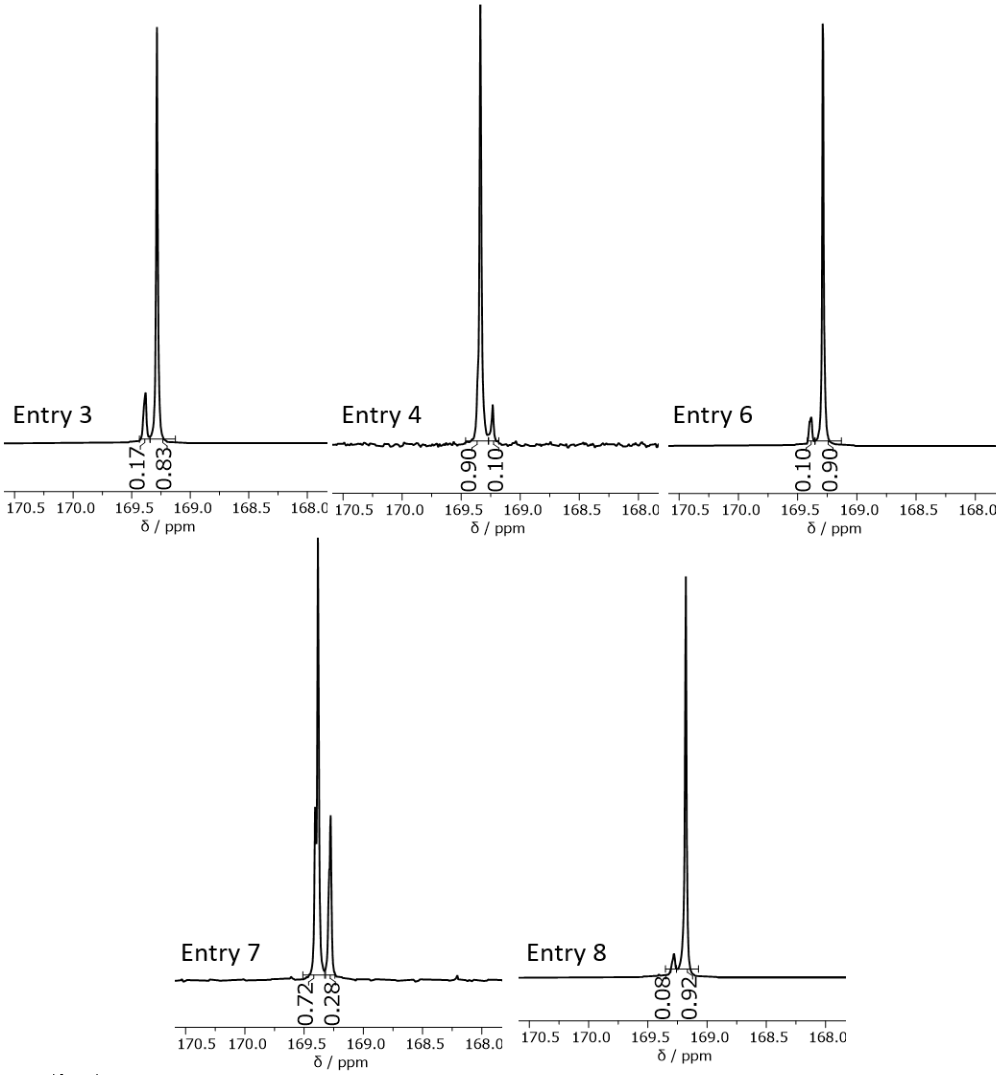

Figure S21: $^{13}C\{^{1}H\}$ NMR spectra (carbonyl region) of PHBs produced by *in situ* generated catalysts **Y+L3-4$^{\textbf{Cyclohexyl/Propyl}}$** and **L5** (Table 1).

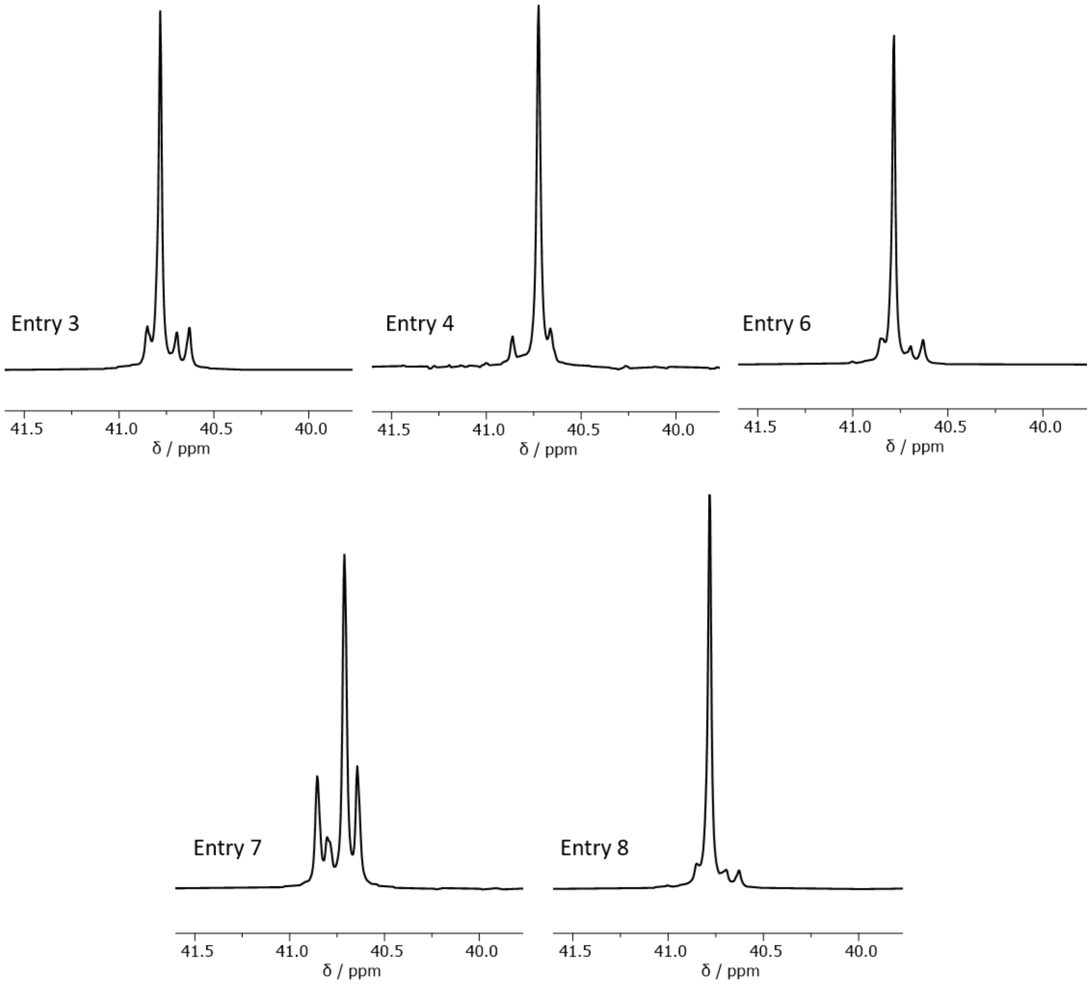

Figure S22: $^{13}C\{^1H\}$ NMR spectra (methylene region) of PHBs produced by *in situ* generated catalysts **Y+L3-4$^{\mathbf{Cyclohexyl/Propyl}}$** (Table 1).

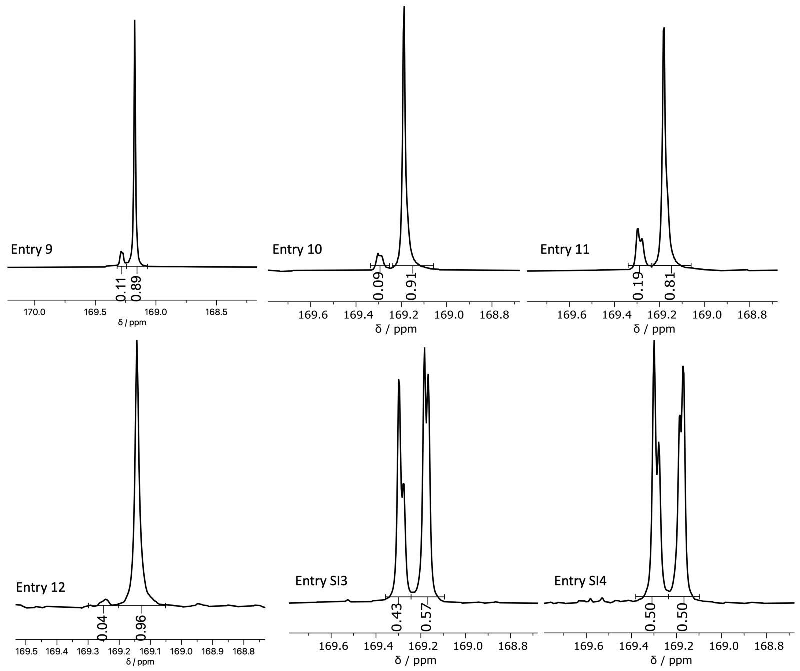

Figure S23: $^{13}C\{^1H\}$ NMR spectra (carbonyl region) of PHBs produced by catalysts **YL5** (Table 1 entries 9, 10, 11 and 12) and **YL5**+BnOH (Table S1, entries 3 and 4).

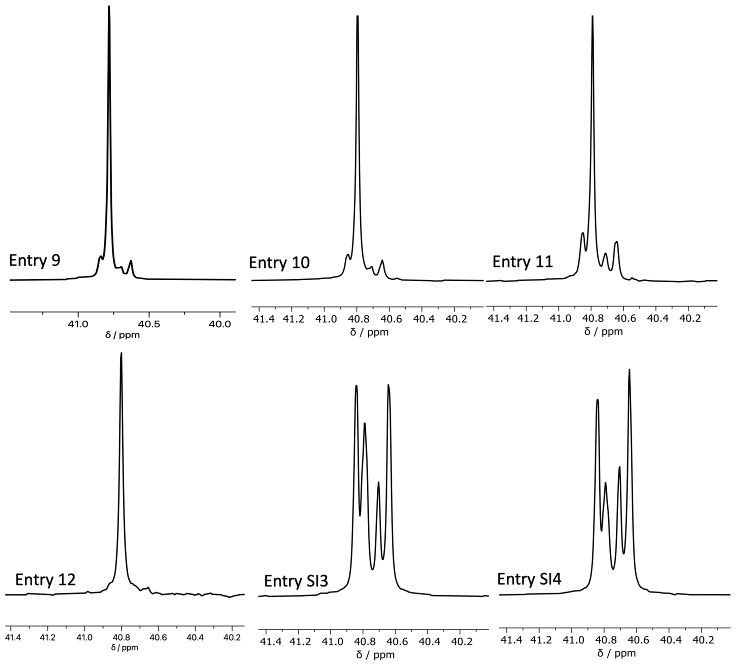

Figure S24: $^{13}C\{^1H\}$ NMR spectra (methylene region) of PHBs produced by catalysts **YL5** (Table 1 entries 9, 10, 11 and 12) and **YL5**+BnOH (Table S1, entries 3 and 4).

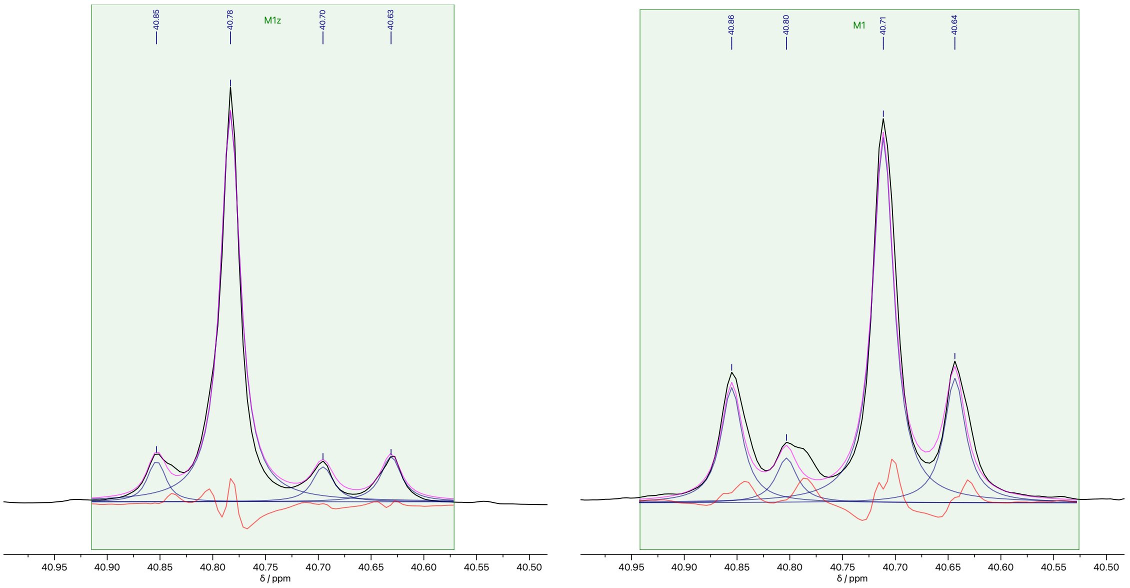

	ppm	Height	Width	L/G	Area
1	**40.8532**	**821.38**	**2.60**	**0.51**	**6226.29**
2	40.7833	7970.64	2.81	0.02	70033.38
3	40.6956	712.05	2.96	0.33	6308.26
4	40.6311	923.81	2.93	0.05	8414.75

	ppm	Height	Width	L/G	Area
1	**40.8550**	**1308.11**	**3.02**	**1.00**	**12377.46**
2	40.8032	509.27	3.02	1.00	4818.76
3	40.7114	4147.58	3.02	1.00	39244.63
4	40.6436	1417.46	3.02	1.00	13412.06

Figure S25: $^{13}C\{^1H\}$ NMR spectra (methylene region) of PHB with peak fit and integration to determine the Bernoulli distribution. Polymer produced by catalysts **Y+L3^{Cyclohexyl}** (left, table 1 entries 3) and **Y+L4^{Propyl}** (right, table S1, entries 7).

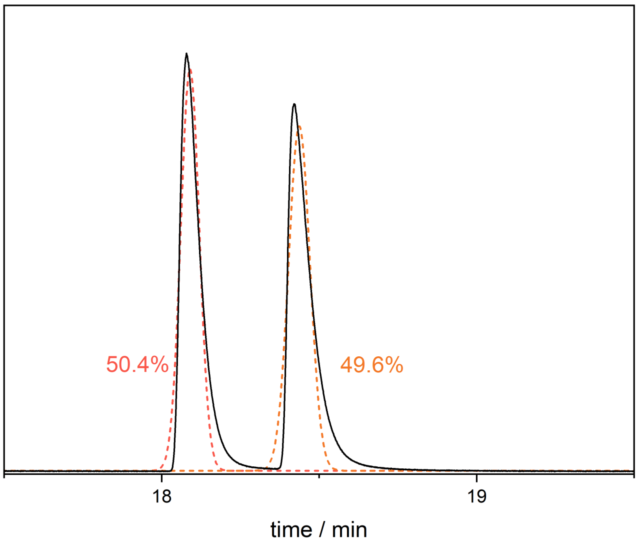

**Peak**	**RetTimer** [min]	**Width** [min]	**Area** [pA·s]	**Height** [pA]	**Area** [%]
1	18.08	0.0614	172.3	37.9	50.4
2	18.42	0.0677	170.7	33.2	49.6

Figure S26: Chiral GC-FID measurement of monomer surplus from polymerization **Y+L5** (Table 1, entry 8) with peak fitting to obtain the ratio of the racemic monomer mixture.

2.4 SEC Characterization of ROP Polymers

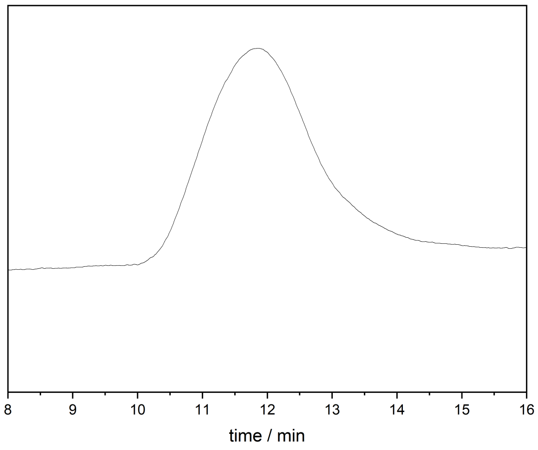

Figure S27: Representative molecular weight distribution of *iso*-PHB (Table 1, entry 6) determined *via* SEC in $CHCl_3$ at 40 °C against polystyrene standards using RI detection.

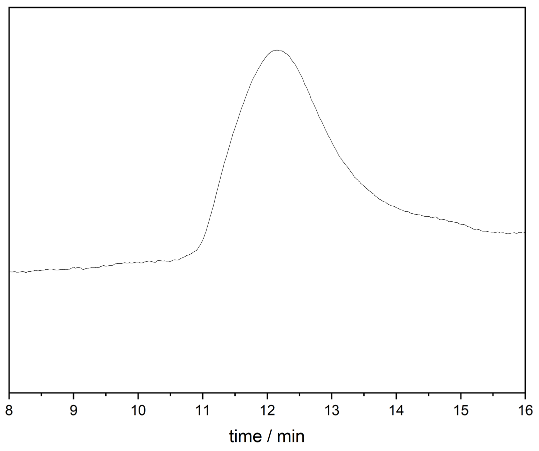

Figure S28: Representative molecular weight distribution of *syndio*-PHB (Table 1, entry 7) determined *via* SEC in $CHCl_3$ at 40 °C against polystyrene standards using RI detection.

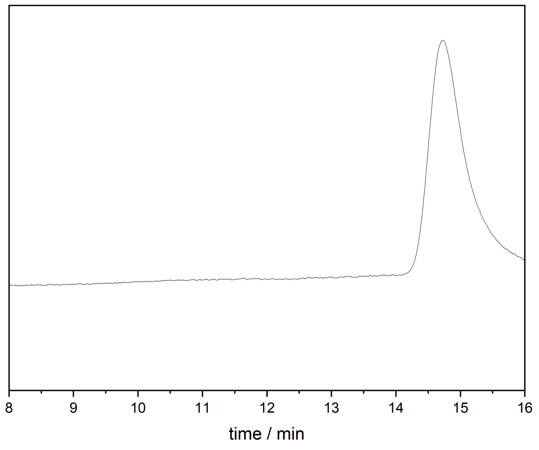

Figure S29: Representative molecular weight distribution of atactic PHB (Table S1, entry 4) determined *via* SEC in $CHCl_3$ at 40 °C against polystyrene standards using RI detection.

3 Additional Characterization Data

3.1 Characterization of the Catalyst YL5

2D-NMR Analysis of Compound YL5

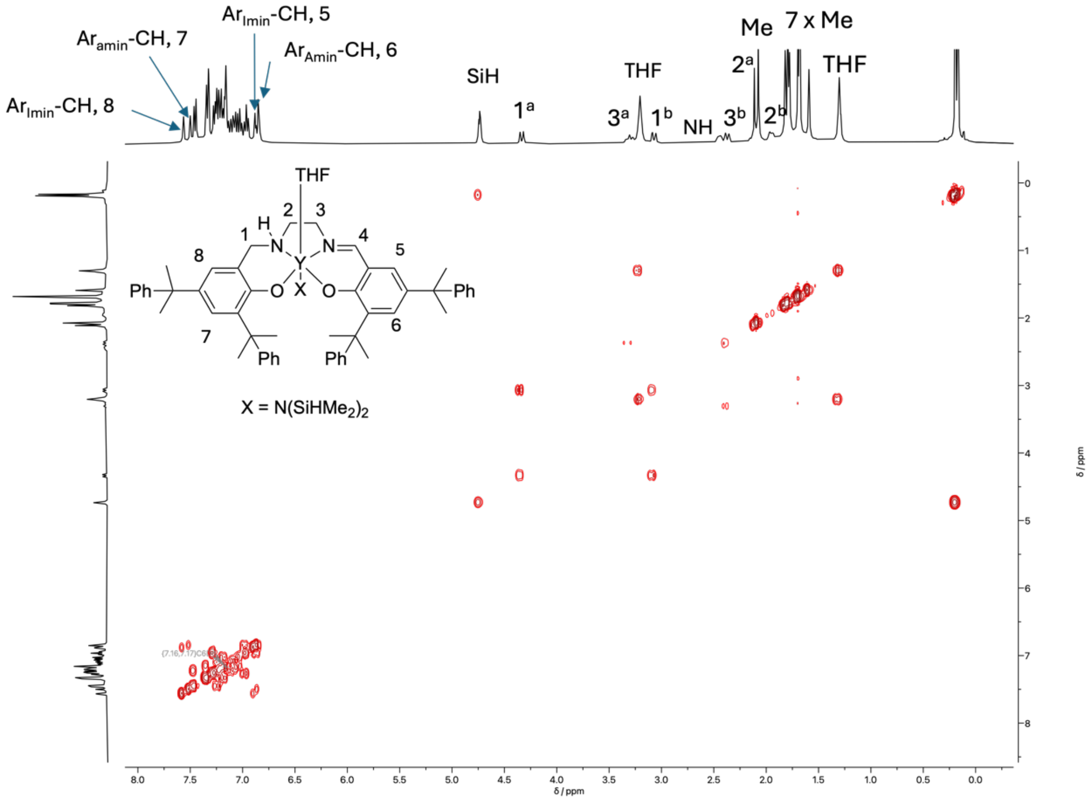

Figure S30: ^{1}H-COSY NMR of catalyst **YL5** and assignment of the corresponding protons.

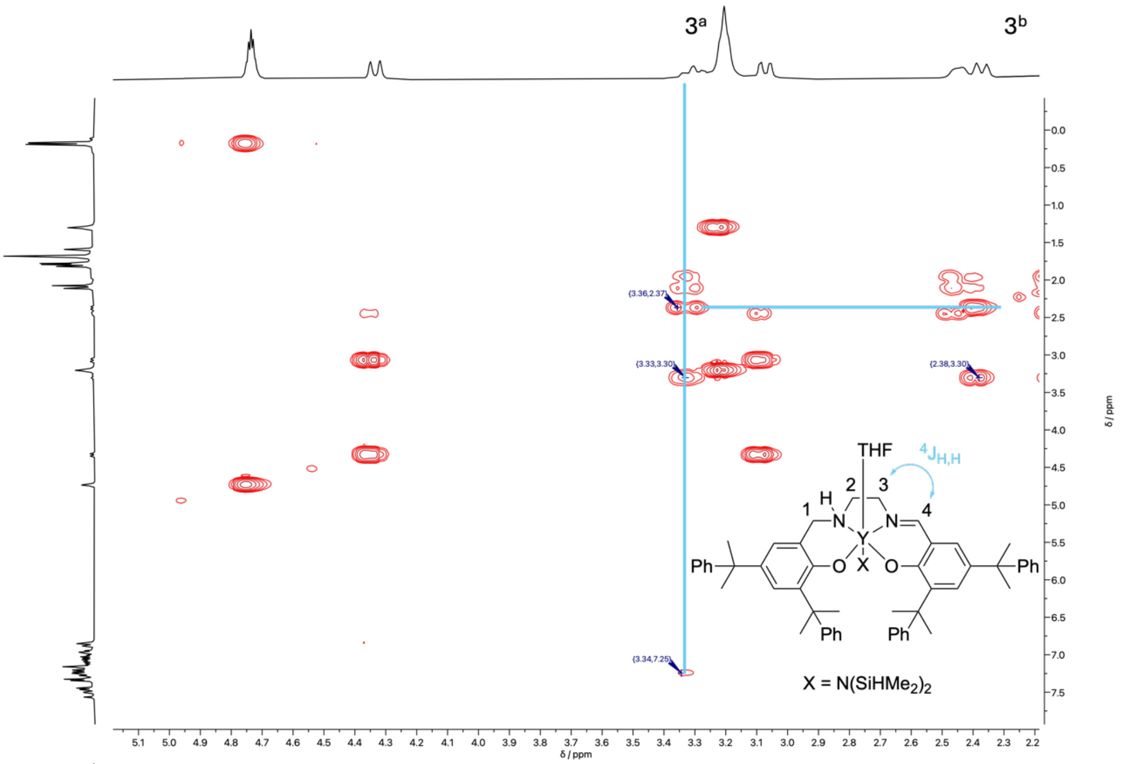

Figure S31: ^{1}H-COSY NMR of catalyst **YL5** and assignment of the corresponding protons in the backbone of the ligand with the proton next to the imin moiety.

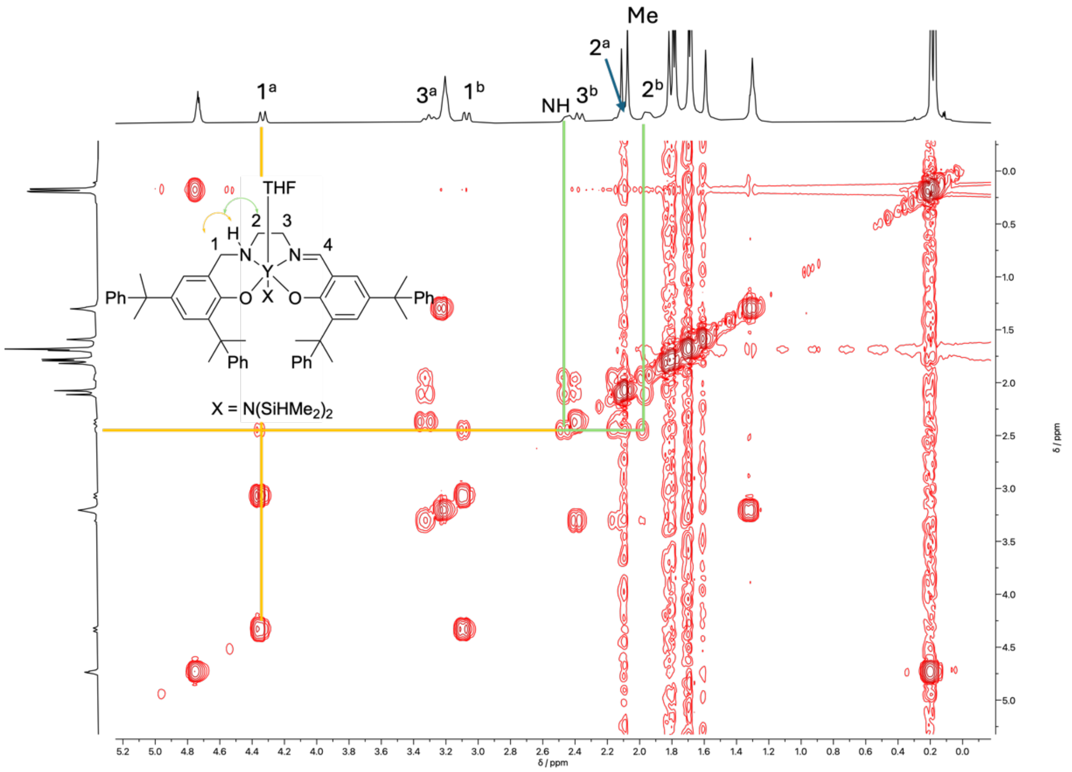

Figure S32: ^{1}H-COSY NMR of catalyst **YL5** and assignment of the corresponding protons in the backbone of the ligand and the protons next to the amine moiety.

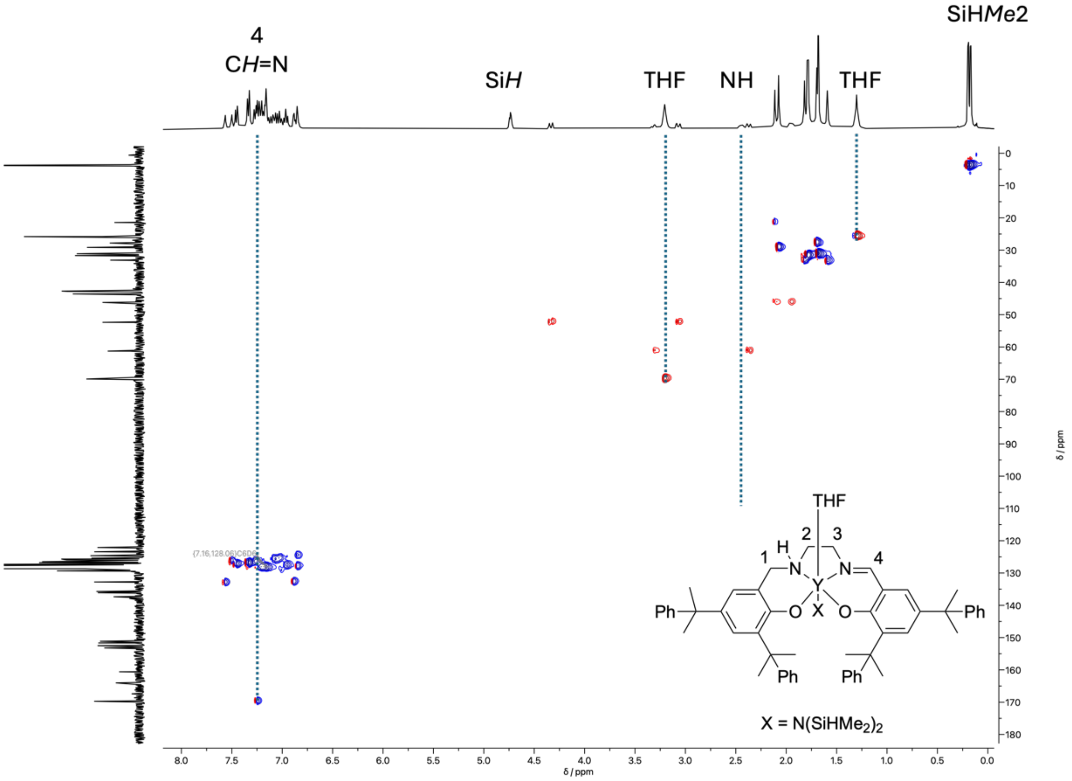

Figure S33: ^{1}H^{13}C-HSQC NMR of catalyst **YL5** and assignment of protons to corresponding C atoms. Assignment of NH signal as no coupling with a C atom is present in the ^{13}C NMR.

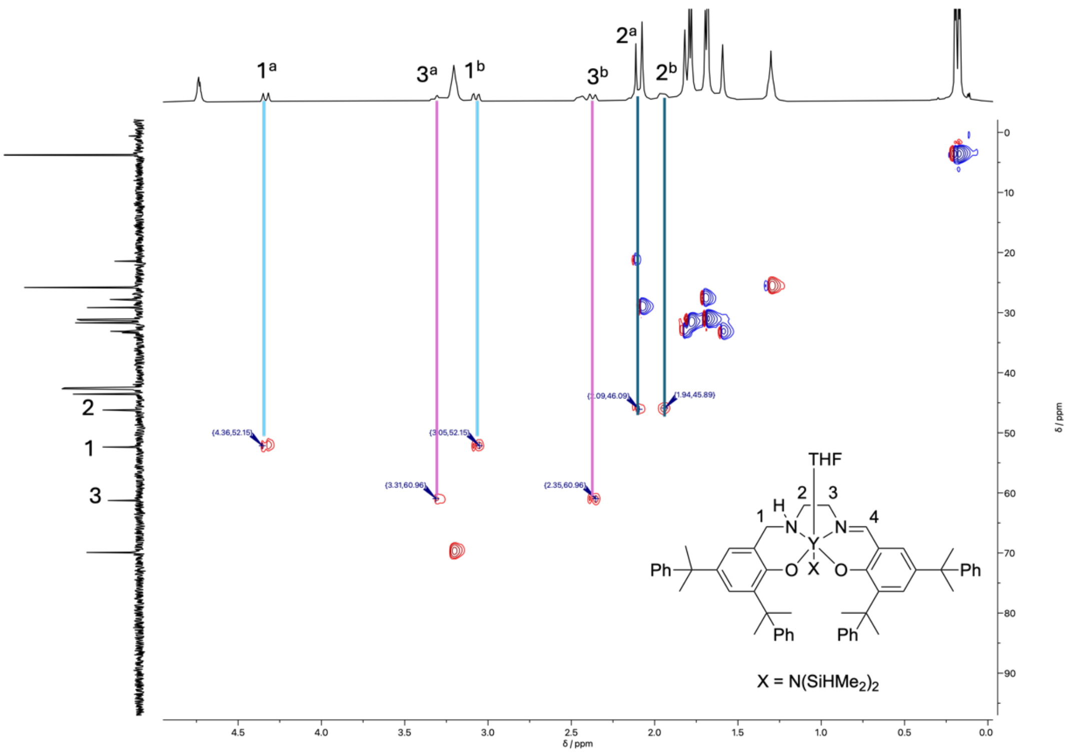

Figure S34: $^{1}H^{13}C$-HSQC NMR of catalyst **YL5** to assign the protons of the ligand backbone to the corresponding ^{13}C NMR.

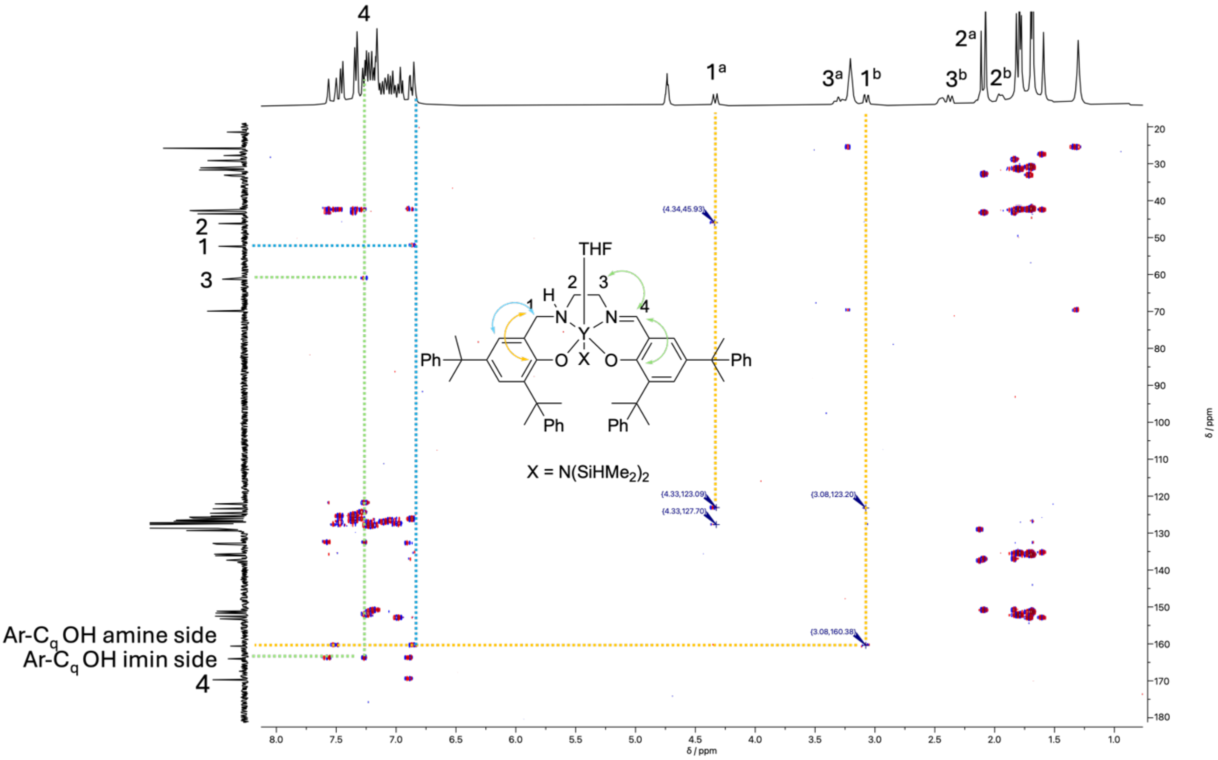

Figure S35: $^{1}H^{13}C$-HMBC NMR of catalyst **YL5** to further evaluate the protons at the aryl moieties.

DOSY NMR Analysis of Compound YL5

DOSY NMR measurements were performed to elucidate the nuclearity of **YL5** in solution under conditions relevant for polymerization runs. The molecular weight of the compounds was determined by using external calibration curves with normalized diffusion coefficients.[9] Toluene-d^8 was used as solvent, and the diffusion coefficient of the residual solvent resonance was used as an internal reference for calculations. An external calibration curve for dissipated spheres and ellipsoids was chosen.[8] The observed diffusion coefficients of the analytes and the internal reference are given in Figures S36. A molecular weight of 1065.9 g·mol^{-1} was determined, respectively, indicating that the compound is mononuclear in solution.

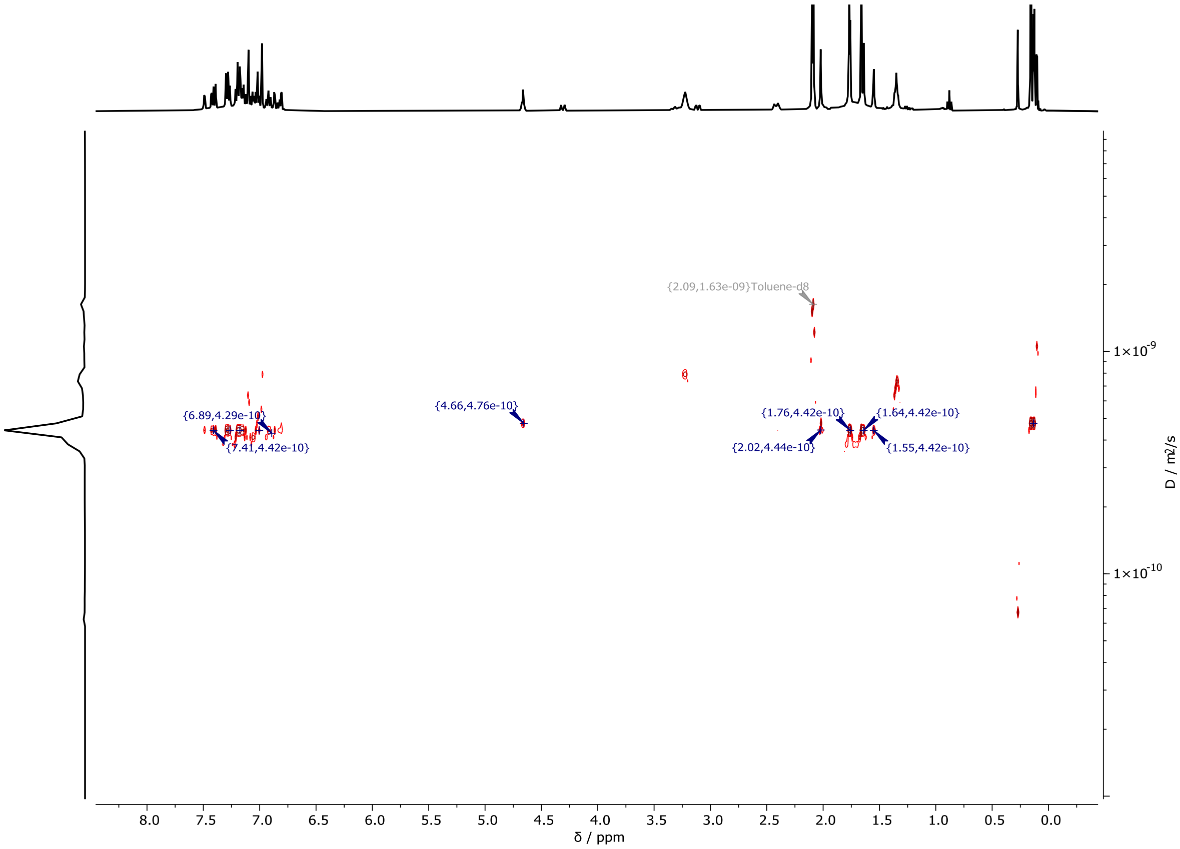

Figure S36: ^{1}H-DOSY NMR of compound **YL5** in toluene-d_8 at room temperature.

LIFDI-MS (Liquid Injection Field Desorption Ionization)

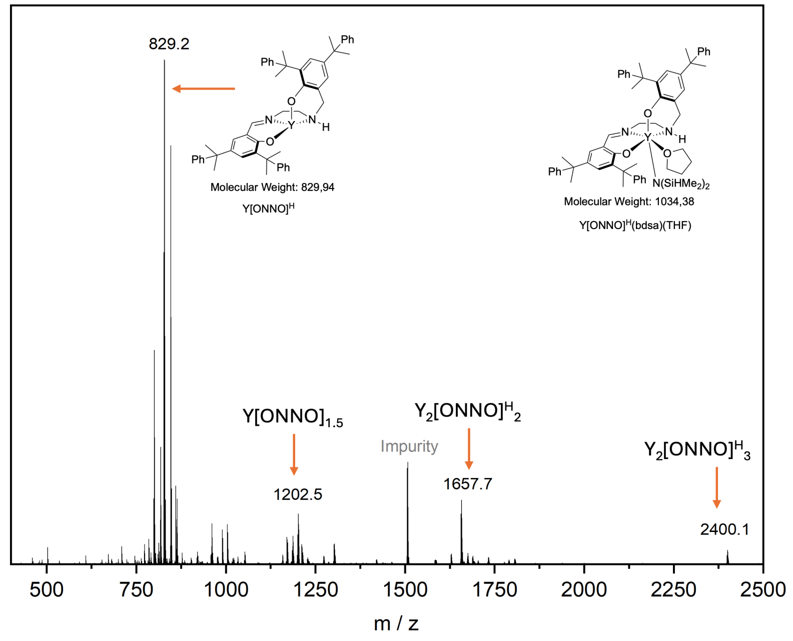

Figure S37: LIFDI-MS of the isolated catalyst **YL5** and assigned signals of the corresponding fragments. Signals at *m/z* ratio 1500 are impurities from the device.

3.2 Kinetic Parameters

The kinetic isotope effect was calculated by determining the kinetic rate constant for the ROP of *rac*-BBL. Polymerization was conducted as mentioned earlier, however at the respective time, an aliquot was taken and evaluated via ^{1}H-NMR to obtain the conversion.

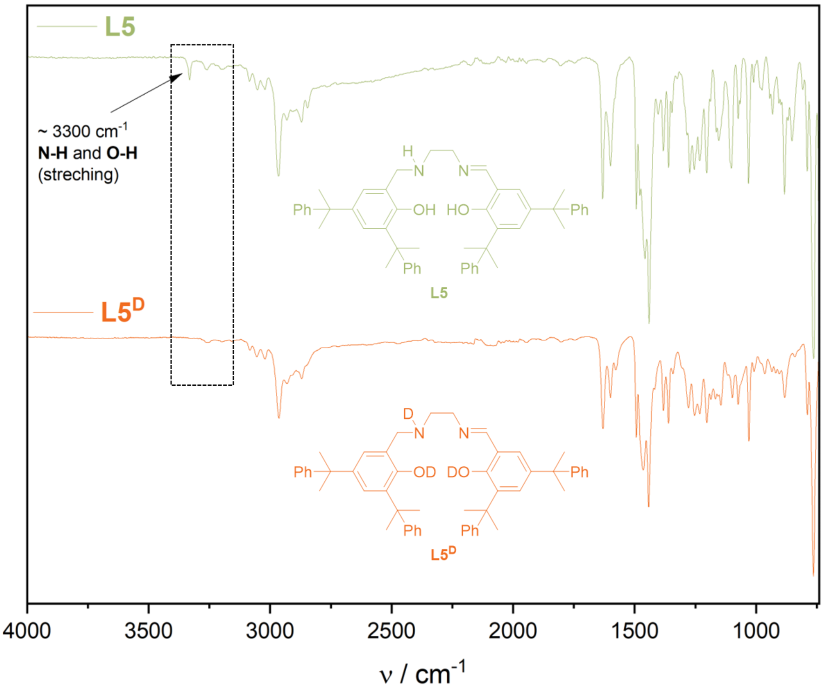

Figure S38: Stacked IR spectra of **L5** (green) and **L5^{D}** (orange) showing the absence of characteristic NH/OH vibrations after deuteration.

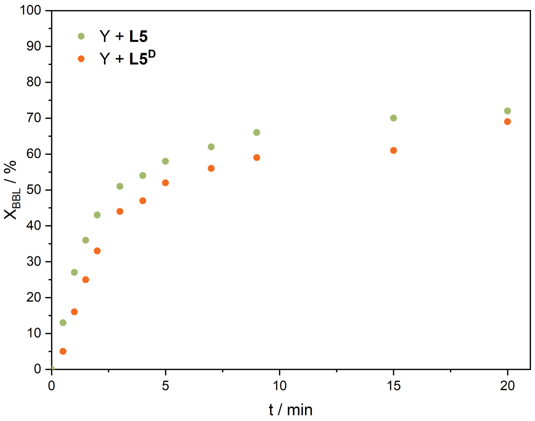

Figure S39: Ring-opening polymerization via the *in situ* approach of *rac*-BBL with **Y+L5** and **Y+L5[D]** in toluene at room temperature**.** Conversions were determined *via* ^{1}H NMR spectroscopy.

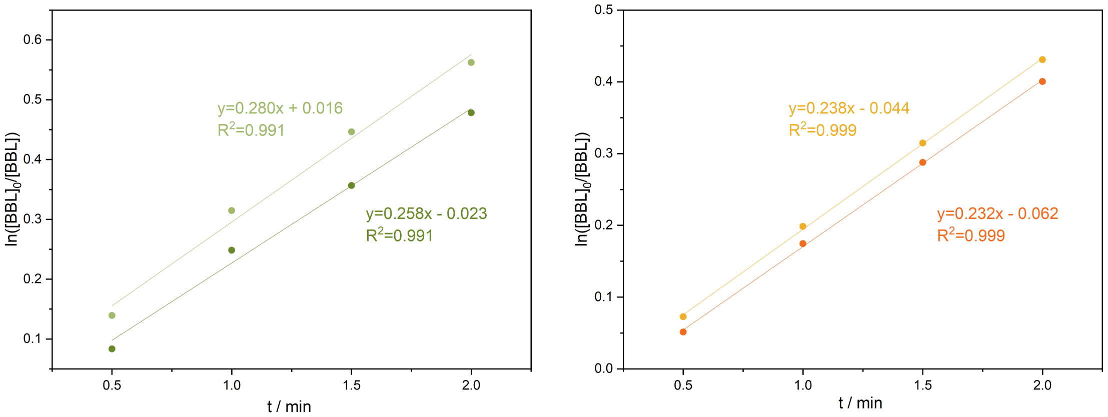

Figure S40: First-order plot with linear fit for *in situ* polymerization of *rac*-BBL initiated by **Y+L5** (left) or **Y+L5[D]** (right). Polymerization was conducted in toluene at room temperature, and a $[BBL]_0$ = 2 M. Conversion was determined by ^{1}H NMR spectroscopy.

Table S2: Calculation of the kinetic rate constant k_{obs} for the ROP of *rac*-BBL.[a]

Run	Catalytic System	k_{obs} [10^{-3} s^{-1}]	$M_{n,rel}$[b] [kg·mol^{-1}]	$Đ$[b]	P_m[c]
1	**Y+L5**	4.667	44	1.8	0.92
2	**Y+L5**	4.300	49	1.7	0.93
3	**Y+L5[D]**	3.965	59	1.6	0.85
4	**Y+L5[D]**	3.870	57	1.8	0.93
5	**YL5**	2.400	72	1.4	0.88
6	**YL5[D]**	1.867	54	1.4	0.92
**MW k^{H}_{obs}**	**Y+L5**	4.483 ± 0.18			
**MW k^{D}_{obs}**	**Y+L5[D]**	3.917 ± 0.05			

[a] Reaction conditions: $[BBL]_0$ = 2 M in toluene at room temperature, [b] Determined by SEC in $CHCl_3$ at 40°C relative to polystyrene standards. [c] Tacticity determined by ^{13}C NMR spectroscopy, integration of the carbonyl signal.

4 Computational Details

Density functional theory (DFT) calculations were carried out using the B3LYP hybrid functional,[10] which integrates a portion of exact Hartree-Fock exchange with density-based approximations. This functional is widely recognized for its effectiveness in balancing computational cost and accuracy across diverse systems. To include the description of dispersion interactions, Grimme's D3 correction[11] was used during optimizations, accounting for long-range van der Waals forces. The efficient second generation def2-TZVP basis set, developed by Ahlrichs and co-workers,[12] of triple-zeta valence quality with polarization functions, was applied to all atoms. For Y, the SDD effective core potential[13] was used to include relativistic effects and core electron treatment. Since Y^{3+} has a closed-shell electronic configuration, spin-restricted calculations were performed. Additionally, the resolution of identity (RI) approximation[14] was employed in the self-consistent field (SCF) procedure during geometry optimizations to enhance computational efficiency.

Solvent effects were incorporated by modelling the toluene solution using the conductor-like screening model (COSMO),[15] a variant of the polarizable continuum model (PCM).[16, 17] In this approach, the solvent is represented as a polarizable dielectric continuum, characterized by its dielectric constant, which was set to 2.4 to reflect the properties of toluene. The default COSMO parametrization in Turbomole was employed to ensure reliable and consistent solvation effects. For gas-phase calculations, vibrational normal mode analysis was conducted to confirm that structures correspond to true minima on the potential energy surface and to obtain the thermal corrections to the free energy. The latter were added to the energies in solution using a thermodynamic cycle. This enabled the determination of Gibbs free energies in solution, which will be discussed in the following sections. For simplicity, we will refer to it as energy. Because of the size of the complex the gas-phase calculations and the vibrational analysis were performed with a single valence plus polarization basis set, def2-SV(P). All computations were carried out using the Turbomole software package, version 7.7.[18, 19]

The ligand under investigation is a tetradentate [ONNO]-type system, incorporating both an amine-phenolate, salan, and an imine-phenolate, salene, functionalities. Given its sequential O-N-N-O donor arrangement, this ligand can adopt multiple binding modes around an octahedral Y^{3+} center. Due to the presence of amine and imine groups, the ligand is non-symmetric, allowing for four feasible coordination isomers, which can be described using the *fac-mer* terminology: *fac-fac, fac-mer, mer-fac,* and *mer-mer.* To determine the most stable coordination mode, all four isomers were computationally optimized and compared based on their relative stability. The optimized structures are depicted in figure S41.

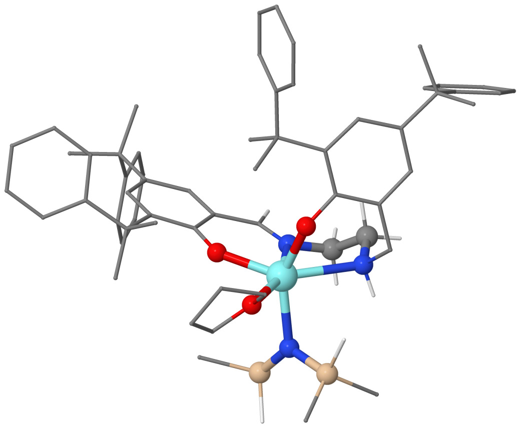

fac-mer
0

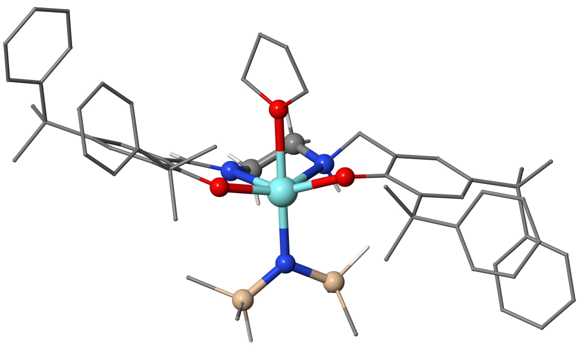

mer-mer
19

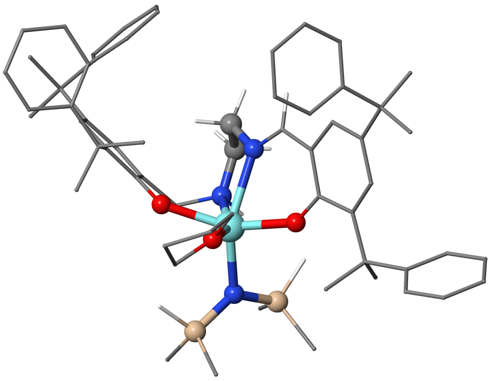

fac-fac
36

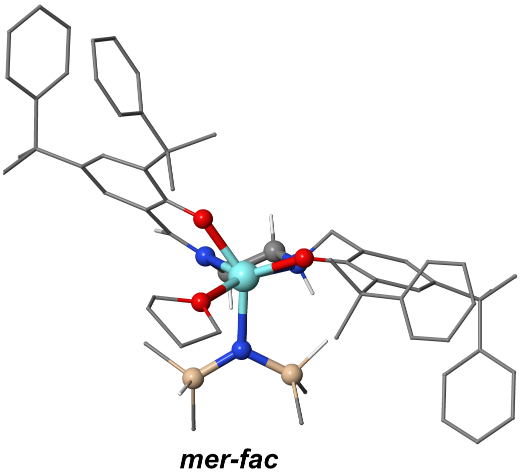

mer-fac
55

Figure S41. Optimized **YL5** complexes and their relative stability in kJ·mol^{-1}. Most hydrogen atoms omitted for clarity.

The *fac-mer* isomer, characterized by *fac*-bound salan and *mer*-bound salene moieties, exhibited the highest stability. Other isomers were significantly less stable. The *fac-mer* isomer is more stable than the second most stable *mer-mer* isomer by 19 kJ·mol^{-1}. The *fac-fac* isomer is less stable by 36 kJ·mol^{-1}. The *mer-fac* isomer with the salene fragment in a *fac* mode exhibited the greatest steric strain and is disfavored by 55 kJ·mol^{-1}. Conformational analysis included evaluation of *tert*-butyl phenyl group rotation at the *para* and *ortho* positions of the coordinated phenolate moieties. These rotations resulted in destabilization of the complexes, with energy increases of up to 15 kJ·mol^{-1} (*fac-mer* and *mer-fac*), 16 kJ·mol^{-1} (*fac-fac*), and 19 kJ·mol^{-1} (*mer-mer*). Furthermore, the relative positioning of the THF and bdsa ligands with respect to the amine hydrogen was investigated. For the *fac-mer* isomer, placement of the bdsa group within the equatorial plane, along with the *mer*-bound salene, destabilized the complex by 110 kJ·mol^{-1}. In both the *mer-mer* and *fac-fac* isomers, *cis* positioning of the amine hydrogen relative to the bdsa ligands was preferred. Alternative arrangements, involving exchange of byligand positions, resulted in destabilization of 18 kJ·mol^{-1} (*mer-mer*) and 27 kJ·mol^{-1} (*fac-fac*).

The optimized structures of all isomers reveal a distorted octahedral coordination environment around Y^{3+}, primarily due to steric constraints imposed by the ligand framework. In the optimized geometry of the most stable *fac-mer* isomer, the angle subtended by the phenolate oxygen atom and the *meridional* plane defined by the salalene nitrogen atoms and the Y^{3+} center is 66°, a deviation from the ideal 90° orthogonality. In the *mer-fac* isomer, the corresponding angle between the *fac*-positioned phenolate group of salene and the *meridional* plane is further reduced to 58°. In the *mer-mer* isomer, ideal coplanarity of the salalene donor atoms would correspond to a 0° dihedral angle. However, in the optimized structures, the observed dihedral angles are 19° for the salene moiety and 16° for the salan moiety, indicating deviations from perfect coplanarity. Furthermore, the *fac-fac* isomer exhibits similar distortion, with the axial salalene oxygen atoms forming an angle of 150° via central metal. The deviations from orthonormality for the phenolate moieties in this isomer are 14° and 37° for the salene bound phenolate. These distortions from an ideal octahedral geometry highlight the steric effects governing the coordination preferences of the ligand, ultimately stabilizing the *fac-mer* configuration.

5 Cartesian coordinates of YL5 isomers

fac-mer YL5(THF)(bdsa)

147

Y	1.9578958	-0.3757755	0.4874316
N	3.7653381	-1.6136908	-0.1900951
Si	3.8054522	-3.3184960	-0.3178720
H	5.2200992	-3.8165611	-0.3741972
Si	5.0941778	-0.5989875	-0.4994875
H	4.6059244	0.8054137	-0.2416780
C	2.9582038	-3.9647139	-1.8748623
H	3.3552690	-3.4846050	-2.7730079
H	3.1005020	-5.0443872	-1.9775006
H	1.8828007	-3.7737404	-1.8331655
C	5.6862534	-0.6262295	-2.2916845
H	6.4702592	0.1154194	-2.4674730
H	6.0940085	-1.6111727	-2.5377653
H	4.8726525	-0.4287772	-2.9954821
C	6.5912257	-0.8436657	0.6153840
H	7.4041737	-0.1686822	0.3338462
H	6.3393731	-0.6490023	1.6599230
H	6.9625827	-1.8693235	0.5443264
C	2.9806931	-4.1500302	1.1566460
H	3.4950230	-3.9018614	2.0879974
H	1.9430753	-3.8213104	1.2536473
H	2.9837457	-5.2378371	1.0468383
O	3.2418926	0.1330057	2.4672858
C	3.5263492	1.4311436	3.0378751
C	3.7157602	-0.8840851	3.3867382
C	3.2899934	1.2249437	4.5240683
H	4.5671353	1.6918113	2.8184445
H	2.8581611	2.1400139	2.5564327
C	3.8218195	-0.2019470	4.7627988
H	2.9923803	-1.6945581	3.3590866
H	4.6748964	-1.2572937	3.0274591
H	2.2214652	1.2774161	4.7329507
H	3.7977824	1.9719685	5.1335388
H	3.2406039	-0.7307331	5.5166597
H	4.8601398	-0.1786474	5.0945142
O	1.2276898	1.6295086	0.8485001
O	0.6698069	-1.5858515	1.7297036
C	0.5684090	2.5816781	0.2068120
C	-0.5851179	3.2008171	0.7460779
C	1.0043385	2.9595390	-1.0871855
C	-1.2458098	4.1539690	-0.0355281
C	0.3110051	3.9125388	-1.8151514
C	-0.8265699	4.5360385	-1.3058190
H	-2.1382311	4.6012473	0.3701914
H	0.6483552	4.1491705	-2.8188566
C	-0.4536743	-2.2521121	1.7532245
C	-1.0375977	-2.6920821	2.9727475
C	-1.1188092	-2.5814902	0.5314689
C	-2.1663214	-3.5023351	2.9076678
C	-2.2738550	-3.3779765	0.5458287
C	-2.8072413	-3.8696608	1.7200480
H	-2.5677922	-3.8680299	3.8394384
H	-2.7379925	-3.6287061	-0.4019493
C	-0.6611649	-2.1492618	-0.7666788

H	-1.2840797	-2.5054608	-1.5941221
C	2.1970829	2.2654353	-1.6871686
H	3.0816157	2.4097767	-1.0625798
H	2.4088798	2.6973155	-2.6714492
N	2.0109772	0.7900497	-1.8025891
N	0.3550485	-1.4128461	-1.0522879
C	0.8461728	0.3958295	-2.6152391
H	0.9815350	0.6510717	-3.6730223
H	-0.0189238	0.9508813	-2.2514174
C	0.5977379	-1.0976355	-2.4604174
H	-0.2387258	-1.4068702	-3.0968112
H	1.4850008	-1.6487892	-2.7874255
H	2.8441328	0.4106154	-2.2446455
C	-4.0738715	-4.7385469	1.6869751
C	-0.3823814	-2.3970133	4.3412347
C	-1.1869743	2.7437663	2.0953488
C	-1.5691920	5.5697953	-2.1654223
C	-0.1244322	2.6504617	3.2217874
H	0.5545895	1.8285183	3.0309461
H	0.4655839	3.5642726	3.2981063
H	-0.6218854	2.4859353	4.1798836
C	-1.7691334	1.3284033	1.8859731
H	-2.5092073	1.3126577	1.0844021
H	-0.9703810	0.6410077	1.6189191
H	-2.2393586	0.9543962	2.7983423
C	-0.6439206	6.7861641	-2.3707031
H	-1.1435951	7.5823512	-2.9272623
H	-0.3579515	7.1865584	-1.3969061
H	0.2696746	6.5168174	-2.9023626
C	-2.8595989	6.0817323	-1.4908245
H	-3.3550226	6.7966122	-2.1501581
H	-3.5622851	5.2732660	-1.2871188
H	-2.6359530	6.5871117	-0.5488513
C	0.0978346	-0.9296713	4.4831444
H	0.9016491	-0.7087036	3.7923828
H	-0.7043889	-0.2222914	4.2842059
H	0.4460831	-0.7664722	5.5055149
C	0.8465270	-3.3205288	4.4572769
H	0.5596613	-4.3729939	4.4378749
H	1.5034568	-3.1473935	3.6085145
H	1.4065784	-3.1273026	5.3756926
C	-4.3565120	-5.4173883	3.0443896
H	-3.5157608	-6.0296408	3.3729286
H	-4.5696616	-4.6801940	3.8208340
H	-5.2286367	-6.0661641	2.9499489
C	-5.2688075	-3.8156294	1.3699192
H	-5.3176219	-3.0228097	2.1179959
H	-5.1650716	-3.3454536	0.3907137
H	-6.2152438	-4.3601119	1.3951919
C	-2.2637883	3.7347889	2.5654367
C	-1.8886620	5.0151596	2.9940226
C	-3.6247632	3.4299407	2.5753082
C	-2.8277809	5.9468634	3.4131105
H	-0.8441897	5.2973721	2.9760076
C	-4.5745746	4.3606275	2.9961113
H	-3.9683347	2.4596344	2.2467446
C	-4.1827638	5.6236021	3.4172102
H	-2.5025174	6.9293003	3.7342824
H	-5.6241996	4.0915133	2.9888940
H	-4.9192416	6.3482383	3.7422749
C	-1.9741132	4.9061562	-3.4925071
C	-2.6556135	3.6829975	-3.4575732

C	-1.7221472	5.4719964	-4.7416304
C	-3.0745077	3.0540154	-4.6208633
H	-2.8451267	3.2138346	-2.4998866
C	-2.1363517	4.8419356	-5.9155240
H	-1.1954728	6.4129445	-4.8187920
C	-2.8152046	3.6323009	-5.8622410
H	-3.6015941	2.1089840	-4.5613035
H	-1.9238818	5.3037477	-6.8725291
H	-3.1374244	3.1429207	-6.7732738
C	-1.3893803	-2.6265014	5.4802465
C	-1.2150493	-3.5925923	6.4693113
C	-2.5368622	-1.8244903	5.5545371
C	-2.1469366	-3.7553535	7.4950950
H	-0.3494393	-4.2390238	6.4542581
C	-3.4688803	-1.9826296	6.5695482
H	-2.7151558	-1.0798937	4.7889677
C	-3.2776327	-2.9531963	7.5512895
H	-1.9824463	-4.5167241	8.2482370
H	-4.3474052	-1.3491133	6.5953878
H	-4.0024779	-3.0800617	8.3459275
C	-3.8949985	-5.8520096	0.6423588
C	-4.8311808	-6.1205204	-0.3556364
C	-2.7526659	-6.6604305	0.6988936
C	-4.6352180	-7.1582305	-1.2673853
H	-5.7272028	-5.5218327	-0.4404729
C	-2.5534664	-7.6957218	-0.2033285
H	-2.0006276	-6.4612767	1.4525464
C	-3.4974818	-7.9503428	-1.1966021
H	-5.3775503	-7.3410407	-2.0351655
H	-1.6580136	-8.3019120	-0.1364571
H	-3.3434241	-8.7544726	-1.9055360

mer-mer YL5(THF)(bdsa)

147

Y	1.2381226	-0.4838026	-0.0366673
N	1.6623150	-2.6004557	-0.7102830
Si	1.1878772	-4.0037712	0.1646273
H	1.2768400	-5.2174747	-0.7138068
Si	2.5039661	-2.8092648	-2.1935395
H	3.0294223	-1.4798529	-2.6200181
C	-0.5903678	-3.9387333	0.7758271
H	-1.2841748	-3.7593127	-0.0486593
H	-0.8606338	-4.8873442	1.2488378
H	-0.7285034	-3.1434508	1.5089182
C	1.3674322	-3.3765985	-3.5945094
H	1.9147610	-3.4684786	-4.5372578
H	0.9244756	-4.3492500	-3.3637224
H	0.5457366	-2.6716000	-3.7522671
C	3.9669822	-3.9942697	-2.1224846
H	4.5639483	-3.9190417	-3.0358195
H	4.6155360	-3.7681937	-1.2749169
H	3.6277180	-5.0283726	-2.0230383
C	2.3209938	-4.3249749	1.6337594
H	3.3538147	-4.4514064	1.2997868
H	2.2961533	-3.4800568	2.3266577
H	2.0269007	-5.2221746	2.1860096
O	0.6952560	1.9365974	0.6507006

C	-0.5567316	2.4451526	1.1608177
C	1.7717111	2.8575659	1.0009636
C	-0.3442890	3.9437093	1.2905701
H	-1.3395160	2.1571298	0.4615749
H	-0.7652560	1.9829264	2.1292297
C	1.1123847	4.0055097	1.7641808
H	2.5140897	2.3185165	1.5860992
H	2.2424463	3.1805825	0.0730275
H	-1.0437573	4.4048090	1.9876502
H	-0.4550818	4.4279899	0.3175567
H	1.1651913	3.8290985	2.8401825
H	1.5933974	4.9605308	1.5549599
O	3.0242981	0.6323624	-0.4819531
O	0.4090634	-0.7650164	1.9514126
C	3.6735300	0.9798969	-1.5816849
C	5.0826411	0.8441997	-1.6922153
C	2.9443959	1.4428210	-2.7013437
C	5.6583061	1.0577013	-2.9380893
C	3.5821740	1.6629004	-3.9214806
C	4.9442697	1.4508595	-4.0721967
H	6.7179843	0.8763009	-3.0504818
H	2.9777067	1.9835929	-4.7609921
C	-0.7125001	-0.6508428	2.6033531
C	-0.7292430	-0.4874688	4.0229652
C	-1.9665590	-0.6859360	1.9153070
C	-1.9539402	-0.4988539	4.6629663
C	-3.1788607	-0.6553871	2.6391228
C	-3.1968635	-0.5771467	4.0109186
H	-1.9686476	-0.4052525	5.7388443
H	-4.1021279	-0.6843005	2.0744700
C	-2.0905682	-0.7026001	0.4779760
H	-3.1239344	-0.7746591	0.1212011
C	1.4558324	1.6516412	-2.5994436
H	1.0942285	2.1139133	-3.5243771
H	1.2071376	2.3240728	-1.7764243
N	0.6998262	0.3910968	-2.3406672
N	-1.1551070	-0.6088069	-0.4008066
C	-0.7592321	0.5552999	-2.4984344
H	-1.0451191	0.6235568	-3.5536205
H	-1.0377863	1.4965667	-2.0199079
C	-1.5049701	-0.5891586	-1.8170810
H	-2.5841292	-0.4821501	-1.9755986
H	-1.1944090	-1.5401700	-2.2633236
H	1.0279243	-0.3042341	-3.0072362
C	-4.4683633	-0.5348754	4.8679930
C	5.6944519	1.5810436	-5.4038713
C	5.9397804	0.3449622	-0.5077819
C	0.5824483	-0.3347435	4.8269723
C	4.7531844	1.9104440	-6.5804618
H	5.3304380	1.9652168	-7.5050261
H	3.9871210	1.1447954	-6.7102061
H	4.2598619	2.8738517	-6.4321117
C	6.7119373	2.7339042	-5.2825465
H	6.1847272	3.6495857	-5.0094036
H	7.4586220	2.5345581	-4.5134358
H	7.2298963	2.9157321	-6.2269802
C	5.6151699	1.1014794	0.8063116
H	5.6761204	2.1822326	0.6759679
H	4.6104678	0.8656014	1.1423496
H	6.3301789	0.8117141	1.5791842
C	5.6185383	-1.1470344	-0.2881808
H	4.5641964	-1.2619875	-0.0472394

H	5.8149653	-1.7305896	-1.1867372
H	6.2067664	-1.5601821	0.5350884
C	1.5089839	0.7484812	4.2117747
H	1.8767649	0.4384812	3.2396189
H	0.9888268	1.6985966	4.0886317
H	2.3591118	0.9154924	4.8754468
C	1.3315456	-1.6809270	4.7841029
H	0.7354182	-2.4853239	5.2174128
H	1.5395991	-1.9391099	3.7488660
H	2.2806470	-1.6206113	5.3213314
C	-5.7473830	-0.4088608	4.0168923
H	-5.7329717	0.4859198	3.3931140
H	-5.8772169	-1.2792426	3.3699138
H	-6.6169925	-0.3453311	4.6727570
C	-4.5582000	-1.8578127	5.6554721
H	-3.7029900	-1.9948276	6.3187039
H	-5.4696309	-1.9044958	6.2549726
H	-4.5736068	-2.6932051	4.9537627
C	-4.3869708	0.6955353	5.7897721
C	-4.5500993	0.6275313	7.1723396
C	-4.1337151	1.9517679	5.2250551
C	-4.4637397	1.7714683	7.9649627
H	-4.7349877	-0.3215185	7.6553876
C	-4.0513226	3.0942169	6.0079235
H	-3.9781959	2.0289395	4.1555034
C	-4.2161979	3.0091685	7.3887201
H	-4.5831464	1.6858912	9.0382609
H	-3.8504965	4.0521309	5.5428563
H	-4.1435111	3.8967623	8.0050837
C	6.3661927	0.2315168	-5.7158811
C	7.6892014	0.1186250	-6.1406103
C	5.6168886	-0.9456758	-5.5954608
C	8.2470307	-1.1249348	-6.4365954
H	8.3083898	0.9993567	-6.2385173
C	6.1655318	-2.1850348	-5.8914462
H	4.5962952	-0.8879866	-5.2375346
C	7.4894646	-2.2819632	-6.3155347
H	9.2801774	-1.1831030	-6.7581257
H	5.5631727	-3.0792856	-5.7825003
H	7.9236051	-3.2485121	-6.5403752
C	0.2788936	0.1129873	6.2670497
C	0.5478022	-0.6801200	7.3814181
C	-0.3021677	1.3685486	6.4946089
C	0.2520433	-0.2397446	8.6720653
H	0.9888748	-1.6591192	7.2618308
C	-0.6091210	1.8079550	7.7731692
H	-0.5613742	1.9976564	5.6527080
C	-0.3289175	1.0037664	8.8751667
H	0.4744991	-0.8807629	9.5169156
H	-1.0848153	2.7711151	7.9076449
H	-0.5686224	1.3418376	9.8758040
C	7.4320187	0.5615232	-0.8089283
C	7.9509377	1.8623499	-0.8499296
C	8.3110847	-0.4872121	-1.0784077
C	9.2851151	2.1045828	-1.1446237
H	7.2935508	2.7039026	-0.6756826
C	9.6528564	-0.2518176	-1.3751872
H	7.9589851	-1.5083932	-1.0741647
C	10.1482874	1.0439393	-1.4096977
H	9.6516468	3.1239385	-1.1731852
H	10.3064666	-1.0900092	-1.5857598
H	11.1897464	1.2287091	-1.6433321

fac-fac YL5(THF)(bdsa)

147

Y	0.3605285	1.9543616	2.2245264
N	0.3915549	3.1287999	4.1652463
Si	-1.1213061	3.6396778	4.7625302
H	-2.1602863	3.0892725	3.8268720
Si	1.9157094	3.3624498	4.8944944
H	2.9457875	2.9777995	3.8770838
C	-1.3547119	5.5140392	4.7310676
H	-1.1433930	5.9322301	3.7430281
H	-2.3798127	5.7843537	5.0001147
H	-0.6812831	6.0033927	5.4392163
C	2.2054713	2.2450287	6.3853947
H	3.2296048	2.3337631	6.7593909
H	1.5239196	2.4974475	7.2020600
H	2.0267235	1.1995394	6.1203733
C	2.2845055	5.1414039	5.3940910
H	3.3201989	5.2438352	5.7301052
H	2.1296049	5.8237219	4.5548827
H	1.6348710	5.4641247	6.2123856
C	-1.5269132	3.0340844	6.4977131
H	-1.3751666	1.9567839	6.5821406
H	-0.8820495	3.5238482	7.2329618
H	-2.5645714	3.2559801	6.7609314
O	1.9603122	2.6792793	0.9295326
O	-1.0878699	0.4043674	2.7702352
C	2.1059342	3.4816898	-0.1139470
C	2.9144479	3.1558766	-1.2316971
C	1.3859623	4.7046106	-0.1373555
C	3.0073081	4.0831119	-2.2750569
C	1.4866568	5.5666277	-1.2162503
C	2.3062684	5.2827206	-2.3068057
H	3.6411942	3.8335090	-3.1098269
H	0.8862990	6.4702352	-1.2183309
C	-1.6229917	-0.6687235	2.2645257
C	-2.0165821	-1.7651963	3.0857959
C	-1.8044564	-0.7884072	0.8508043
C	-2.3820302	-2.9522486	2.4607498
C	-2.2330997	-1.9999344	0.2925391
C	-2.4762123	-3.1143451	1.0728164
H	-2.6025221	-3.7984542	3.0910897
H	-2.3237294	-2.0649117	-0.7867186
C	-1.6000713	0.3282004	-0.0468333
H	-2.0568031	0.2066676	-1.0356436
C	0.4590737	5.0362360	0.9991580
H	0.0399172	6.0338860	0.8351823
H	0.9982888	5.0528216	1.9500236
N	-0.6497659	4.0474666	1.1705886
N	-0.9766965	1.4209782	0.2116588
C	-1.4511628	3.7875632	-0.0506336
H	-1.4000393	4.6298577	-0.7470937
H	-2.4907750	3.6676798	0.2587428
C	-1.0041305	2.5060269	-0.7606573
H	-1.6831583	2.2957917	-1.5951820
H	-0.0009813	2.6434653	-1.1664383
C	-2.8129127	-4.4569968	0.4058247
C	-1.9504385	-1.6604727	4.6276101
C	3.7126385	1.8343776	-1.2902424

C	2.3451612	6.2550792	-3.4944417
C	2.8223260	0.6101046	-0.9506557
H	2.5175462	0.6458096	0.0875189
H	1.9203673	0.5840104	-1.5623176
H	3.3806510	-0.3111354	-1.1321955
C	4.8284977	1.9212268	-0.2297614
H	5.5109341	2.7466000	-0.4358604
H	4.3762300	2.1009088	0.7430759
H	5.4061511	0.9947229	-0.1812276
C	3.2072939	5.7288675	-4.6610071
H	3.1741535	6.4405048	-5.4878719
H	4.2495828	5.6121048	-4.3568463
H	2.8496777	4.7680132	-5.0317584
C	2.9672622	7.5843593	-3.0224337
H	3.0673216	8.2952612	-3.8457094
H	2.3771708	8.0529762	-2.2338792
H	3.9641205	7.3910741	-2.6235801
C	-2.6837397	-0.3869744	5.1227166
H	-2.1803511	0.5046688	4.7638574
H	-3.7144974	-0.3524027	4.7710566
H	-2.6971402	-0.3743661	6.2143681
C	-0.4732840	-1.5453177	5.0633680
H	0.1145014	-2.4028088	4.7304610
H	-0.0365513	-0.6464289	4.6349568
H	-0.3955838	-1.4779359	6.1508652
C	-4.1571890	-4.3156249	-0.3357970
H	-4.9257881	-4.0015911	0.3717004
H	-4.1085785	-3.5703688	-1.1311796
H	-4.4735787	-5.2637362	-0.7752104
C	-2.9676328	-5.6009630	1.4302490
H	-3.1748794	-6.5333262	0.9025480
H	-2.0608277	-5.7462786	2.0185466
H	-3.7951162	-5.4079160	2.1157294
H	-1.2741543	4.4311856	1.8725200
O	1.8670343	0.0702422	2.5162183
C	1.5994995	-1.2786054	2.0269924
C	3.2673027	0.1915691	2.9029533
C	2.8396363	-2.0873893	2.3751722
H	0.6930280	-1.6359492	2.5030528
H	1.4356524	-1.2202292	0.9490593
C	3.9526745	-1.0349449	2.3229533
H	3.6337022	1.1369578	2.5119574
H	3.3117583	0.2108500	3.9937978
H	2.9941096	-2.9110090	1.6787424
H	2.7506793	-2.5009787	3.3821437
H	4.2533729	-0.8472841	1.2915929
H	4.8361716	-1.3158112	2.8952491
C	4.2784803	1.5887958	-2.6985208
C	5.6437256	1.5920312	-2.9814953
C	3.4017958	1.3389379	-3.7640201
C	6.1187685	1.3536332	-4.2716476
H	6.3628182	1.7852494	-2.1985967
C	3.8661389	1.1062032	-5.0502748
H	2.3341492	1.3545640	-3.5881031
C	5.2345802	1.1102151	-5.3127231
H	7.1864623	1.3631698	-4.4562878
H	3.1595804	0.9248458	-5.8514729
H	5.6016230	0.9283021	-6.3152484
C	-2.6336007	-2.8762639	5.2768988
C	-4.0237714	-3.0284033	5.1818727
C	-1.9279457	-3.8692679	5.9566406
C	-4.6774158	-4.1179137	5.7397914

H	-4.6050955	-2.2936351	4.6409284
C	-2.5781557	-4.9656637	6.5227539
H	-0.8539857	-3.8057634	6.0548973
C	-3.9556315	-5.0970108	6.4181648
H	-5.7531127	-4.2043476	5.6434400
H	-1.9983910	-5.7175966	7.0447971
H	-4.4620129	-5.9483630	6.8560252
C	-1.6544255	-4.8330142	-0.5344797
C	-0.3415987	-4.7888333	-0.0475815
C	-1.8430530	-5.2451813	-1.8526846
C	0.7381407	-5.1426267	-0.8434364
H	-0.1662971	-4.4575332	0.9688698
C	-0.7609707	-5.5988227	-2.6590666
H	-2.8378905	-5.2932945	-2.2732337
C	0.5335469	-5.5500102	-2.1603746
H	1.7425162	-5.0972581	-0.4392765
H	-0.9372336	-5.9110252	-3.6815383
H	1.3738423	-5.8219964	-2.7870779
C	0.9093655	6.4281238	-4.0199342
C	0.1515644	5.2862124	-4.3097644
C	0.3183627	7.6712598	-4.2412886
C	-1.1411897	5.3813950	-4.8038932
H	0.5810038	4.3088235	-4.1266048
C	-0.9832920	7.7736682	-4.7334481
H	0.8626555	8.5806676	-4.0279007
C	-1.7191319	6.6316721	-5.0182396
H	-1.7026110	4.4797418	-5.0187480
H	-1.4184236	8.7534364	-4.8908418
H	-2.7297370	6.7104222	-5.3997744

mer-fac YL5(THF)(bdsa)

147

Y	1.0310274	-0.2102540	-0.1779135
N	1.5063976	-2.4370965	-0.5901661
Si	0.8944352	-3.6822768	0.4076509
H	1.7423781	-3.8618857	1.6347023
Si	2.6622149	-2.7122125	-1.8225075
H	3.1406028	-1.3805786	-2.2964458
C	0.8038111	-5.3867429	-0.4003739
H	1.7797871	-5.7421911	-0.7368862
H	0.4141460	-6.1153568	0.3165326
H	0.1349896	-5.3748635	-1.2651011
C	1.9591873	-3.5590463	-3.3591617
H	2.7015675	-3.5816742	-4.1625624
H	1.6620747	-4.5884131	-3.1460736
H	1.0758491	-3.0348042	-3.7351163
C	4.2001990	-3.6573656	-1.2809304
H	4.9375144	-3.6788213	-2.0887075
H	4.6659066	-3.1759701	-0.4184515
H	3.9712519	-4.6904128	-1.0073467
C	-0.8489211	-3.3203038	1.0385825
H	-0.9635105	-2.3398886	1.5040361
H	-1.5677645	-3.3726953	0.2169408
H	-1.1299224	-4.0727274	1.7810496
O	1.7922635	-0.7998795	2.0675095
C	0.9933990	-1.0225137	3.2602370
C	3.1741436	-1.1877739	2.3269740
C	1.9952407	-0.9831488	4.3980452

H	0.2293004	-0.2512171	3.2920172
H	0.5192255	-2.0034854	3.1865408
C	3.2060261	-1.6843963	3.7697009
H	3.4513669	-1.9482241	1.5985923
H	3.7888086	-0.3005180	2.1803902
H	1.6239214	-1.4862932	5.2903305
H	2.2347087	0.0506939	4.6533590
H	3.0729721	-2.7670564	3.7995248
H	4.1470137	-1.4391947	4.2610745
O	2.5997961	1.1080148	-0.8000356
O	-0.1660763	1.1329975	1.0674325
C	3.3088184	1.3115113	-1.9056209
C	4.7266738	1.3525427	-1.9009765
C	2.6336447	1.4153596	-3.1409080
C	5.3776787	1.3326223	-3.1287314
C	3.3395679	1.4203712	-4.3422347
C	4.7247638	1.3393560	-4.3617322
H	6.4566499	1.2599598	-3.1292347
H	2.7742763	1.4493489	-5.2653093
C	-1.3302499	1.2217451	1.6383233
C	-1.5582909	2.1245140	2.7264001
C	-2.4279359	0.4312258	1.1793578
C	-2.7627451	2.0269291	3.3970987
C	-3.6588216	0.4444709	1.8693900
C	-3.8308113	1.1951755	3.0081955
H	-2.9230080	2.6636172	4.2548920
H	-4.4644861	-0.1621879	1.4752370
C	-2.3682403	-0.2993163	-0.0623775
H	-3.3426824	-0.6139102	-0.4528765
C	1.1310003	1.4000063	-3.1571574
H	0.7737773	1.6273493	-4.1673412
H	0.7214124	2.1582239	-2.4826475
N	0.5942859	0.0853544	-2.7055956
N	-1.3175868	-0.5712649	-0.7532556
C	-0.8250428	-0.1234725	-3.0609413
H	-0.9353185	-0.4587720	-4.0979445
H	-1.3386414	0.8352652	-2.9660482
C	-1.4681527	-1.1175527	-2.0945770
H	-2.5179567	-1.2821793	-2.3637789
H	-0.9398947	-2.0739076	-2.1463475
H	1.1508016	-0.6337324	-3.1595101
C	-5.1094762	1.2092730	3.8560044
C	5.5603230	1.1676282	-5.6368825
C	5.5507819	1.2204709	-0.6035385
C	-0.4938368	3.1730749	3.1303782
C	4.6883375	1.1021851	-6.9069028
H	5.3257655	0.9565049	-7.7805384
H	3.9816222	0.2717563	-6.8686764
H	4.1272235	2.0291291	-7.0471482
C	6.5068378	2.3762674	-5.7811020
H	5.9166420	3.2939118	-5.7971380
H	7.2091545	2.4451413	-4.9494163
H	7.0787483	2.3294455	-6.7100801
C	4.9805252	2.0628747	0.5663943
H	4.8984085	3.1153159	0.2930527
H	3.9914004	1.7201974	0.8523381
H	5.6539883	1.9894287	1.4234951
C	5.5006329	-0.2677564	-0.2100849
H	4.4621346	-0.5753076	-0.1250124
H	5.9639797	-0.8950227	-0.9719854
H	5.9933750	-0.4511335	0.7483183
C	-0.0336673	3.9800936	1.8860581

H	0.4925021	3.3370173	1.1859929
H	-0.8787180	4.4307756	1.3656287
H	0.6358261	4.7824850	2.2025986
C	0.7472707	2.4614972	3.7034606
H	0.4951438	1.8493759	4.5710719
H	1.1789161	1.8172265	2.9419974
H	1.5049608	3.1888758	4.0022847
C	-6.2650998	0.4373576	3.1882670
H	-6.5120048	0.8439577	2.2066911
H	-6.0155922	-0.6195585	3.0711133
H	-7.1572185	0.5046356	3.8126140
C	-4.7951348	0.5112741	5.1948752
H	-4.0086241	1.0284926	5.7459882
H	-5.6806886	0.4514605	5.8311586
H	-4.4524161	-0.5059701	4.9987182
C	-5.5598515	2.6688599	4.0451077
C	-5.7963908	3.2406829	5.2941707
C	-5.7448229	3.4742208	2.9147200
C	-6.2048733	4.5687050	5.4128772
H	-5.6551741	2.6628019	6.1965822
C	-6.1569605	4.7942893	3.0261563
H	-5.5423671	3.0629825	1.9331475
C	-6.3901158	5.3507680	4.2819085
H	-6.3692918	4.9895686	6.3974475
H	-6.2882953	5.3936309	2.1331848
H	-6.7022742	6.3837358	4.3739620
C	6.3193548	-0.1687263	-5.5279269
C	7.6714421	-0.3048458	-5.8387878
C	5.6269801	-1.3150544	-5.1165315
C	8.3131027	-1.5392973	-5.7402315
H	8.2485732	0.5534454	-6.1539335
C	6.2590388	-2.5459789	-5.0198623
H	4.5832078	-1.2321276	-4.8400787
C	7.6119497	-2.6655497	-5.3313784
H	9.3670743	-1.6133516	-5.9806281
H	5.6986639	-3.4132816	-4.6910008
H	8.1113360	-3.6232376	-5.2487472
C	-1.0862319	4.1747186	4.1372346
C	-0.6545620	4.2644723	5.4604903
C	-2.1182049	5.0358157	3.7384164
C	-1.2235498	5.1765535	6.3495313
H	0.1333692	3.6205109	5.8233755
C	-2.6961220	5.9368790	4.6195380
H	-2.5073561	4.9727712	2.7307258
C	-2.2481500	6.0154558	5.9355977
H	-0.8623439	5.2213946	7.3701760
H	-3.5109294	6.5651815	4.2832113
H	-2.6987214	6.7163269	6.6274586
C	6.9911673	1.7097786	-0.8226698
C	7.2155945	3.0389959	-1.2057560
C	8.1080570	0.8970916	-0.6355041
C	8.4977209	3.5339420	-1.3932137
H	6.3681276	3.6888469	-1.3846104
C	9.4005649	1.3889926	-0.8187977
H	7.9889716	-0.1380026	-0.3489990
C	9.6033042	2.7081489	-1.1978840
H	8.6363102	4.5651697	-1.6958041
H	10.2476257	0.7306427	-0.6665724
H	10.6061967	3.0903633	-1.3435006

6 References

(1) Wang, Z.; He, J.; Mu, Y. Synthesis of chiral salan ligands with bulky substituents and their application in Cu-catalyzed asymmetric Henry reaction. *J. Organomet. Chem.* **2020**, *928*, 121546. DOI: https://doi.org/10.1016/j.jorganchem.2020.121546.

(2) Du, H.; Pang, X.; Yu, H.; Zhuang, X.; Chen, X.; Cui, D.; Wang, X.; Jing, X. Polymerization of rac-Lactide Using Schiff Base Aluminum Catalysts: Structure, Activity, and Stereoselectivity. *Macromolecules* **2007**, *40* (6), 1904-1913. DOI: 10.1021/ma062194u.

(3) Bruckmoser, J.; Pongratz, S.; Rieger, B. High-Throughput Approach in the Ring-Opening Polymerization of β-Butyrolactone Enables Rapid Evaluation of Yttrium Salan Catalysts. *Organometallics* **2023**, *42* (10), 876-884. DOI: 10.1021/acs.organomet.2c00624.

(4) Altenbuchner, P. T.; Kronast, A.; Kissling, S.; Vagin, S. I.; Herdtweck, E.; Pöthig, A.; Deglmann, P.; Loos, R.; Rieger, B. Mechanistic Investigations of the Stereoselective Rare Earth Metal-Mediated Ring-Opening Polymerization of β-Butyrolactone. *Chemistry – A European Journal* **2015**, *21* (39), 13609-13617. DOI: https://doi.org/10.1002/chem.201501156.

(5) Tang, X.; Chen, E. Y. X. Chemical synthesis of perfectly isotactic and high melting bacterial poly(3-hydroxybutyrate) from bio-sourced racemic cyclic diolide. *Nature Communications* **2018**, *9* (1), 2345. DOI: 10.1038/s41467-018-04734-3.

(6) Wang, Y.; Qin, Y.; Wang, X.; Wang, F. Coupling reaction between CO2 and cyclohexene oxide: selective control from cyclic carbonate to polycarbonate by ligand design of salen/salalen titanium complexes. *Catalysis Science & Technology* **2014**, *4* (11), 3964-3972, 10.1039/C4CY00752B. DOI: 10.1039/C4CY00752B.

(7) Gesslbauer, S.; Savela, R.; Chen, Y.; White, A. J. P.; Romain, C. Exploiting Noncovalent Interactions for Room-Temperature Heteroselective rac-Lactide Polymerization Using Aluminum Catalysts. *ACS Catalysis* **2019**, *9* (9), 7912-7920. DOI: 10.1021/acscatal.9b00875.

(8) Bruckmoser, J.; Pongratz, S.; Stieglitz, L.; Rieger, B. Highly Isoselective Ring-Opening Polymerization of rac-β-Butyrolactone: Access to Synthetic Poly(3-hydroxybutyrate) with Polyolefin-like Material Properties. *Journal of the American Chemical Society* **2023**, *145* (21), 11494-11498. DOI: 10.1021/jacs.3c02348.

(9) Bachmann, S.; Gernert, B.; Stalke, D. Solution structures of alkali metal cyclopentadienides in THF estimated by ECC-DOSY NMR-spectroscopy (incl. software). *Chem. Commun.* **2016**, *52* (87), 12861-12864, 10.1039/C6CC07273A. DOI: 10.1039/C6CC07273A.

(10) Becke, A. D. Density-functional thermochemistry. III. The role of exact exchange. *The Journal of Chemical Physics* **1993**, *98* (7), 5648-5652. DOI: 10.1063/1.464913 (acccessed 2/5/2025).

(11) Grimme, S.; Antony, J.; Ehrlich, S.; Krieg, H. A consistent and accurate ab initio parametrization of density functional dispersion correction (DFT-D) for the 94 elements H-Pu. *The Journal of Chemical Physics* **2010**, *132* (15). DOI: 10.1063/1.3382344 (acccessed 2/5/2025).

(12) Weigend, F.; Ahlrichs, R. Balanced basis sets of split valence, triple zeta valence and quadruple zeta valence quality for H to Rn: Design and assessment of accuracy. *Physical Chemistry Chemical Physics* **2005**, *7* (18), 3297-3305, 10.1039/B508541A. DOI: 10.1039/B508541A.

(13) Andrae, D.; Häußermann, U.; Dolg, M.; Stoll, H.; Preuß, H. Energy-adjustedab initio pseudopotentials for the second and third row transition elements. *Theoretica chimica acta* **1990**, *77* (2), 123-141. DOI: 10.1007/BF01114537.

(14) Eichkorn, K.; Weigend, F.; Treutler, O.; Ahlrichs, R. Auxiliary basis sets for main row atoms and transition metals and their use to approximate Coulomb potentials. *Theor. Chem. Acc.* **1997**, *97* (1), 119-124. DOI: 10.1007/s002140050244.

(15) Klamt, A.; Schüürmann, G. COSMO: a new approach to dielectric screening in solvents with explicit expressions for the screening energy and its gradient. *JACS* **1993**, (5), 799-805, 10.1039/P29930000799. DOI: 10.1039/P29930000799.
(16) Miertuš, S.; Scrocco, E.; Tomasi, J. Electrostatic interaction of a solute with a continuum. A direct utilizaion of AB initio molecular potentials for the prevision of solvent effects. *Chem. Phys.* **1981**, *55* (1), 117-129. DOI: https://doi.org/10.1016/0301-0104(81)85090-2.
(17) Tomasi, J.; Persico, M. Molecular Interactions in Solution: An Overview of Methods Based on Continuous Distributions of the Solvent. *Chem. Rev.* **1994**, *94* (7), 2027-2094. DOI: 10.1021/cr00031a013.
(18) Ahlrichs, R.; Bär, M.; Häser, M.; Horn, H.; Kölmel, C. Electronic structure calculations on workstation computers: The program system turbomole. *Chem. Phys. Lett.* **1989**, *162* (3), 165-169. DOI: https://doi.org/10.1016/0009-2614(89)85118-8.
(19) Franzke, Y. J.; Holzer, C.; Andersen, J. H.; Begušić, T.; Bruder, F.; Coriani, S.; Della Sala, F.; Fabiano, E.; Fedotov, D. A.; Fürst, S.; et al. TURBOMOLE: Today and Tomorrow. *Journal of Chemical Theory and Computation* **2023**, *19* (20), 6859-6890. DOI: 10.1021/acs.jctc.3c00347.
